# Pisiform Homojunction with Energy Band Bending Induced via Co-Implantation Design Enabling Fast-Charging Sodium-Sulfur Battery

**DOI:** 10.1007/s40820-026-02163-2

**Published:** 2026-03-27

**Authors:** Yanjun Gao, Zujia Lu, Qiyao Yu, Jianguo Zhang

**Affiliations:** State Key Laboratory of Explosion Science and Safety Protection, Beijing Institute of Technology, Beijing, 100081 People’s Republic of China

**Keywords:** Induced-homojunction, Co-implantation design, Fast conversion reaction kinetics, High utilization efficiency, Fast-charging ability

## Abstract

A novel “induced-homojunction” concept proposed here is of great significance to alleviate the severe shuttling effects and poor rate-capability behavior, where the sandwiched p-n Mo_2_C homojunction/carbon composite is constructed by the co-implantation design of Fe and Mo-vacancy (v_6_Fe–Mo_2_C/C), enabling heterogeneous variation in n/p-type characteristics among adjacent crystal structure. Encouringly, the p-n homojunction formation with continuous band bending favors the rapid carrier transmission across the interface to endow reactive sites with high activity and strengthen polysulfides capture, hence promoting original S–S bonds cleavage, which is regarded as the critical step to suppress the shuttling-behavior and trigger conversion reactions occurrence. Crucially, high-speed ions/electrons transport effectively driven by the formed large-range internal-electric-field during the energy band alignment, ensures they timely reach the above-mentioned highly active sites and react fully, enabling the ultrafast conversion kinetics process. A conspicuous sulfur utilization (1508 mAh g^−1^ at 0.1 A g^−1^) and especially the superior rate performance (1337 mAh g^−1^ at 1 A g^−1^) are presented by the battery with v_6_Fe–Mo_2_C/C@S cathode. And the battery delivers a stable discharge capacity independently from the charging rate (even at 5 A g^−1^). This “induced-homojunction” concept achieves the significant reaction kinetics advantage to provide new insight for the exploitation of fast-charging Na–S batteries.

**Supplementary Information:**

The online version contains supplementary material available at 10.1007/s40820-026-02163-2.

## Highlights

- A novel “induced-homojunction” concept is first applied to achieve fast-charging Na–S batteries.
- Homojunction interface regulation enables high-efficiency NaPSs capture and fast conversion reaction kinetics.
- Na–S battery shows high specific capacity and superior rate performance with ultrahigh capacity retention up to 88.8% at 1 A g^−1^. And the battery delivers a stable discharge capacity independently from the charging rate (even at 5 A g^−1^).

**Supplementary Information:**

The online version contains supplementary material available at 10.1007/s40820-026-02163-2.

## Graphical abstract

**Figure:**
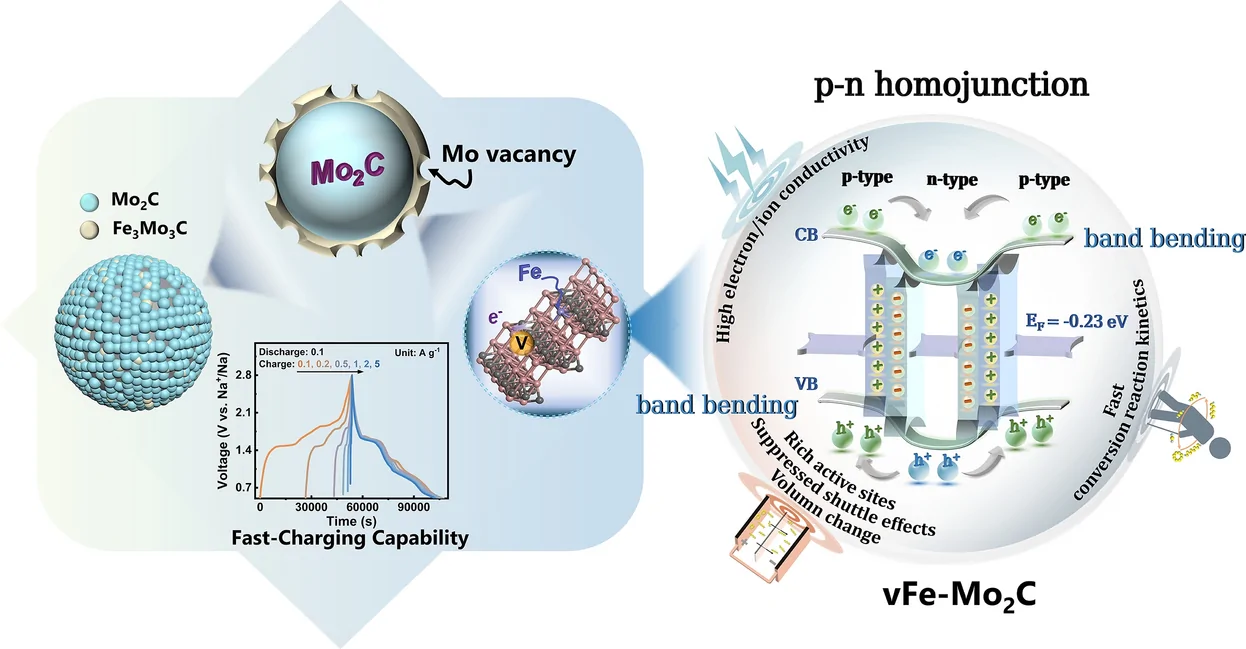

## Introduction

Reliable and cost-effective energy is vital for modern society’s development. The exponentially growing demand for future high-energy electronic systems represented by electric vehicles (EVs), unmanned deep-space drones and flexible smart power energy storage devices strongly stimulates the exploitation of advanced rechargeable next-generation batteries [1–3]. In contrast to lithium resources with geological scarcity, sodium’s inherent abundance merit ensures a sustainable supply chain capable of meeting the development of sodium-based battery technologies. Particularly, rechargeable room-temperature (RT) Na–S batteries further reduce the high cost of electrode elements by using inexpensive and environmentally friendly sulfur as the active material and then circumvent the bottlenecks of critical material scarcity to be appropriate for gigawatt-scale stationary energy storage in grid applications. It has also opened up a new avenue toward the energy storage field due to the high theoretical capacity up to 1675 mAh g^−1^ (S), ultrahigh specific energy density of 1274 Wh kg^−1^, cost-efficiency and non-toxicity. Yet now, the sulfur cathode suffers from poor electron conductivity (5 × 10^−28^ S m^−1^ at 25 °C) and huge volume change during cycling. More unfortunately, inevitable dissolution interactions between sodium polysulfides (NaPSs) and the solvent molecules of liquid electrolyte (LE) bring about the diffusion of the active materials from cathode to anode by shuttling. Integrating with the sluggish solid–liquid-solid phase transformation from sulfur to high-order NaPSs and subsequently to the final Na_2_S_2_/Na_2_S product, the low sulfur utilization and poor cycling stability are all unavoidable [4–8].

Noteworthy, the exploitation of host materials has been demonstrated to be an effective approach to fully utilize the sulfur cathode by solving the aforementioned drawbacks. As for the volume variation issue of electrodes, strategies such as introducing porous carbon architectures and depositing functional coatings on electrodes have been proven to be effective [9–12]. Carbon materials used to enhance electronic conductivity and even enhance the mechanical stability have also been widely employed. Li et al. [13] proposed a three-dimensional porous graphitic carbon composite containing sulfur (3D S@PGC) as the cathode, where the 3D PGC network endows the electrode with high electroconductivity and structural integrity. Liu’s group [14] used a flexible carbon cloth (CC) deposited by Ni layer (CC/Ni) that can provide high-speed electron-transport pathways and robust mechanical support, together with the hollow CoS nanocage structure, to achieve an ultrahigh areal capacity in lithium-ion battery (LIB). Therefore, constructing carbon/sulfur cathodes is considered to compensate for the poor conductivity of sulfur and mitigate the shuttling effects through physical confinement. To date, a variety of carbon hosts, including carbon nanotubes (CNTs) and metal–organic-framework (MOF)-derived carbon, have been widely reported [15–17]. However, the problem that inherently weak affinity between nonpolar carbon and polar NaPSs is innegligible. Given the energy storage mechanism of sulfur active species, introducing highly efficient electrocatalytic matrices usually with polar immobilizing functions can not only enhance the anchoring capability toward NaPSs but also markedly reduce the energy barriers of redox reactions. This dual functionality addresses the drawback of incomplete conversion processes toward solid Na_2_S, shortens the residence time of soluble NaPSs in the electrolyte, thereby enabling full realization of the capacity [10, 18–20]. After tuning the elemental composition, Chen et al. [21] constructed the high-entropy sulfides incorporating Ti, Ni, Co, Fe, and Mg, which served as the sulfur host to fully exert their catalytic activity on conversion reactions involved in Li–S batteries, finally delivering the prolonged cycling life. A modified-spinel electrocatalyst (Fe_0.1_Co_2.9_O_4_–Se) by complementing exclusive Se–O coordination and Fe-doping was designed by Wen et al. [22], which facilitated the instantaneous nucleation of Li_2_S, lowered the bidirectional catalytic energy barriers, and thereby presenting the excellent catalytic ability. Thereinto, molybdenum carbide (Mo_2_C), featured eminent catalytic performance close to noble metals across a certain range of electrochemical reactions, outstanding thermal/chemical stability, tunable electronic structure and strong sulfophilic property, has been widely investigated in energy storage systems. Nonetheless, Mo_2_C as a semiconductor would inevitably impede electron transport to some extent, ultimately causing low rate capabilities and sulfur utilization [23].

Metal-atom doping can be a feasible route to prompt charge distribution distortion on the surface atoms [24–27]. Particularly, iron-doping can effectively tailor the electronic structure and introduce additional active sites with highly catalytic activity, which offers a promising route to improve the intrinsic conductivity of materials. Fe-doping has been widely demonstrated as a reliable and well-established strategy in recent studies across electrocatalysis and electrochemical energy storage fields [28, 29]. The Fe-doped NiSe_2_ particle was reported by Cabot et al. [30], and the related effects were studied, where the Fe-rich cores and doping in NiSe_2_ strengthened the density of states at the Fermi level and introduced unpaired electrons facilitating S–S bond breakage, thereby improving the adsorption behavior and accelerating the catalytic conversion rate of lithium polysulfides (LiPSs). Hollow and hierarchical nanoplates composed of Fe-doped Co_3_O_4_ nanosheets have also been proposed as sulfur hosts [31]. Notably, the introduction of heterogeneous Fe-atoms induced electron redistribution and local structural deformation, which contributes to reducing the energy barriers of the conversion reactions, enabling high areal capacities even at a high sulfur loading and the finite electrolyte. Likewise, the electron-transport resistance of Fe-Doped Mn_*x*_O_*y*_ anode was reduced in LIBs [32]. The accelerated OER process was attributed to optimized adsorption of the rate-determining step and the d-band center shifting closer to the Fermi level as a result of Fe-doping for Ni_3_S_2_ Electrode [33]. Besides, as a special type of defect, vacancy engineering has become a focus for boosting electrochemical performance. The presence of atomic vacancies usually provides rich active sites, optimized electronic conduction and favorable ion migration for strengthening electrocatalytic ability, typically originating from high-temperature crystal synthesis that causes random changes in the atomic arrangement [34, 35]. Zr-based cationic vacancies in Li_2_ZrO_3_ induce lattice distortion and local charge deficiency to activate the electron-compensation mechanism, thereby enhancing Li^+^ transport efficiency and markedly improving the performance of solid-state lithium-metal batteries [36]. As for the Fe_3-*x*_C material, the defective Fe is responsible for the strengthened confinement and catalytic function for LiPSs, enabling fast and durable sulfur storage processes [37]. Song and co-workers[38] achieved the stable long-cycling properties in lithium-sulfur (Li–S) batteries, attributable to the exposure of active-site with strong combining-capability by introducing abundant oxygen vacancies in the intrinsic atomic structure to stabilize the Li_2_S and immobilize polysulfides. More importantly, the homojunction design, as an interfacial band engineering, has also been proven to be an effective means to promote the charge transfer, ameliorate the inherent conductivity and accelerate the electrocatalytic reactions [39, 40]. Therefore, it holds great promise to play a critical role in accelerating charge transport, facilitating polysulfides conversion and alleviating active material losses. So far, an overwhelming majority of the researches have been concentrated on the fundamental working mechanisms that metal-atom doping and vacancy engineering synergistically offer rich active sites and increase the charge density to enhance the electron conductivity and adsorption capability for polysulfides. However, little research has linked the causal relationship between the co-existence of vacancy/dopant and homojunction to artificially create homojunction interfaces. Specifically, the corresponding mechanism that individual regulation around the doping sites and vacancies may lead to the heterogeneous variation of conductive type across neighboring structures within Mo_2_C materials to engender interfacial homojunction effects with high activity, is seldom explored, let alone the concomitant impacts on NaPSs entrapment, conversion reactions and charging/discharging rate capability.

Herein, a novel “induced Mo_2_C homojunction by co-implantation of Fe and Mo-vacancy at an atomic-scale” concept is proposed, which is distributed on N-doped carbon nanospheres, as a highly efficient sulfur host for Na-S batteries firstly. Thereinto, a simple acid-etching protocol was used to optimize the relative ratio of doped metal amounts to in-situ formed Mo-vacancy densities, which in turn maximally exerts homojunction effects. Notably, the differential regulation for electronic structure by Fe or Mo-vacancy causes the disparity of n/p conductive type and Mo_2_C energy band structure at adjacent locations. This asymmetric band configuration necessitates the homojunction interface formation, where the composition identity on both sides of the interface enables a continuous band bending, remarkably contributing to the directional carrier transport to spontaneously establish bidirectional internal electric fields (IEF). For one case, the interfacial charge accumulation at both terminals strengthens NaPSs capture, which in turn promotes the breakage of S–S bonds in NaPSs more effectually to accelerate its further transformation. More importantly, IEF affords a potent force for high-rate migration of charged species, ensuring their timely participation in conversion reactions. Consequently, the v_6_Fe–Mo_2_C/C@S cathode exhibits a highly reversible capacity of 1508 mAh g^−1^ at 0.1 A g^−1^ after 100 cycles and especially the superior rate behavior of 88.8% capacity retention at 1 A g^−1^. Its tiny capacity fading of 0.0058% per cycle is also realized over 1000 cycles. Encouragingly, this homojunction mechanism enables the outstanding fast-charging ability (5 A g^−1^) with a stable discharge capacity (1357.0 mAh g^−1^). This work not only comprehensively elaborates the mechanism of the well-designed homojunction, but also provides a new insight into enhancing the catalytic activity of sulfur host for future Na–S systems with eminent rate performance and fast-charging capability.

## Experimental Section

### Materials Synthesis of the v_t_Fe–Mo_2_C/C and Mo_2_C/C Hosts

1.23 g Ferrous chloride tetrahydrate (FeCl_2_·4H_2_O), 2.18 g Ammonium molybdate tetrahydrate ((NH_4_)_6_Mo_7_O_24_·4H_2_O) and certain amounts poly (vinyl alcohol) (PVA) were dissolved in 180 mL of deionized water, and then stirred magnetically at 85 °C for an hour to form a clear solution. Besides, a certain amount of melamine was in formaldehyde with constant stirring for 30 min at 60 °C until fully dissolved to obtain a homogeneous colorless solution. Then, the above solution and 2.4 mL of acetic acid were mixed under vigorous stirring for 4 h to form a milky solution with slight orange. Subsequently, the products were collected by centrifugation after washing with water and dried overnight. After that, calcining for the mixture was performed at 800 °C under an Ar atmosphere for two hours. The products were finally harvested after etching in 1 M HCl solution to remove the Fe species on the surface and denoted as v_t_Fe–Mo_2_C (t representing the hours of acid treatment time, including 0, 2, 4, 6, and 12 h). Furthermore, Mo_2_C/C was also synthesized by using the same procedure described above except that no iron salt was introduced into the precursor.

### Materials Synthesis of the v_t_Fe–Mo_2_C/C@S and Mo_2_C/C@S Cathodes

The corresponding cathode materials were prepared following a melt-diffusion strategy. Typically, v_t_Fe–Mo_2_C/C or Mo_2_C/C and sulfur powder were mixed via grinding with a weight ratio of 1:1, and then the mixture was sealed and then heated at 155 °C for 24 h in an Ar-filled autoclave. Afterward, further treatment at 300 °C for 10 h with a heating rate of 5 °C min^−1^ was performed to obtain the final v_t_Fe–Mo_2_C/C@S and Mo_2_C/C@S composites.

### Materials Characterization

The morphology and microstructure of samples were characterized in scanning electron microscopy (SEM) coupled with an energy dispersive X-ray spectrometer (EDS) using a Hitachi SU8600 instrument. High-resolution transmission electron microscopy (HR-TEM) characterization, including digital imaging and selected-area electron diffraction (SAED) pattern acquisition, was carried out on a Tecnai G2 F30 S-TWIN (FEI) instrument at 300 kV. XRD analysis was conducted on a Bruker D8 ADVANCE diffractometer using a Cu K*α* radiation with the 2θ degree ranging from 10 to 80°. The percentages of doped-Fe in the samples were determined by inductively coupled plasma atomic emission spectroscopy (ICP-OES 720, Agilent). The surface chemical states were analyzed by ultraviolet photoemission spectroscopy (UPS) measurements and X-ray photoelectron spectrometer (XPS) measurements on a Thermo Fisher Escalab 250Xi, using C 1*s* (284.8 eV) as a reference with an Al K*α* radiation as the photon source. The X-ray absorption fine structure spectra (XAFS) were collected at SPring-8 in fluorescence mode. Characterization of vacancies was conducted by electron paramagnetic resonance spectrometer (EPR, Bruker EMXplus-6/1). A UV–visible (UV–vis) spectrophotometer (hitachi UH4150, JAPAN) was used to record the ultraviolet–visible diffused reflectance spectra (UV–vis DRS). Brunauer–Emmett–Teller (BET) surface area and pore-size distributions were measured through nitrogen adsorption at 77 K, using an Anton Paar Autosorb 6100 (Austria) instrument. Raman spectra were characterized using Horiba LabRAM HR Evolution system with a 532 nm excitation laser. The contact angle was measured on a JY-82C Video Contact Angle Measuring Instrument. The thermal decomposition behavior of the products was monitored by using a Mettler Toledo TGA/SDTA851 analyzer under Ar with a heating rate of 5 °C min^−1^.

### Electrochemical Measurements

For the preparation of the cathodes, 70 wt% of the v_t_Fe–Mo_2_C/C@S composites were mixed with 20 wt% conductive carbon black and another 10 wt% polyvinylidene fluoride (PVDF) binder. Glass fiber separators (Whatman GF/F) and 1.0 M NaClO_4_-based electrolyte were used to assemble the CR2032 Na–S batteries in an argon-filled glove box with less than 0.01 ppm of H_2_O and O_2_. Thereinto, the average mass loading of sulfur is around 1.0 mg cm^−2^. As for the Na anode, the surface-oxidized layers need to be mechanically removed from bulk sodium, Na-metal disk is 14 mm in diameter and around 300 µm in thickness. For the battery tests, N/P ratio and E/S ratio are 20.3 and 90 μL mg^−1^, respectively [41]. High mass loading analyses are also performed, with the mass loading up to 4.1 mg cm^−2^ followed by the same procedure, corresponding to the E/S ratio of 22.0 μL mg^−1^ each cell. Before testing, the assembled batteries were aged for at least 12 h to ensure sufficient electrolyte penetration into the electrodes. The galvanostatic charge and discharge (GCD) tests were performed on the Neware battery testing system. The capacities of cells in this work were normalized based on the mass of sulfur. The cyclic voltammetry (CV) and electrochemical impedance spectra (EIS) measurements were conducted on the electrochemical workstation (CHI 660E, Shanghai Chenhua, China) with different scan rates (0.1 ~ 1 mV s^−1^) and the frequency range from 0.01 to 100 kHz, respectively. Mott-Schottky plots were also investigated at an AC frequency of 1.0 kHz with an amplitude of 0.01 V. The measured potentials were converted to the reversible hydrogen electrode (RHE) scale according to the following Nernst equation:

1 $${E}_{\mathrm{RHE}}={E}_{\mathrm{Ag}/\mathrm{AgCl}}+0.059\mathrm{pH}+{E}_{\mathrm{Ag}/\mathrm{AgCl}}^{\mathrm{o}}$$

where *E*_Ag/AgCl_ refers to the potential tested experimentally versus the Ag/AgCl reference, *E*^o^_Ag/AgCl_ = 0.1976 V at 25 °C.

Noteworthy, the diffusion coefficient of Na^+^ (D_Na+_) could be obtained from galvanostatic intermittent titration technique (GITT) measurements according to Fick’s second diffusion law as the following formula:

2 $$D_{GITT} = \frac{4}{\pi \tau }\left( {\frac{{n_{{\mathrm{M}}} V_{{\mathrm{M}}} }}{S}} \right)^{2} \left( {\frac{{{\Delta }E_{{\mathrm{S}}} }}{{{\Delta }E_{\tau } }}} \right)^{2} \left( {\tau < \; < \frac{{L^{2} }}{{D_{{{\mathrm{GITT}}}} }}} \right)$$

where τ, Δ*E*_s_ and Δ*E*_τ_ represent the relaxation time of the current pulse, the variation of the steady-state voltage and voltage variation in the range from t_0_ to t_0+τ_, respectively, *n*_M_ and *V*_M_ refer to the amount of substance and the molar volume, respectively, S is the contacting area between active material and electrolyte.

### Visualized Adsorption Test

As for the sodium polysulfide adsorption test, 20 mg of each v_t_Fe–Mo_2_C/C or Mo_2_C/C powder was added into the 10 mL diluted Na_2_S_6_ solution and kept for 12 h. All the steps were completed in the glovebox. Comparing the color of solutions and analyzing the supernatant by UV–vis absorption spectrum.

### Symmetric-Cell Assembly and Measurements

For the preparation of the electrodes, the active materials (v_t_Fe–Mo_2_C/C or Mo_2_C/C), carbon black and PVDF were mixed with a weight ratio of 8:1:1. Two identical electrodes were used as working and counter electrodes, with 25 μL 0.2 M Na_2_S_6_ electrolyte injected into each cell. The CV measurements within a voltage window of − 1.5–1.5 V with the scan rate of 5–100 mV s^−1^ and EIS tests of the symmetric cells were carried out to investigate the catalytic properties of as-synthesized materials.

### Theoretical Calculation Details

All calculations were performed by density functional theory (DFT) as implemented on the basis of Vienna Ab initio Simulation Package (VASP). The general gradient approximation (GGA) of Perdew-Burke Ernzerd of (PBE) was employed for the exchange–correlation function. The core-valence interactions were accounted by the projected augmented wave (PAW) method [42]. A vacuum space of 15 Å along the vertical direction was used to avoid mirror interactions between the adjacent cells. And a plane-wave basis set with a kinetic energy cutoff of 500 eV was used to expand the eigenstates of the electron wave functions. The Brillouin zone integration was sampled with 3 × 3 × 1 Monkhorst–Pack k-point in the Gramma-centered grids for the structure relaxation [43]. The simulation ceased until the convergence criteria of total energy (1 × 10^−5^ eV) and force tolerance (0.01 eV Å^−1^) were reached.

The adsorption energy (Eads) can be defined as follows:

3 $${E}_{\mathrm{ads}}={E}_{\left[{\mathrm{substract}+\mathrm{Na}}_{2}{S}_{x}\right]}-{E}_{\mathrm{subsract}}{-E}_{\mathrm{Na}2\mathrm{Sx}}$$

where the *E*_[substract+Na2Sx]_, *E*_substract_ and *E*_Na2Sx_ (x = 1, 2, 4, 6, and 8) represent the total energy of the host that was adsorbed by Na_2_S_x_ polysulfide, the energy of host model and the energy of polysulfides, respectively.

The Gibbs free energy (*G*_NaxSy_) can be calculated as follows:

4 $${G}_{\mathrm{NaxSy}}={E}_{\left[\mathrm{substract}+\mathrm{NaxSy}\right]}+{E}_{0}+\mathrm{nRT}-\mathrm{TS}+\left(2-\mathrm{x}\right){E}_{\mathrm{Na}}+(8-\mathrm{y}){E}_{\mathrm{S}}$$

where *G*_NaxSy_ is the Gibbs free energy, *E*_[substract+Na2Sx]_ is the total energy of the system, (*E*_0_ + nRT-TS) is the zero-point energy correction, Na is the single-point energy of sodium atom and *E*_S_ is the single-point energy of sulfur atom, respectively. Na_x_S_y_ represents polysulfide (x = 0, 2 and y = 1, 2, 4, 6, 8).

## Results and Discussion

### Design and Structure Characterizations

**Fig. 1:**
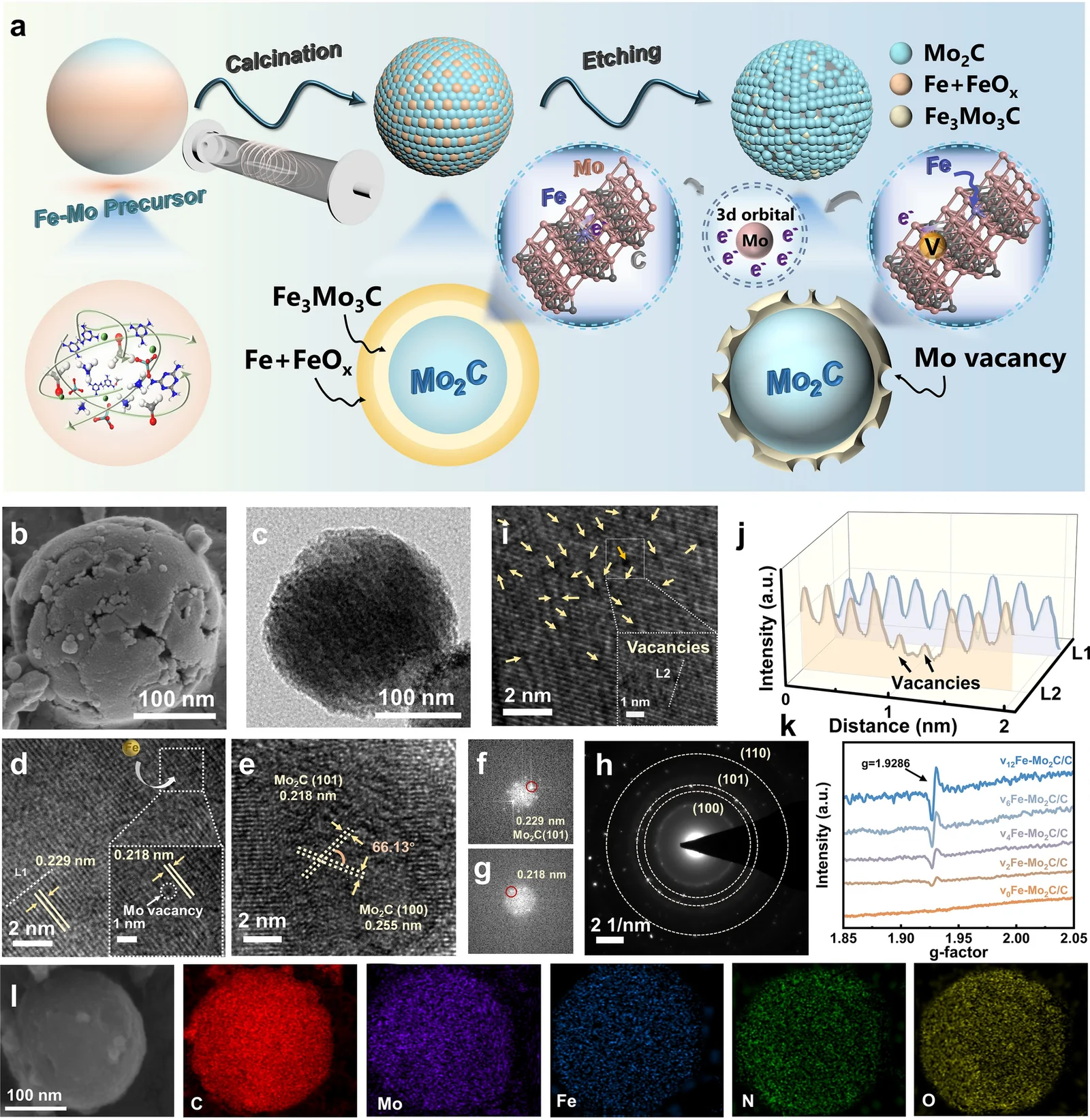
Illustration of the synthesis process and microstructure. **a** Schematic representation of the preparation of v_t_Fe–Mo_2_C/C electrocatalyst. **b** SEM image of v_6_Fe–Mo_2_C/C. **c-e** HR-TEM image with different magnification degrees of v_6_Fe–Mo_2_C/C. **f, g** Corresponding Fast Fourier transform (FFT) image of region in (**d**). **h** SAED pattern corresponding to (**e**). **i** HR-TEM images toward surface defects in v_6_Fe–Mo_2_C/C. **j** Image intensity line profiles taken along the white dotted lines (L1 and L2) in (**d**, **i**). **k** EPR spectra of v_t_Fe–Mo_2_C/C (t = 0, 2, 4, 6, and 12). **l** Corresponding EDS elemental mapping of v_6_Fe–Mo_2_C/C

To obtain a promising reservoir for sulfur with corresponding intermediates and achieve fast-charge transfer kinetics, a novel “induced-homojunction” concept via the co-implantation of Fe and Mo-vacancy at the atomic-scale is put forward. The unique structure of vacancy/Fe-doping dual-modified molybdenum carbide and carbon composite was prepared by simple one-step calcination treatment followed by the HCl etching process and denoted as v_t_Fe–Mo_2_C/C, where v and subscript t represent vacancy and the hours of acid treatment time, including 0, 2, 4, 6, and 12 h, respectively, as shown in Fig. 1a. Notedly, pre-generated bimetallic carbide and the changed HCl etching time provide the possibility to flexibly regulate the ratio of Fe-dopant amount to Mo-vacancy density in Mo_2_C materials, prompting highly active homojunction interfaces to be distributed as extensively as possible throughout the entire material [44]. The micromorphology of Mo_2_C-based carbonaceous composites was thoroughly characterized by field-emission scanning electron microscopy (FESEM), presenting an integrated spherical structure with densely dispersed particles on the carbon matrix. EDS mapping shown in Fig. 1l verifies a homogeneous distribution of Mo, C, Fe, O, and N throughout the v_6_Fe–Mo_2_C/C sample without significant aggregation. Notably, the continuously increased voids and reduced particle size in the prolonged acid treatment procedure are observed in FESEM and relevant transmission electron microscopy (TEM) patterns (Figs. 1b, c and S1c–f) on account of the elimination of Fe, FeO_*x*_ and the successful etching for the Fe–Mo bimetallic carbide, where the porous feature, especially v_6_Fe–Mo_2_C/C sample with a diameter of ≈ 240 nm, is remarkably conducive to the fast mass transport in charge/discharge reactions. However, the undue etching time as long as 12 h induces irreversible structural collapse, while no more Fe species can be removed.

To further probe the presence of Mo-vacancies and crystal-structure variation, high-resolution TEM (HR-TEM) measurements for v_6_Fe–Mo_2_C/C were performed. Particularly, the boundaries between metallic Fe species and the Mo_2_C surface are gradually exposed to the outside by HCl etching. In contrast to the perfect crystalline lattices with a typical distance of 0.229 nm that can be assigned to the (101) plane of hexagonal-phase *β*-Mo_2_C, the shrunken lattice fringes with a distance about 0.218 nm around Mo-vacancies created by acid washing Fe-doped Mo_2_C samples, mostly can be ascribed to the smaller radius of Fe-atom (124 pm) than that of Mo (139 pm) to insert into the carbide framework (Fig. 1d). Simultaneously, the inter-angle between *β*-Mo_2_C (100) and (101) plane is expanded to 66.13° than that of the standard hexagonal crystal (60°) (Fig. 1e). Apparently, analysis of the Fast Fourier transform (FFT) also indicates the hexagonal crystalline nature of Mo_2_C. Meanwhile, the revealed smaller lattice distance of 0.218 nm corresponding to (101) plane is consistent with the FE-TEM diagram (Fig. 1f, g). And the SAED analysis further supporting the polycrystalline nature of the Mo_2_C nanoparticles in view of several concentric circles, demonstrates the smaller interplanar fringes of v_6_Fe–Mo_2_C/C than that of the original *β*-Mo_2_C once again (Fig. 1h). These findings jointly reveal the pronounced change in the crystal surface condition and configuration. The defects construction can be regarded as a potential pathway to modulate surface electronic configurations and atomic arrangements, effectively improving the charge-storage efficiency. Hence, it is vital to analyze the crystal defects with the aid of HR-TEM characterizations. As depicted in Fig. 1i, these indexed lattice fringes are not continuous and the corresponding image intensity profiles along the white dashed line of L2 are drawn to compare with L1 (Fig. 1j), indicating the existence of a mass of point defects (marked by yellow arrows). It was also confirmed by XPS characterization, where the point defects should be attributed to Mo-vacancies on account of the breakage of Mo–C–Fe linker on the interface between the *β*-Mo_2_C and metallic Fe. In comparison, the intensity peaks have no obvious depression in profile along the white dashed line of L1, indicative of preserved lattice integrity. As expounded in Fig. 1a, during the thermal treatment process, Fe_3_Mo_3_C specie was formed on the interface of Mo_2_C with Fe phase, which links to Mo_2_C by the Mo–C–Fe linker. Subsequently, the usage of HCl could remove Fe-atoms, Fe-oxide and Fe_3_Mo_3_C by destroying Mo–C–Fe bonds, inducing rich defects at the interface (Fig. S2). The samples containing vacancies are typically accompanied by structural defects, such as lattice dislocation, distortion, and twin crystal, these defect features of which likely originate from the aggregation or cooperative effects of a large number of vacancies, as identified in Figs. S3 and S4 [45, 46].

To further confirm the existence of Mo-vacancies and investigate the variation trend of vacancy concentrations, electron paramagnetic resonance (EPR) spectra are utilized to provide significant information on the paramagnetic centers of materials. In Fig. 1k, the v_0_Fe–Mo_2_C/C barely exhibits any EPR signal, while v_2_Fe–Mo_2_C/C, v_4_Fe–Mo_2_C/C, v_6_Fe–Mo_2_C/C and v_12_Fe–Mo_2_C/C present apparent centrosymmetric EPR peaks at g = 1.9286, which can be ascribed to Mo-vacancies [47, 48]. As is known to all, the peak intensity of EPR can be used to assess the concentration of vacancies in the material [49]. A progressively stronger EPR signal observed from v_2_Fe–Mo_2_C/C to v_12_Fe–Mo_2_C/C samples with increased etching time indicates that acid-etching effectively promotes the formation of Mo-vacancies and regulating the relative ratio of Fe-dopants to Mo-vacancies through this approach is feasible. Taken together, the co-implantation of abundant Mo-vacancies and Fe-atoms in an appropriate proportion, with the Mo–C–Fe linker existing around the Mo-vacancies probably, would efficiently prompt the charge redistribution and equip the v_6_Fe–Mo_2_C/C sample with rich electron-transport shortcuts as well as highly active sites.

**Fig. 2:**
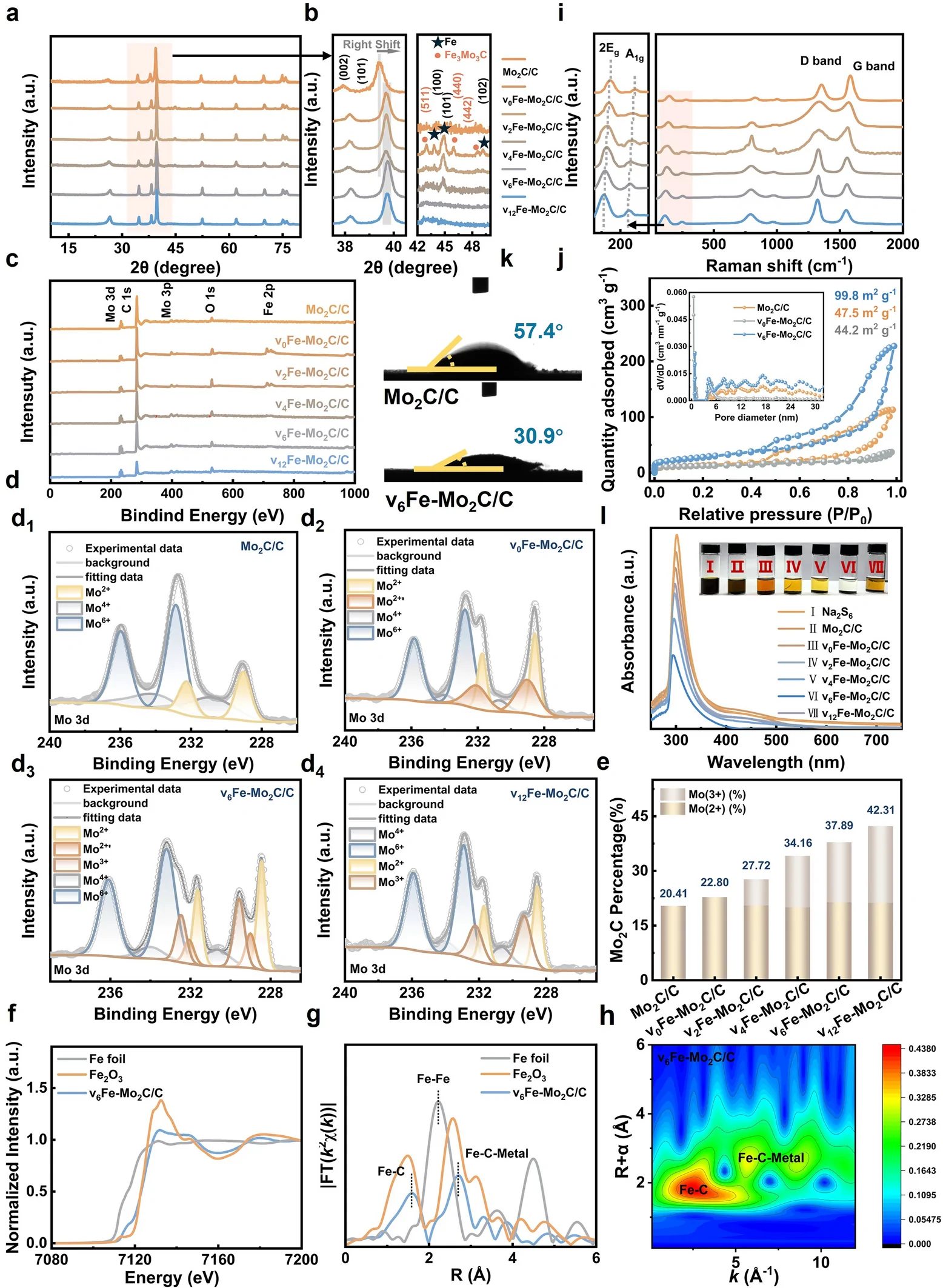
Characterization of v_t_Fe–Mo_2_C/C host materials. **a** XRD plots of full spectra. **b** Related zoom-in regions between 37° (2θ) and 41°, 42° and 50°. **c** Full XPS curves. **d** High-resolution XPS spectra of Mo 3*d* of Mo_2_C/C (**d**_**1**_), v_0_Fe–Mo_2_C/C (**d**_**2**_), v_6_Fe–Mo_2_C/C (**d**_**3**_), and v_12_Fe–Mo_2_C/C (**d**_**4**_). **e** Surface Mo^2+^ and Mo^3+^ percentages of the above samples ascertained by XPS analysis. **f** Fe K-edge XANES spectral of v_6_Fe–Mo₂C/C and reference samples (Fe_2_O_3_ and Fe foil). **g** v_6_Fe–Mo_2_C/C and reference samples (Fe_2_O_3_ and Fe foil) in R-space.** h** Wavelet transforms contour plots for v_6_Fe–Mo_2_C/C. **i** Raman analysis of full spectra and the related zoom-in regions between 50 and 350 cm^−1^. **j** N_2_ adsorption and desorption isotherms, the inset in (**j**) is pore-size distribution curves. **k** Contact angle test. Interaction between NaPSs and various host materials: **l** Ultraviolet–visible spectra of the blank Na_2_S_6_ solution, solutions with a series of host materials after 12 h, the inset in (**l**) is the optical images of the above solutions

The crystal structure and constitution are verified by X-ray diffraction (XRD) results, as presented in Fig. 2a, where the peaks for the Mo_2_C/C and v_t_Fe–Mo_2_C samples can be indexed to the (100), (002), (101), (102), (110), (103), (200), (112), and (201) planes of the standard hexagonal *β*-Mo_2_C structure (PDF number of 65–8766) without additional peaks corresponding to metallic molybdenum, molybdenum oxides or iron-molybdenum alloy, demonstrating the dominant Mo_2_C crystalline phase on the carbon matrix with broadened amorphous carbon peak (≈ 26°). Considering the peaks occurrence of Fe_3_Mo_3_C and metallic Fe, with their gradual attenuation upon extended acid-etching process, the evolutionary pathway of the target product is presented. As described in Fig. 1a, the Fe phase was formed due to the reduction of Fe precursor and the Fe_3_Mo_3_C existed by the interaction between metallic Fe and *β*-Mo_2_C substrate on the interface during the high-temperature carburization process, the acid-etching for which facilitates the atomic-level vacancies construction and doping [50, 51]. Furthermore, Mo_2_C unit cell volume in v_t_Fe–Mo_2_C samples is decreased to the pristine *β*-Mo_2_C counterpart, substantiated by the blue-shift of (101) diffraction peaks because of the smaller Fe-atom than the Mo counterpart, which accords well with the above HR-TEM results of reduced lattice distance toward (101) plane in v_6_Fe–Mo_2_C/C sample (Fig. 2b). Furthermore, inductively coupled plasma atomic emission spectroscopy (ICP) measurement was conducted for the quantitative analysis of Fe contents in various vtFe–Mo_2_C/C samples, as summarized in Table S1. The Fe content shows an apparently decreased trend from 11.30% to 1.37% with the prolonged acid-etching time from 0 to 6 h, and no Fe signal can be detected in the v_12_Fe–Mo_2_C/C sample. Notedly, in the v6Fe–Mo_2_C/C sample, Fe-atoms remain present in the form of Fe_3_Mo_3_C, as confirmed by ICP, XPS and EXAFS analyses (Tables S1, S2 and S5). Combined with the low Fe content revealed by the ICP result and survey spectra of XPS, it can be explained that no distinct Fe_3_Mo_3_C diffraction peaks can be identified in the XRD patterns [52].

X-ray photoelectron spectroscopy (XPS) was conducted to further investigate the surface chemical composition and unsaturated bonds on the point defects after co-implantation strategic design. In the full XPS spectra of the v_t_Fe–Mo_2_C/C samples, the Mo 3*d*, Mo 3*p*, C 1*s*, O 1*s*, and Fe 2*p* peaks can be observed, respectively (Fig. 2c). The surface electronic states toward a series of as-prepared carbides are analyzed by the high-resolution Mo 3*d* spectra (Figs. 2d and S5), where the Mo 3*d* curves of v_6_Fe–Mo_2_C/C can be deconvoluted into five doublet peaks. Thereinto, the Mo^2+^ assigned to Mo–C bonding (*β*-Mo_2_C), Mo^2+'^ and Mo^3+^ indexed to Mo–C bonding of Fe_3_Mo_3_C and Mo-vacancies, respectively, could be deemed as the active sites for the electrocatalytic energy storage based on the redox reactions [53–55]. And the higher oxidation states (Mo^4+^ and Mo^6+^) can be ascribed to the unavoidable surface oxidation when these materials are exposed to air. First-principles calculations indicate that the introduction of metal vacancies modulates the surface electronic structure and changes the charge-density distribution, which in turn can influence the local chemical states. Specifically, Mo-vacancy formation breaks the inherent equilibrium and redistributes the electron localization of Mo_2_C, where the departure of the Mo atom will take away the electrons that were originally intended to transfer to surrounding nonmetal atoms. As a consequence of Mo removal, the electron density in the vicinity of Mo-vacancies tends to be depleted to compensate for the electron deficiency of nonmetal atoms, ultimately rendering the Mo hollow a large positive state in Mo_2_C [47, 50, 56–59]. Inspiringly, the Mo-C content sum of Mo^2+^ and Mo^3+^ in molybdenum carbide is drastically boosted after Fe-doping than that of pristine Mo_2_C, thereby it can be rationally speculated that the introduction of Fe-atoms facilitates the formation of Mo-based carbide and enables more active sites for charge storage (Fig. 2e). In all surface Mo species, the concentration of Mo^2+'^ species is gradually decreased from 22.77% across v_0_Fe–Mo_2_C/C to 0% across v_12_Fe–Mo_2_C/C. On the contrary, the percentage of Mo-vacancies (Mo^3+^) is apparently increased from 0% across v_0_Fe–Mo_2_C/C to 21.10% across v_12_Fe–Mo_2_C/C. As testified by the quantitative analysis (Table S2), Fe-atom doping, together with the in-situ Mo-vacancies formation caused by the chemical etching, has been successfully achieved in v_2_Fe–Mo_2_C/C, v_4_Fe–Mo_2_C/C and v_6_Fe–Mo_2_C/C samples, and the dual-modification strategy exert a direct influence on the surface electronic states. The high proportion of Mo-vacancies aligns well with the ample point defects of the v_6_Fe–Mo_2_C/C sample shown in the FE-TEM results (Fig. 1i).

Moreover, the Fe 2*p* XPS spectra show the peak positions, the related Fe-bonding environments and percentages in Fig. S7 and Table S3. In addition to the Fe metal (~ 707.47 eV) and Fe^2+^ (~ 710.92 eV), the peak positions of Fe 2*p*_*1/*2_ in the range of 713.61–713.94 eV originate from the Mo–C–Fe bonding (Fe_3_Mo_3_C) [50, 60, 61]. Notably, there is only Fe_3_Mo_3_C constitution in the v_6_Fe–Mo_2_C/C material and no signals related to metallic Fe, Fe-oxide, as well as even no Fe_3_Mo_3_C is detected in the v_12_Fe–Mo_2_C/C sample, as listed in Table S2 in detail. As far as the detailed binding energy data are concerned (Table S2), it is clearly identified that the Mo^2+^ peak in Mo 3*d* spectra of v_0_Fe–Mo_2_C shifts toward lower binding energy direction, implying that Mo atom is at electron-enrich state compared with that of pristine Mo_2_C, which can be attributed to the higher electronegativity of Mo (2.16) versus Fe (1.83), thereby inducing the electron transfer from Fe to Mo and the peaks red-shift [62]. Furthermore, the Mo^2+^ peak position in Mo 3*d* spectra of v_12_Fe–Mo_2_C without Fe species is also localized at a lower binding energy in contrast to that of pristine Mo_2_C. Correspondingly, the peak position related to Mo–C bond in C 1*s* spectrum of v_12_Fe–Mo_2_C (284.39 eV) shifts to a higher binding energy than that of *β*-Mo_2_C (284.20 eV) without any treatment (Fig. S8). These phenomena indicate that Mo-vacancies play an important role in charge redistribution at their surrounding atoms, which urges the electrons to flow from C atom to the adjoining Mo atom even though the ability to grab electrons of C is stronger than that of Mo. Therefore, both the surface Mo-vacancies and Fe-doping could effectually promote charge transfer and regulate the electronic state distribution on the surface, which might lead to the conductive properties change in the local domain of the semiconductor, indicating the potential of constructing the homojunction in this individual Mo_2_C material. Overall, with the continuous acid treatment, the Fe content exhibits a pronounced decreasing trend, accompanied by the gradual disappearance of Fe metal and Fe^2+^ species. Concurrently, the proportion of surface Mo-vacancies increases markedly, which is primarily attributed to the aggravated cleavage for the Mo–C–Fe bonds, this behavior of which is fully consistent with the evolution process toward v_t_–Fe–Mo_2_C/C discussed in Fig. 1a. Ultimately, the relative contents of Fe species and surface Mo-vacancies (R value) are flexibly regulated, thereby obviously optimizing the surface electronic properties through the formation and enrichment of homojunction interface over the whole host material, which is reasonably expected to play a critical role in providing favorable ligand environments for capturing active sulfur-based species and contributing to the enhanced catalytic performance (Table S4).

X-ray absorption near-edge structure (XANES) and extended X-ray absorption fine structure (EXAFS) are employed to further analyze the coordination environment and bonding structure. As shown in Fig. 2f, Fe absorption edge suggests that the Fe species in v_6_Fe–Mo_2_C/C is in a high state, which is similar to that of the Fe_2_O_3_ reference [60]. Therefore, the valence state of Fe in v_6_Fe–Mo_2_C/C can be determined to be + 3, which accords well with the Fe_3_Mo_3_C phase presented in XPS results. More in-depth analysis of the radial distribution function (R-space) via Fourier transform reveals the peak at ≈ 1.58 Å, corresponding to the characteristic Fe–C bond peak. Moreover, compared to Fe foil, the Fe component in v_6_Fe–Mo_2_C/C sample shows a strong peak at ≈ 2.69 Å, indicating the presence of Fe–C-Metal bonds (Fig. 2g) [63–68]. And these peak positions are very close to those corresponding to the Mo–C and Mo–C–Mo bonds revealed in the Mo_2_C R-space analysis of Mo_2_C–1Ni/SiO_2_ sample [69]. The above results testify that Fe-atoms are still present in the form of carbide, which is consistent with the XPS analysis. Meanwhile, FT-EXAFS curve fitting analysis is conducted to obtain quantitative coordination information and give the R-space and k-space plots (Fig. S9 and Table S5). Particularly, wavelet transform results ulteriorly provide the evidence that v_6_Fe–Mo_2_C/C are primarily characterized by Fe–C coordination in the first coordination shell and Fe–C-Metal coordination in the second coordination shell (Fig. 2h).

In order to investigate the crystal structure and concomitant electron coupling effects induced by the synergy of metal-atom doping and cation vacancies, Raman spectroscopy was performed. As shown in Fig. 2i, the Raman vibrational peaks near 146, 278, 826, and 999 cm^−1^ of as-prepared materials originate from *β*-Mo_2_C, and the two noticeable characterization peaks appear at 1352 and 1583 cm^−1^, corresponding to the defect (D) and graphitic (G) carbon, respectively. It manifests high purity and quality toward a series of Mo_2_C-based carbon composites. It should be noted that a gradual negative shift is observed in the Raman peaks of v_t_Fe–Mo_2_C/C with the enhancement of HCl etching degree for the Fe-doped molybdenum carbide materials, indicating the constantly increased crystal structural defects, which can be further ascribed to the obviously changed electron distribution induced by the more and more Mo-vacancies [45, 70, 71]. The analysis result is perfectly consistent with the electron transfer process learned in XPS characterization. With the help of the carbon substrate owning the higher defect level, the ample active sites with high charge density would be supplied for the competitive v_6_Fe–Mo_2_C/C@S cathode. Besides, the specific Brunauer–Emmett–Teller surface area (S_BET_) of v_t_Fe–Mo_2_C/C presents a monotonic escalation trend with the extension of etching duration from 0 to 6 h, concomitant with the structural porosity evolution and increase of larger pore architecture, which can be explained by the gradual Fe-based species dissolutions with the increase of vacancy amounts, as displayed in Fig. S10 [72, 73]. Notably, the v_6_Fe–Mo_2_C/C sample shows the highest S_BET_, with a broad distribution of narrow micropores mainly located at ≈ 0.85 nm and a few small mesopores in 3.5–31 nm size, which would afford more exposed active sites and low-resistance ion channels (Fig. 2j). Meanwhile, the co-implantation strategy based on Fe-doping and in-situ generated Mo-vacancies also noticeably improves the surface wettability (Fig. 2k). In the high-resolution S 2*p* spectrum (Fig. S11), two apparent splitting peaks at 164.0 and 165.3 eV are related to the S 2*p*_*3/2*_ and S 2*p*_*1/2*_ in elemental S, respectively [53]. EDS mapping also confirms the successful loading and uniform distribution of active sulfur (Fig. S12). The thermogravimetric analysis (TGA) curve of the v_6_Fe–Mo_2_C/C@S, as exhibited in Fig. S13, indicates the sulfur content about 48.8 wt%. In visualized adsorption experiments (Fig. 2l), v_6_Fe–Mo_2_C/C makes the solution color from dark yellow to approximately colorless, with the maximal decrease in absorbance of S_6_^2−^ at ≈ 294 nm. In short, these results prove the most robust adsorption ability of the v_6_Fe–Mo_2_C/C composite for polysulfides, which can be ascribed to the high-activity sites due to charge redistribution via the induced-homojunction. Finally, the diffusion of NaPSs is efficiently inhibited to enhance the reversibility and cycling stability.

### Mechanism Analysis of Interface Formation and Interaction

**Fig. 3:**
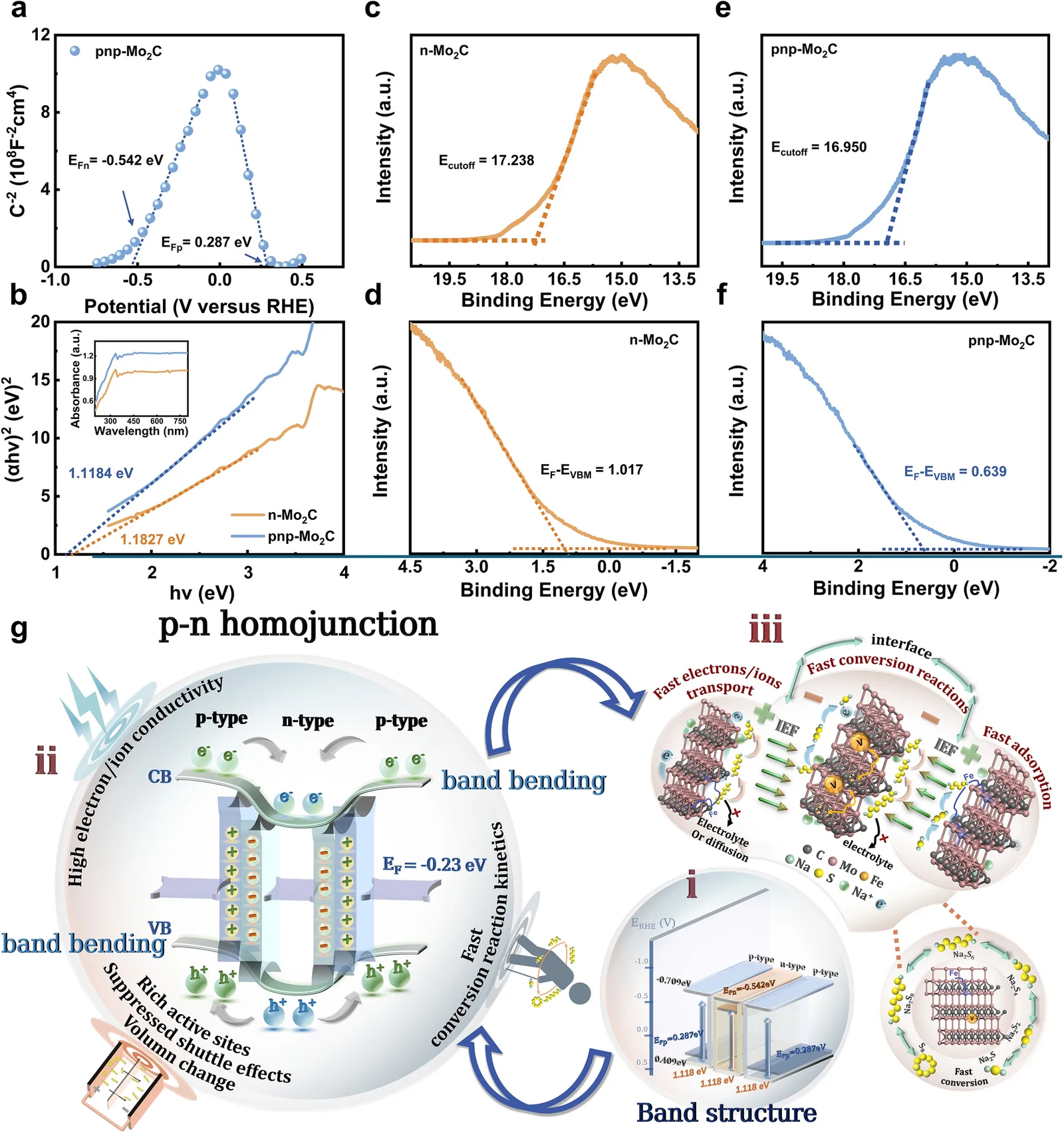
Mechanism analysis of interface formation and electrochemical reactions dynamics improvement. **a** Mott-Schottky plot of pnp-Mo_2_C. **b** Tauc plots of absorption spectra of n-Mo_2_C and pnp-Mo_2_C. **c, d** UPS spectra of n-Mo_2_C. **e, f** UPS spectra of pnp-Mo_2_C. **g** Schematic of the high-activity interface with bidirectional IEF: (**i)** Individual E_F_ positions of p-type and n-type areas in pnp-Mo_2_C as well as final E_CB_/E_VB_ (conduction band and valence band) of pnp-Mo_2_C. (**ii)** Sketch for electron–hole transfer and then established IEF on the interface in pnp-Mo_2_C. (**iii)** Visual description with theoretical models for the formed charged space on the interface and the energy storage process involved in the adsorption and “solid–liquid-solid” conversion of NaPSs. The “V” represents Mo-vacancy and the represented color of Fe-atom is purple

The charge density decides the interaction between hosts and polysulfide intermediates, which serves as the first step for the subsequent electrocatalytic process. And the charge transport capability also governs the conversion reaction rate, thus the relative investigation of which is very essential. The analytic approaches involving the homojunction have been extensively reported in the literature, which provides the effective and reliable reference for our exploration. Firstly, the Mott-Schottky (M-S) tests were conducted to analyze the conductivity type of the Mo_2_C only decorated by Mo-vacancy as well as Mo_2_C synergistically modified by Fe-doping and Mo-vacancy. With regard to the Mo_2_C with cation vacancies, the slope of M-S curve is ascertained to be positive, denoted as n-Mo_2_C (Fig. S14). Intriguingly, the M-S plot of the other sample manifests straight lines with positive and negative slopes at different potential ranges, implying the co-existence of n-type and p-type conductivity features in this material, which is most plausibly attributed to the respective modulations for the surrounding electronic structures induced by Mo-vacancy or Fe-doping (Fig. 3a). Within the individual material, the intimate contact and alternating stacks of n-type and p-type conductive domains can be determined as the sandwiched p-n homojunctions. Thus, it is denoted as pnp-Mo_2_C. Notably, the Fermi level (E_F_) of pnp-Mo_2_C is determined by extrapolation of the abscissa intercept, where the Fermi levels of n-type and p-type domains are signed as E_Fn_ (− 0.542 eV vs. Reversible Hydrogen Electrode (RHE)) and E_Fp_ (0.287 eV vs. RHE), respectively. In view of the E_Fn_ toward n-Mo_2_C approaching that of pnp-Mo_2_C and previous relevant reports, it can be deduced that the E_Fn_ of pure n-type Mo_2_C can stand for that of homojunction pnp-Mo_2_C to a certain degree [74–76]. Moreover, ultraviolet–visible diffuse reflectance spectroscopy (DRS) was used to monitor the absorption properties and band gap (E_g_) of pnp-Mo_2_C and n-Mo_2_C. Given the further handled Tauc plots of Fig. 3b, pnp-Mo_2_C has a narrower intrinsic E_g_ of 1.118 eV than that of n-Mo_2_C (1.183 eV), implying the enhanced electrical conductivity. Generally, the work functions are ascertained by subtracting the cutoff energy (E_cutoff_) of the secondary electrons from the He I excitation energy (21.22 eV), where the E_cutoff_ values of n-Mo_2_C and pnp-Mo_2_C are obtained by the ultraviolet photoelectron spectrum (UPS) measurement (Fig. 3c–f), thus the E_F_ of n-Mo_2_C and the pnp-Mo_2_C can be further determined as − 0.521 eV (vs. RHE) and − 0.230 eV (vs. RHE) [77]. Thereinto, the energy versus RHE (E_RHE_) is converted through the energy versus vacuum (E_Vac_) with the help of the equation: E_Vac_ + E_RHE_ = − 4.5 eV, and as anticipated, these E_Fn_ values exhibit good agreement with that attained by the electrochemical measurements [78]. Notably, the E_F_ of pnp-Mo_2_C resides between those values of n-type and p-type semiconductors, thereby validating the rationality of aforementioned analytical protocol. According to the onset valence band photoemission on the low binding energy edge of the UPS spectrum, the positions of the valence band maximum (E_VBM_) with respect to the Fermi level are confirmed to be 0.499 eV (n-Mo_2_C) and 0.409 eV (pnp-Mo_2_C).

The detailed band positions for pnp-Mo_2_C are depicted in “region i” of Fig. 3g and listed in Table S6, in which the individual E_F_ locations of n-Mo_2_C and p-Mo_2_C are also labeled to determine the interfacial interactions after the contact of n-Mo_2_C and p-Mo_2_C [79–81]. It can be attributed to the implantation of Fe and vacancy, respectively, induce the material to transform Mo_2_C into semiconductors with different conductivity types. Particularly, the Mo_2_C after co-implantation design enables the conduction band position (E_CB_) variation compared with n-Mo_2_C, integrating with the reduced E_g_, which can excite more electrons and promote their fast migration, implying the enhanced conductivity and redox electrocatalytic activity. The synergistic feature of n-type and p-type semiconductors brings about the sandwiched p-n homojunction formation in the form of three-unit cells. The unique structure contributes to shortening the diffusion distance of charge and offering ample high-activity surface for polysulfides adsorption. More significantly, the homojunction caused by unequal energy levels in the same materials facilitates the highly effective carrier separation and migration on the interface between the Fe-doped Mo_2_C region and the vacancy-modified Mo_2_C region. Based on the above analyses for the band structure, as represented by the relative positions of E_F_, the carriers migration direction is ascertained and depicted in “region ii” of Fig. 3g, leading to the positive and negative charges accumulation in the p-type and n-type conductive regions, respectively, which implies the establishment of the internal electric field (IEF) directed from the p-type region toward the n-type region. Noteworthy, the spontaneous charge transport process accompanied by the apparent band bending phenomenon is also intuitively illustrated. In other words, the band bending process represents the process of the homojunction formation, implying the construction process of the IEF. As listed in Table S6, the results obtained from UPS and M-S measurements provide direct evidence for the energy level variation. The E_F_ of pnp-Mo_2_C homojunction (− 0.230 eV vs. RHE) differs from those of n-Mo_2_C only with Mo-vacancies (− 0.521 eV vs. RHE) and Fe-doped Mo_2_C (0.287 eV vs. RHE)), where E_F_ of pnp-Mo_2_C lies between E_F_ of the latter two. And the valence band location (E_VB_) and conductive band location (E_CB_) of pnp-Mo_2_C can be determined at 0.409 and − 0.709 eV, respectively. For n-Mo₂C, E_VB_ and E_CB_ are at 0.499 and −0.684 eV, respectively. The above data indicate that, at the interface of adjacent Fe-doped Mo_2_C structure and the Mo-vacancy local structure, the directional migration of interfacial carriers promotes band bending and E_F_ shift, ultimately leading to their equilibration within the formed homojunction. Moreover, the theoretical models of Mo_2_C with Fe-atom doping or Mo-vacancy are used for making up three units to vividly express the two-way IEF on the homojunction interface in “region iii” due to the distinct conductive types in the individual Mo_2_C regulated by Fe and vacancies at the atomic-level, respectively. This integrated analytical strategy combining Mott-Schottky, UV and UPS measurements has been widely adopted in studies of homojunctions, which collectively demonstrate the sufficiency and reliability of evidence provided by this series of methods above. In virtue of the effects of “induced-homojunction”, the directional charge migration leads to localized charge enrichment at active sites of pnp-Mo_2_C, which amplifies their chemical etching capability toward NaPSs and promotes the strong bonding of Mo-S, thereby further weakening the S–S bonds and remarkably facilitating the decomposition of NaPSs to engage in the subsequent reduction pathways. Notedly, the suppression of shuttling effects is achieved not only through simple immobilization, more importantly, via the rapid conversion of soluble long-chain NaPSs to decrease the possibility of dissolution, which tries their best to reduce the loss of active materials. In another case, bidirectional IEF can be regarded as a powerful driving force for rapid ion transport and electron conduction to timely reach the above-mentioned highly active sites and proceed with the full conversion reactions, which in turn extremely accelerates the redox dynamics of the active sulfur matrix and makes the fast-charging ability possible.

### Electrochemical Performance

**Fig. 4:**
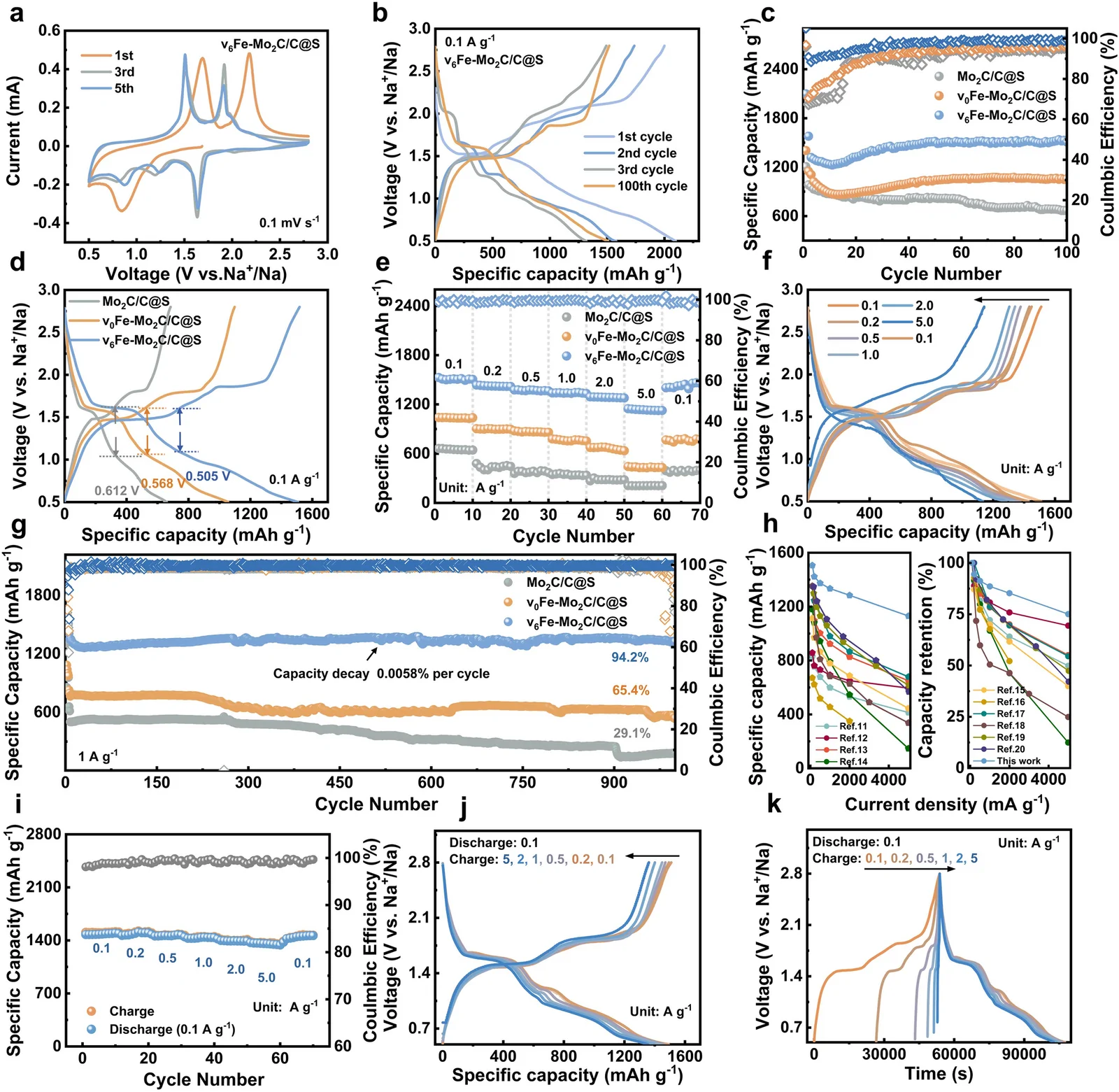
Electrochemical performance and fast-charge testing. **a** CV curves at different cycle numbers. **b** GCD profiles in the initial three cycles and 100th cycle of v_6_Fe–Mo_2_C/C@S cathode. **c** Cycling performance at 0.1 A g^−1^. **d** GCD profiles at 100th cycle. **e** Rate performance. **f** GCD profiles at various current densities of v_6_Fe–Mo_2_C/C@S cathode. **g** Long-term cycling performance at 1 A g^−1^. **h** Rate performance comparison of the cathode reported in this work with the state-of-the-art cathodes of Na||S batteries published recently. **i-k** The fast-charging rate performance of Na–S battery with v_6_Fe–Mo_2_C/C@S cathode: discharging at 0.1 A g^−1^

To overall explore the homojunction impacts manipulated by energy band bending due to adjacent atom-level modification, a series of v_t_Fe–Mo_2_C/C@S cathodes were assembled into RT Na-S cells and the relative electrochemical performance was tested. In the cyclic voltammetry (CV) curves at 0.1 mV s^−1^, it can be discovered that a tiny peak occurs at 2.02 V and several apparent reduction peaks, corresponding to the multi-step conversion of sulfur (S_8_) to long-chain polysulfides (Na_2_S_n_, 4 < n ≤ 8), the formation of Na_2_S_4_, the further conversion to short-chain sodium polysulfides (Na_2_S_2_/Na_2_S) and solid-state interface (SEI) layer formation, respectively. Likewise, the charge process involves two peaks around 1.51 and 1.92 V, indexed to the reversible oxidation behavior [82–84]. Of note, the almost coincident CV curves after the first cycle suggest good stability and reversibility of the Fe–Mo_2_C-6/C@S cathode (Fig. 4a). As demonstrated in Fig. S15, the v_6_Fe–Mo_2_C/C@S electrode delivers the earliest cathodic peaks and anodic peaks, serving as direct evidence for the anticipated rapid reduction reactions and oxidative regeneration of long-chain NaPSs. Meanwhile, the v_6_Fe–Mo_2_C/C@S electrode shows noticeably higher peak currents accompanied by larger CV integral areas than those of Mo_2_C/C@S and v_0_Fe–Mo_2_C/C@S cathodes, further demonstrating the favorable kinetics characteristic during the redox process.

As anticipated, the galvanostatic reaction plateaus of the v_6_Fe–Mo_2_C/C@S electrode match well the peaks in the CV curves. Specifically, the v_6_Fe–Mo_2_C/C@S cathode delivers the highest initial specific capacity of 2099 mAh g^−1^ at the current density of 0.1 A g^−1^ for the first cycle and can remain the highest reversible capacity of 1508 mAh g^−1^ with CE of 99.3% over 100 cycles than that of Mo_2_C/C@S (669.1 mAh g^−1^), v_0_Fe–Mo_2_C/C@S (1056.5 mAh g^−1^) and other v_t_Fe–Mo_2_C/C@S (t = 2, 4 or 12) cathodes, suggesting a superior utilization for active material in virtue of the moderate affinity to the NaPSs and effective catalytic capability of v_6_Fe–Mo_2_C/C in the cathode (Figs. 4b, c and S18a, b). Correspondingly, the trapping capability of various host materials for the Na polysulfides has also been verified by the adsorption experiments related to the Na_2_S_6_ solution. As aforementioned in Fig. 2l, the color of Na_2_S_6_ solution after adding the v_6_Fe–Mo_2_C/C materials presents almost colorless with the largest decrease in absorbance of S_6_^2−^, while the homologous solutions with Mo_2_C/C and v_0_Fe–Mo_2_C/C only have a slight change after adsorption for 12 h. Particularly, the formation of solid electrolyte interphase (SEI) and cathode electrolyte interphase (CEI) on the surface of electrodes can be attributed to the decomposition of carbonate electrolyte and the nucleophilic attacks from nascent polysulfides on the solvent molecules during the activation process, which leads to the initial irreversible discharge capacity and the different first-cycle CV curve with distinct redox behavior [10, 18, 85, 86]. Furthermore, the discharge plateaus of the subsequent loops are below 2.0 V, suggesting the main formation of short-chain NaPSs and circumvention of continuous electrolyte decomposition due to the formation of CEI, contributing to the long-term utilization of active species and the cycling stability [5, 87]. And the apparently boosted initial CE of v_6_Fe–Mo_2_C/C@S cathode than other counterparts demonstrates the evidently enhanced Na_2_S conversion dynamics, which constantly increases over 99.0% and maintains stable in the subsequent charge-storage process. Generally, the ratio of Q_2_/Q_1_ can be associated with the electrocatalytic ability of host materials for NaPSs conversion, where the capacity fading during the Q_2_ stage reflects the sluggish kinetics process and the shuttling effects caused by the soluble NaPSs. Thereinto, the capacities of Q_1_ and Q_2_ are indexed to the reduction processes of soluble NaPSs and the subsequent conversion to nucleated Na_2_S_2_/Na_2_S, respectively. Hence, the highest Q_2_/Q_1_ ratio of v_6_Fe–Mo_2_C/C@S cathode further substantiates its best catalytic activity toward the NaPSs transformation and the most efficient sulfur utilization (Fig. S16) [82, 88]. It is clearly reflected that the Mo-vacancy and Fe-doping heterogeneously modulate the band structure to trigger the directional charge migration and induce charge accumulation at the active sites, accompanied by the interfacial electric field formation providing the additional driving force for accelerating the electrons/ions transport. Collectively, the capture for soluble NaPSs, the effective cleavage of S–S bonds and succedent sodiation are noticeably promoted to enhance the discharge depth and conversion ability to the final product Na_2_S. Inspiringly, during the repeated charge–discharge processes, the specific capacity of the v_6_Fe–Mo_2_C/C@S shows an initial decline followed by a subsequent increase trend, which we assume to be on account of the gradual activation process in the cathode to form rapid charge transport channels or postponed self-conditioning of the battery system. Thereafter, the cell assembled by v_6_Fe–Mo_2_C/C@S cathode exhibits significantly stable discharge capacity, eventually maintaining 1508 mAh g^−1^ at the 100th cycle, as shown in Fig. 4b. Notably, the negligible capacity of pure v_6_Fe–Mo_2_C/C electrode without sulfur clearly confirms that sulfur is the sole active material (Fig. S17), and the excellent performance obtained in this work originates from the improvement of sulfur-based energy storage process by the host rather than from the introduction of additional capacity-contributing materials. Figure S18b expounds that the stepwise activation process is a common phenomenon for all S cathodes with v_t_Fe–Mo_2_C/C hosts, but the overall performance of the v_6_Fe–Mo_2_C/C@S is undoubtedly the best. As a sharp contrast, the capacity degradation rate of v_0_Fe–Mo_2_C/C@S cathode is much faster and the energy storage ability toward Mo_2_C/C@S cathode presents unremitting and severe deterioration to remain only 68.07% corresponding to the initial capacity value, suggesting that the joint implantation of Mo-vacancies and Fe-atom endows the v_6_Fe–Mo_2_C/C@S with a robust competitive advantage in the cycling-stability aspect through a unique n-p electronic transmission effects on the charge distribution on each redox-active sites and charge conduction. Noteworthy, the v_6_Fe–Mo_2_C/C@S cathode undergoes the smallest polarization in terms of discharge and charge profiles, in conjunction with the largest storage capacity, the performance improvement of which originates from the valid inhibition for dissolved NaPSs shuttling and stabilization for the Na-metal anode. It can be further explained by the enhanced affinity for polysulfides and decreased conversion barrier due to the induced-homojunction (Fig. 4d).

Considering the separators are in close contact with the cathodes, an analysis of separators after cycling can be regarded as an indicator of NaPSs shuttling, which was carried out by disassembling from batteries with Mo_2_C/C@S, v_0_Fe–Mo_2_C/C@S and v_6_Fe–Mo_2_C/C@S cathodes, respectively. Typically, the dissolution of polysulfides into the electrolyte and their subsequent shuttling between the cathode and anode result in noticeable discoloration of the separators. As can be observed in Fig. S19, in contrast to the most severely yellowing separator corresponding to the Mo_2_C/C@S cathode, the separator from the v_6_Fe–Mo_2_C/C@S system remains nearly unchanged in color. It implies a negligible loss of polysulfides toward the anode side and provides strong evidence for the efficient constraint and conversion by v_6_Fe–Mo_2_C/C host to obviously inhibit long-chain polysulfides shuttling, which is crucial for the retention of active materials.

To further demonstrate the structural and compositional stability of the v_6_Fe–Mo_2_C/C material after 100 cycles, the post-mortem analyses of XPS, SEM and TEM were performed. Noteworthy, the morphology of cycled v_6_Fe–Mo_2_C/C catalyst is well preserved to promise the fully exposed active sites and ion transport pathways (Fig. S20a, b). High-resolution TEM (HR-TEM) and the corresponding Fast Fourier transform (FFT) image reveal a distinct lattice spacing of 0.218 nm assigned to the (101) crystal plane of hexagonal Mo_2_C, where a reduced lattice distance than pristine Mo_2_C (0.228 nm) can be explained by the insertion of Fe-atoms with a smaller size. In conjunction with a large number of point defects (marked by yellow arrows) that originate from the Mo-vacancies, the dual-implantation modification is well maintained (Fig. S20c, d). With regard to the analysis of compositional stability, Fig. S21a presents the Mo 3*d* spectrum of the v_6_Fe–Mo_2_C/C@S cathode at the fully charged state through cycling. Notably, the characteristic peaks corresponding to Mo^2+^, Mo^2+^′, and Mo^3+^ species coexist, and their relative ratio of Mo^2+^′ and Mo^3+^ species assigned to Fe_3_Mo_3_C and Mo-vacancies, respectively, remains consistent with those in the pristine state. Meanwhile, no noticeable change in the peak positions is observed. Moreover, the Fe 2*p* spectrum recorded in Fig. S21b after cycling exhibits exclusively Fe^3+^ characteristic peak representing the Fe_3_Mo_3_C, where the behavior is highly identical to that of the acid-etched initial state of v_6_Fe–Mo_2_C/C, suggesting the sufficient chemical stability throughout the reaction process. Typically, the unfilled d orbitals of oxidized Mo can attract electrons from NaPS anions, thereby facilitating their conversion, where the electron transfer process leads to the peaks downshift relative to the pristine sample in Mo 3*d* spectrum. During the subsequent charging process, the Mo peaks generally upshift back toward their original states. Such reversible peak position evolution has been repeatedly reported in previous studies [89–92]. Therefore, the absence of noticeable peak shifts at the fully charged state compared with the pristine state provides robust evidence for the chemical stability of the v_6_Fe–Mo_2_C/C catalyst in the repeated charge–discharge cycles [93, 94]. More importantly, the maintained coexistence status of Fe-doping and Mo-vacancy structures in the optimized ratio, as depicted in Fig. S21a, facilitates the construction of abundant homojunction interfaces. And these interfaces afford highly active sites for reactive sulfur species and the intrinsic electric fields, ensuring effective trapping of NaPSs and promoting the breakage of S–S bonds while enabling fast electron and ion transport to accelerate NaPSs conversion. These functions ultimately promise sustained high utilization of active sulfur, while preventing active materials loss and capacity fading, exhibiting excellent electrochemical stability. Collectively, the catalyst retains both structural integrity and chemical-state stability after prolonged cycling, which explains its robust electrochemical cycling performance with a tiny attenuation rate of 0.0058% per cycle at a current density of 1 A g^−1^. Notably, the chemical environment and the homojunction architecture are well preserved even after cycling, allowing the highly active interfaces to continuously enable strong NaPSs trapping and efficient NaPSs conversion for highly efficient sulfur utilization. Meanwhile, the intact morphology and stable framework ensure sustained exposure of active sites, fast mass transport, and efficient electron conduction through the carbon matrix, while alleviating the volume variation of active sulfur to some extent. Consequently, these features guarantee a long-term stable Na–S battery system with fast-charge-storage kinetics.

To reveal the electrocatalytic capability and demonstrate the effectiveness of “induced-homojunction” concept in accelerating the transformation reactions kinetics aspect to render v_6_Fe–Mo_2_C/C acting as an ideal host for Na–S batteries, the rate performance at various current densities from 0.1 to 5 A g^−1^ was tested, as drawn in Fig. 4e. To obtain the realistic capability, the measurement was conducted after 100 cycles at 0.1 A g^−1^. As the current densities increase, the highest reversible capacity and slowest degradation velocity toward v_6_Fe–Mo_2_C/C@S of 1505, 1425, 1371, 1337, 1288, and 1137 mAh g^−1^ at 0.1, 0.2, 0.5, 1, 2, and 5 A g^−1^, respectively, are presented to deliver the unparalleled rate properties than that of Mo_2_C/C@S (656, 440, 368, 347, 283, and 209 mAh g^−1^ under a series of currents above, respectively) and that of v_0_Fe–Mo_2_C/C@S cathodes (1036, 905, 869, 761, 680, and 429 mAh g^−1^ under above currents, respectively). And the v_6_Fe–Mo_2_C/C@S cathode also shows the best capacity recovery when the current density reverts back to 0.1 A g^−1^. It can be rationally supposed that much better rate capability and reversibility mainly benefit from the quick breakage of S–S bonds in reaction specimens and the high-speed mobility of electrons/ions to carry out full and timely sodiation with NaPSs at various orders, which could be further attributed to the introduction of the sandwiched p-n homojunction structure induced by the co-implantation design. In specific, Fe-doping and Mo-vacancy, respectively, enable Mo_2_C to become semiconductors with diverse conductive types, such as p- or n-type, via the intimate contact in individual Mo_2_C material, leading to the formation of rich homojunctions with interfacial p-n effects. In view of the composition identity on both sides of the interface, the continuous band bending takes place, beneficial to carrier separation and fast transport, implying the charge accumulation on sites, robust entrapment ability and spatial potential gradient establishment. Hence, the electrons/ions conductivity and the electrocatalytic capability of Mo_2_C are extremely optimized to highlight the redox kinetics superiority. As collected charge/discharge curves in Fig. 4f, the reaction plateaus are distinguishable and nearly unchanged even at high current densities, integrating the slightly aggravated voltage polarization, to collectively confirm the outstanding Na-ions transport behaviors and fast reaction kinetics. The high mass loading cathode was examined to evaluate the practical applicability of v_6_Fe–Mo_2_C/C@S. Despite a challenging task of sulfur loading up to 4.1 mg cm^−2^ (E/S = 22.0 μL mg^−1^), the electrode still maintains a specific capacity of 1072.8 mAh g^−1^ throughout 100 cycles at 0.2 A g^−1^ (Fig. S22a). The well-defined voltage plateaus observed in the charge–discharge profiles, as shown in Fig. S22b, together with the stable cycling behavior, indicate the fast sulfur-redox kinetics and the robustness of the v_6_Fe–Mo_2_C/C@S cathode material. In addition, the electrochemical performance of v_6_Fe–Mo_2_C/C@S cathode displays the enormous competitiveness compared to the previously reported RT Na–S batteries, as listed in Table S7. Moreover, the almost top-tier rate capability is highlighted in Fig. 4h, together with the above advantages including the energy density aspect, endowing the v_6_Fe–Mo_2_C/C@S cathode with the enormous competitiveness in the practical applications [53, 95–104].

In addition, the long-term cycling stability of Na–S batteries with various cathodes is assessed at 1 A g^−1^. After 5 loops at a low current density of 0.1 A g^−1^ and the initial SEI formation, the capacity presented a reduced trend of v_6_Fe–Mo_2_C/C@S cathode in the first 30 cycles and then slowly increased to 1361 mAh g^−1^ during the stepwise activation process in the following cycles. Inspiringly, the energy storage steadily proceeded and the capacity was up to 1327 mAh g^−1^ with tiny decay even after 1000 cycles, which is equal to 0.0058% attenuation rate per cycle (Fig. 4g). And the approximate curves throughout the cycling duration demonstrate the stable reaction interface and weak polarization of the v_6_Fe–Mo_2_C/C@S sample, as shown in Fig. S23. In accordance, the Coulombic efficiencies become higher than 99% and remain stable after the activation process to prove the excellent reversibility during the charge and discharge process. Nonetheless, the Na||S battery with v_0_Fe–Mo_2_C/C host shows evident capacity decay and retains only 65.4% of the initial value after 1000 cycles, let alone the sharp degradation with 29.1% capacity in the work procedure toward Mo_2_C/C@S cathode, the ceaselessly deteriorating procedure in which should be imputed to the dissolution of long-chain NaPSs and diffusion to react with the anode, high-resistance ions migration pathways as well as the poor electron conductivity, leading to the severe active species loss and high redox barrier. When an appropriate relative ratio of Fe-doping to Mo-vacancies is achieved, the abundance-degree of homojunction interface reaches its maximum and the formed IEF is distributed in the most extensive range across the Mo_2_C, thereby describing it as the large‑range internal electric field. The above electrochemical tests have confirmed that v_6_Fe–Mo_2_C/C sample corresponds to the system with the highest abundance-degree of homojunction interfaces and accelerates the reaction kinetics to the greatest extent.

Based on the ICP and EPR measurements and quantitative XPS analysis, the samples can be divided into several types: (i) v_0_Fe–Mo_2_C/C represents the sample only with Fe-doping; (ii) v_2_Fe–Mo_2_C/C, v_4_Fe–Mo_2_C/C and v_6_Fe–Mo_2_C/C represent the sample with Fe-doping and Mo-vacancies simultaneously, and the differences among the three samples lie in the different ratio of the Fe-dopant to Mo-vacancies; (iii) v_12_Fe–Mo_2_C/C represents the sample only with Mo-vacancies. The electrochemical performance of v_0_Fe–Mo_2_C/C@S, v_12_Fe–Mo_2_C/C@S and v_6_Fe–Mo_2_C/C@S samples is further compared to elucidate the individual roles of Fe-doping or Mo-vacancies, and the role of homojunction structure based on the synergistic effects in performance improvement. As shown in Figs. 4c and S18b, the v_6_Fe–Mo_2_C/C@S cathode maintains an exceptional capacity of 1508.0 mAh g^−1^ after 100 cycles at 0.1 A g^−1^, whereas v_12_Fe–Mo_2_C/C@S delivers 1279.9 mAh g^−1^ under the same conditions. In contrast, v_0_Fe–Mo_2_C/C@S exhibits a substantially lower capacity of only 1056.5 mAh g^−1^. The apparently high specific capacity implies a superb utilization efficiency for sulfur species and excellent reversibility in virtue of the moderate affinity to the NaPSs and the effectively catalytic capability of v_6_Fe–Mo_2_C/C in the cathode. It can be further attributed to the distinct modulation for the electronic structure induced by Fe-doping and Mo-vacancies, respectively, which gives rise to the differences in the band structures of adjacent Mo_2_C crystallites, thereby driving spontaneous migration of electrons and holes across the interface and endowing the interfacial sites with high activity. More importantly, rate performance analysis (Figs. 4e and S18c) shows that v_6_Fe–Mo_2_C/C@S delivers the specific capacity up to 1337.0 mAh g^−1^ at 1 A g^−1^, with 88.8% of its initial capacity at 0.1 A g^−1^. Under the same background, v_12_Fe–Mo_2_C/C@S retains 1047.1 mAh g^−1^ (79.8%) and v_0_Fe–Mo_2_C/C@S reaches only 755.8 mAh g^−1^ (73.0%). Upon reverting from high current density back to 0.1 A g^−1^, v_6_Fe–Mo_2_C/C@S recovers to 94.9% (1429.3 mAh g^−1^) of its initial capacity, whereas v_12_Fe–Mo_2_C/C@S and v_0_Fe–Mo_2_C/C@S recover only 81.8% (1044.6 mAh g^−1^) and 74.7% (773.9 mAh g^−1^). The superior rate performance of v_6_Fe–Mo_2_C/C@S cathode, surpassing that of samples modified solely by Fe-doping or by Mo-vacancies, can be well explained by the internal electric field (IEF) formation and fast S–S bonds breakage facilitated by highly active sites on the homojunction interface. At the homojunction interface between adjacent Mo_2_C crystallites individually modified by Fe-doping and Mo-vacancies, voluntary carrier transport terminates upon alignment of the Fermi levels, with energy band bending, ultimately leading to the accumulation of opposite charge on either side of the interface and the formation of so-called IEF. Such IEF provides an additional driving force to promote both electron and ion transport, enabling their rapid access to active sites to participate in the conversion reactions of NaPSs, which finally manifests as the considerably accelerated conversion kinetics. Furthermore, the effective immobilization of NaPSs markedly suppresses the shuttling effects, preventing the loss of active species. And the boosted sulfur utilization can be enabled by a larger discharge depth to a certain degree. As a result, after 1000 cycles at 1 A g^−1^, v_6_Fe–Mo_2_C/C@S retains as high as 94.2% of its initial capacity, while the capacity retention of v_12_Fe–Mo_2_C/C@S decreases markedly with only 76.4%, not to mention that v_0_Fe–Mo_2_C/C@S preserves merely 65.4% (Figs. 4g and S24). Across all evaluated metrics, including the specific capacity, rate and cycling capability, the v_6_Fe–Mo_2_C/C@S cathode exhibits the best electrochemical performance, far outperforming that of v_0_Fe–Mo_2_C/C@S and v_12_Fe–Mo_2_C/C@S. Accordingly, it can be concluded that the homojunction construction synergistically induced by Fe-doping and Mo-vacancies is fundamentally responsible for the enormous performance enhancement.

The above observed differences in electrochemical performance are also related to the microstructure variations of host materials. As confirmed by nitrogen adsorption–desorption characterization (Figs. 2j and S10), with the enhanced etching from 0 to 6 h, the specific surface area (S_BET_) significantly increases, reaching its maximum value of 99.8 m^2^ g^−1^ in the v_6_Fe–Mo_2_C/C sample that features a broad pore-size distribution and a larger pore amount, encompassing both narrow micropores and small mesopores. However, excessive etching leads to structural degradation, causing a decrease in S_BET_ of the v_12_Fe–Mo_2_C/C sample. Particularly, the highest S_BET_ of v_6_Fe–Mo_2_C/C allows for the exposure of more active sites for NaPS adsorption. The restriction for NaPSs effectively suppresses the shuttling, directly preventing the loss of active species and consequently promoting the breakage of chemical bonds that can be regarded as a key factor in triggering the conversion reactions. Furthermore, the favorable pore structure of v_6_Fe–Mo_2_C/C plays a crucial role in enhancing its electrochemical performance. Its narrow micropores contribute to a larger active surface for charge storage, while the abundant mesopores render the open ion migration pathways. Combined with the improved conductivity caused by the homojunction structure and the additional electron conduction routes provided by the carbon substrate, these features facilitate the timely transport of electrons and ions to the reaction sites and then enable efficient participation in the redox reactions. Besides, the increased pore amount with enlarged room would more effectively alleviate the volume changes during the charge and discharge processes. Consequently, these enormously accelerate the conversion reaction kinetics, increase discharge depth, and promise the full utilization of sulfur, ultimately resulting in an exceptionally high specific capacity (1508 mAh g^−1^) at 0.1 A g^−1^, the optimal rate and cycling performance of v_6_Fe–Mo_2_C/C@S cathode.

The development of fast-charging batteries capable of being charged within a few minutes is critical for enhancing the device usability and convenience. The corresponding test of Na–S battery with v_6_Fe–Mo_2_C/C@S cathode is described in Fig. 4i, where this battery is discharged at a constant rate 0.1 A g^−1^ and charged at gradually increased current densities. Thereinto, the rate capability, expressed as the specific capacity retained even at high charge current, can be regarded as the key indicator for assessing the fast-charging performance [105]. The excellent discharge capacity retention is obtained under progressively increased charging current from 0.1, 0.2, 0.5, 1.0, 2.0 to 5.0 A g^−1^, where the disparity in discharge time is tiny but the difference in charge time is extremely obvious (Fig. 4j–k). Noteworthy, under the condition that the charging process can be finished in 17 min, the battery can deliver about 1357.0 mAh g^−1^ when discharging at 0.1 A g^−1^, implying the ultralong discharging time upon 814 min. Moreover, the outstanding cycling capability of this system is also demonstrated by the high specific capacity upon returning to 0.1 A g^−1^, which renders this battery with v_6_Fe–Mo_2_C/C@S cathode significantly practical values.

### Catalytic Activities and Kinetics Analysis

**Fig. 5:**
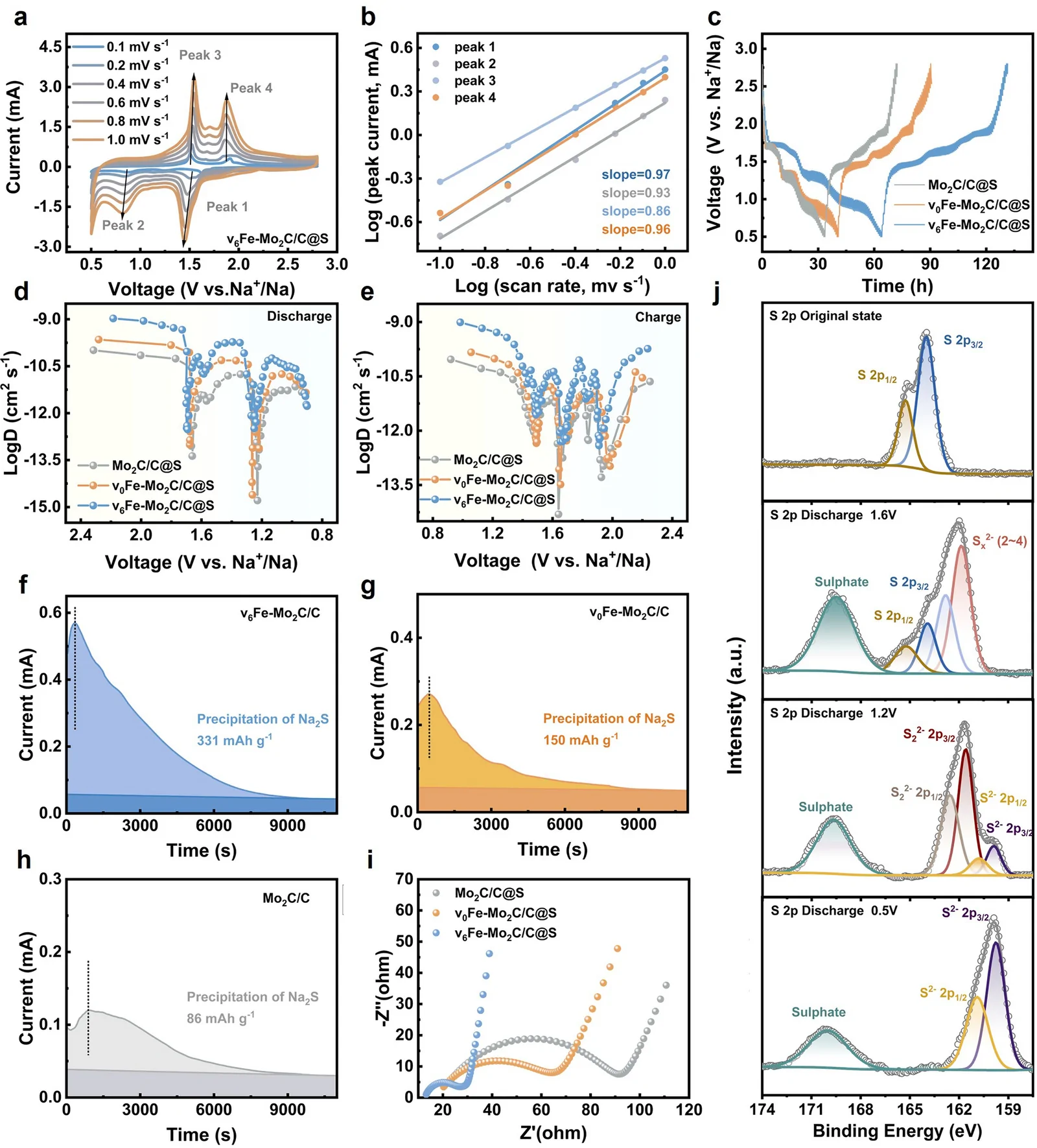
Redox reaction kinetics characterization and analysis. For v_6_Fe–Mo_2_C/C@S cathode: **a** Multi-rate scan CV curves. **b** Linear fitting between log i and log ν of the cathodic/anodic peaks. **c** Voltage variations of GITT profiles. Diffusion coefficient of Na^+^ for the **d** sodiation and **e** desodiation processes. Potentiostatic nucleation profiles of Na_2_S on **f** v_6_Fe–Mo_2_C/C, **g** v_0_Fe–Mo_2_C/C, **h** Mo_2_C/C. **i** Nyquist plots. **j** Ex situ XPS spectra of the v_6_Fe–Mo_2_C/C@S cathode measured at different discharge states

Out of the considerations that the incomplete redox conversion and low utilization rate can be attributed to the awful conductivity as well as the high reaction barrier, a series of reaction kinetics analyses were conducted in later work. First of all, CV curves under various scanning rates were collected to elucidate the mechanism of the excellent rate performance (Fig. 5a). Thereinto, the reduction peaks are observed to slightly shift toward lower voltage while the oxidation peaks slightly move toward higher voltage with the increased scan rate, which results from the increased polarization of the cell at high scan rates. In theory, the alterable b-value in power law i_p_ = av^b^ is close to 0.5 or 1, signifying that the charge storage is diffusion-controlled processes or capacity-controlled behavior, respectively, where i_p_ represents the peak current (A), ν represents the scan rate (V s^−1^), both a and b are constant parameters. Notably, the fitted b values of the battery with v_6_Fe–Mo_2_C/C@S electrode are about 0.97, 0.93, 0.86, and 0.96 assigned to peaks 1, 2, 3, and 4, respectively, delivering a capacitive process of v_6_Fe–Mo_2_C/C@S-based battery (Fig. 5b). In terms of the sharpest slopes among other systems (Fig. S25), the fastest Na^+^ diffusion dynamics regarding v_6_Fe–Mo_2_C/C@S cathode during the redox reactions can be unveiled. As is known to all, the transport kinetics characteristic of Na^+^ is mainly governed by the accumulation amount of insulating Na_2_S_2_/Na_2_S on the electrode and electrolyte viscosity susceptible to the soluble-NaPSs concentration, with the latter emerging as a critical limiting factor to dictate overall ionic transmission efficiency. That is to say, the unique n-p homostructural v_6_Fe–Mo_2_C/C host with the sustaining band bending brings its superiority into full play, such as high-efficiency electrocatalytic activity and attractive entrapping ability for the sulfur-based intermediates, thereby ensuring the fast ions diffusion, providing strong impetus to achieve high-depth discharge and protect the system from shuttling effects, finally displaying the desired reaction dynamics process. Moreover, the capacitive contribution is precisely evaluated at each given scan rate based on the equation of i_p_ = k_1_v + k_2_v^1/2^, where k_1_v and k_2_v^1/2^ refer to the reaction-controlled contribution and diffusion-controlled contribution, respectively [10, 41]. The reaction-controlled capacity contribution of v_6_Fe–Mo_2_C/C@S cathode to the total reversible capacity at the scan rate of 1 mV s^−1^ far exceeds those of v_0_Fe–Mo_2_C/C@S and Mo_2_C/C@S, once again embodying its advantages of rapid charge transfer kinetics due to the p-n homojunction effects, as shown in Fig. S26. And the calculated reaction-controlled contribution is as high as 90.4% even at a low scan rate of 0.1 mV s^−1^. In addition, the measurably increased proportion of reaction-controlled contribution, along with the sweeping rate rising, and the highest current response toward the v_6_Fe–Mo_2_C/C@S electrode strongly confirm the favorable energy storage behavior with ultrafast kinetics of Na^+^ manifested by the apparent reaction-controlled process. By contrast, the v_0_Fe–Mo_2_C/C@S and Mo_2_C/C@S cathode displays overall lower reaction-controlled contribution, which is in the range of 58.0%–83.2% and 51.1%–75.1%, respectively, at the same scan rates. This merit of v_6_Fe–Mo_2_C/C@S cathode extremely accelerates the sodiation/desodiation reactions and enables a fast-charge-storage process, especially at high rates (Fig. S26b) [106].

To gain deep insight into the enhanced reaction kinetics and further elaborate the mechanism of how to achieve the fast-charging performance by “induced-homojunction”, galvanostatic intermittent titration technique (GITT) was carried out. As described in Fig. 5c, the following sodiation and desodiation processes of v_6_Fe–Mo_2_C/C@S cathode present higher capacities compared with those of Mo_2_C/C@S and v_0_Fe–Mo_2_C/C@S cathodes, demonstrating the improved NaPSs adsorption performance, higher ion diffusion and active species utilization efficiency, which coincides with preceding results in Fig. 4. Thereinto, the corresponding illustrations of the typical single-step charge/discharge GITT curve signed with Δ*E*_s_, Δ*E*_τ_, and IR drop are given (Fig. S27), which is used for better understanding the measurement process and calculating the following diffusion coefficient of Na^+^ (D_Na+_) based on Fick’s second law [107]. Attentionally, the profiles toward D_Na+_ of various cathodes in Fig. 5d, e exhibit peaks at about 1.68 and 1.25 V throughout the discharge procedure, assigned to the reduction process from long-chain Na_2_S_n_ (4 < n ≤ 8) to Na_2_S_4_, and the step to short-chain Na_2_S_2_/Na_2_S. Likewise, the apparent charging peaks are indexed to the reversible and stepwise oxidation process. In particular, although v_0_Fe–Mo_2_C/C@S exhibits boosted D_Na+_ within the discharge range (> 1.30 V) and charge region (1.60–1.96 V), its reaction sites manifest analogous Na⁺ diffusion behavior to Mo_2_C/C@S during subsequent discharge process corresponding to the voltage around 1.25 V, and the charge process at the voltage around 1.49 V. This similarity testifies intrinsic high-energy barriers toward the conversion to short-chain sodium polysulfides in both v_0_Fe–Mo_2_C/C@S and Mo_2_C/C@S cathodes, resulting in considerable hindrance for the Na_2_S formation and decomposition [103]. As a stark contrast, the evidently boosted D_Na+_ during the overall charge and discharge processes of v_6_Fe–Mo_2_C/C@S electrode directly manifests the extraordinary migration kinetics of Na^+^. And then the superior Na^+^ conductivity, particularly in the range assigned to Na_2_S nucleation and activation process closely relevant to the complete redox reactions, guarantees the immense decreases in the charge–discharge reaction resistances, which in turn realizes the kinetically favorable energy storage process and shows great significance for ultrafast charging Na–S battery. These results above also strongly confirm that only under the interfacial p-n effects with the energy band bending synergistically created by doped-Fe and Mo-vacancies, can the rapid migration of Na^+^ and high-efficiency redox reactions be ensured throughout the entire energy storage process, which can be explained by the increased charge density due to the directional carrier migration and the bidirectional IEF drive for charge. Considering the importance of the step toward the final product Na_2_S, the catalytic capability of various host materials is evaluated via the potentiostatic nucleation experiments. Based on Faraday’s law, the highest deposition capacity (331 mAh g^−1^) of v_6_Fe–Mo_2_C/C catalyst is identified, far exceeding that of v_0_Fe–Mo_2_C/C (150 mAh g^−1^) and Mo_2_C/C (86 mAh g^−1^). And as depicted in Fig. 5f–h, the highest current response of the v_6_Fe–Mo_2_C/C sample and the lowest value of the Mo_2_C/C sample suggest the faster deposition rate on the former electrode. Integrating the merit that the Na_2_S deposition curve toward v_6_Fe–Mo_2_C/C material peaks fastest, it can be asserted that the conversion process to insoluble Na_2_S occurs earliest, implying the best electrocatalytic ability of the v_6_Fe–Mo_2_C/C sample and the most favorable conversion process among NaPSs [18, 108].

Subsequently, electrochemical impedance spectroscopy (EIS) was tested to ulteriorly explore the surface state and reaction kinetics properties of RT Na–S cells with different cathodes. The Nyquist plot of v_6_Fe–Mo_2_C/C@S cathode exhibits much lower charge transfer resistance (R_ct_) represented by the half-circle than other cathodes, signifying the faster dynamics behavior and a more stable surface (Fig. 5i). Likewise, the noticeably large resistance of Mo_2_C/C@S and v_0_Fe–Mo_2_C/C@S cathodes can be interpreted by the inherent electronic insulation and interface incompatibility. Moreover, the lowest Ohmic resistance (R_s_) represents the reduced Ohmic polarization and the smallest Warburg impedance (Z_w_) value implies the v_6_Fe–Mo_2_C/C@S electrode achieves the best solid-state diffusion kinetics of Na^+^ in the electrode architecture compared with other systems. Thereinto, the electronic and ionic conduction cannot be sufficiently improved by the doping-strategy alone and the resulting catalytic performance exhibits a huge gap compared to that observed in the presence of p-n homojunction. Besides, the symmetrical batteries with 0.2 M Na_2_S_6_ electrolyte were also assembled with identical electrodes on both sides to further investigate the catalytic capability of Mo_2_C/C, v_0_Fe–Mo_2_C/C and v_6_Fe–Mo_2_C/C on the conversion of NaPSs. It can be seen that v_6_Fe–Mo_2_C/C explicitly displays a pair of reversible redox peaks with the highest current response and the smallest redox potential gap in the − 1.5–1.5 V voltage window compared with that of other hosts, affirming the enhanced electrocatalytic activity of v_6_Fe–Mo_2_C/C based on the sandwiched p-n homojunction (Fig. S28a). Meanwhile, the EIS plots (Fig. S29) tested for symmetrical batteries disclose that v_6_Fe–Mo_2_C/C owns a distinctly lowest interfacial charge transfer resistance (R_ct_), which can be attributed to the competitive electronic conductivity, thus making contributions to affording excellent electrocatalytic activity for highly effective NaPSs transformation and competitive fast-charging capability [109].

### Electrochemical Mechanism of Designed Cathode

The ex situ XPS measurements of the v_6_Fe–Mo_2_C/C@S cathode are conducted to further clarify the sulfur conversion mechanism at different voltage states (Fig. 5j). As for the original cathode, two peaks at 165.3 and 164.0 eV are assigned to the spin–orbit coupling of S 2*p*_*3/2*_ and S 2*p*_*1/2*_ in elemental S. For the first discharge to 1.6 V, two new peaks located at 162.9 and 161.9 eV could be attributed to Na_2_S_*x*_ (2 ≤ x ≤ 4), in accompany with the weakened signal of elemental S during the discharge process. As anticipated, sulfur-intermediate is further reduced at a deeper discharge depth and the characteristic S signal thoroughly disappears. Upon discharging to 0.5 V, only the peak of Na_2_S at 161.0 and 159.8 eV can be observed without other NaPSs, implying the high S utilization [53, 85].

**Fig. 6:**
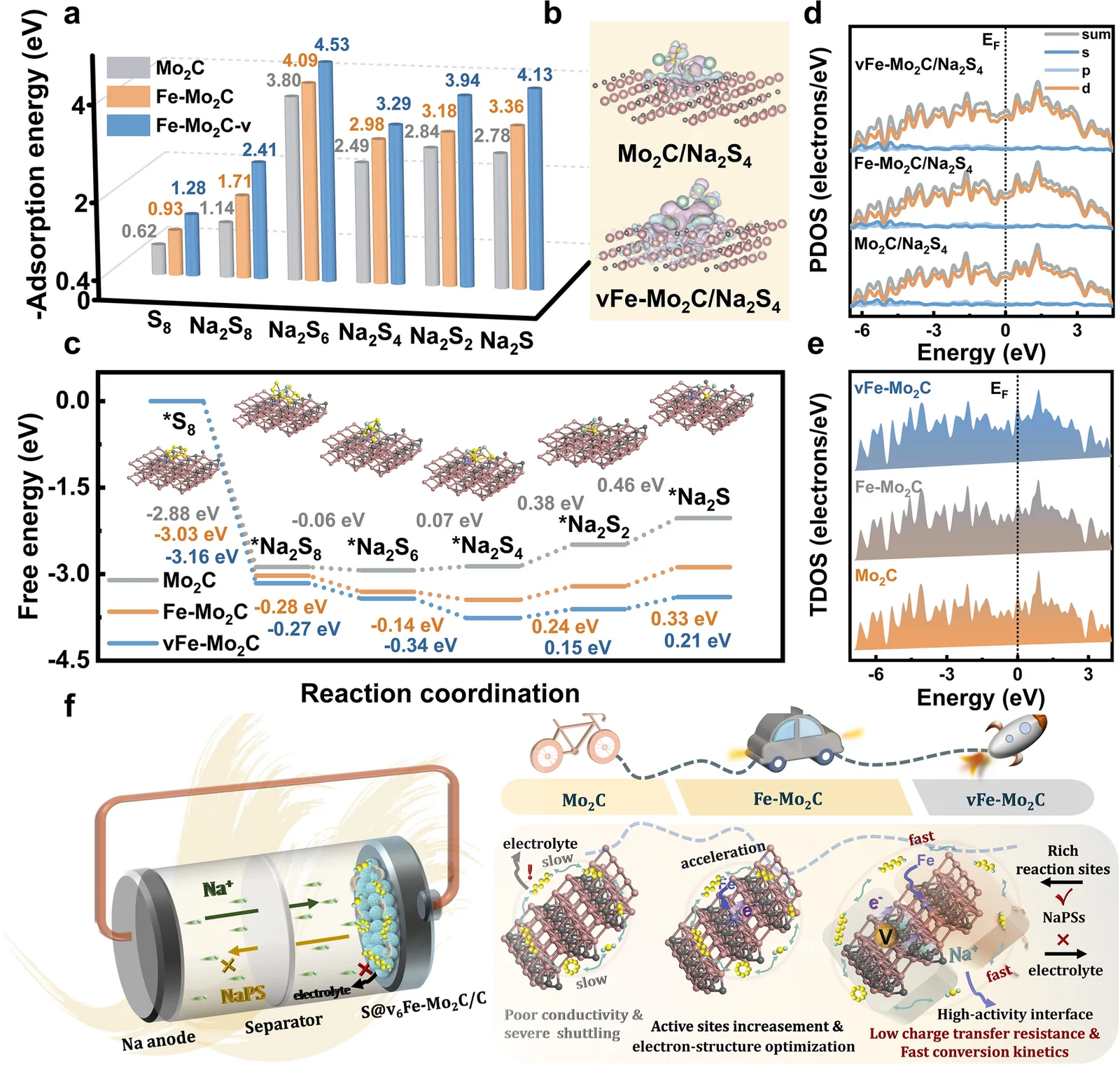
Theoretical simulations of the electronic structure, Na_2_S_n_ dissociative adsorption and conversion process: **a** Adsorption energies of S_8_ and NaPSs on Mo_2_C, Fe–Mo_2_C and vFe–Mo_2_C hosts. **b** Charge-density difference of Na_2_S_4_ adsorbed on Mo_2_C or vFe–Mo_2_C, blue and purple represent charge accumulation and loss, respectively. **c** Gibbs free energy profiles of reduction step from S_8_ to final Na_2_S on Mo_2_C, Fe–Mo_2_C and vFe–Mo_2_C hosts. The optimized adsorption conformations of NaPSs on vFe–Mo_2_C are illustrated in the insets. (pink, gray, purple, light green, and light yellow balls represent molybdenum, carbon, iron, sodium, and sulfur atoms, respectively). **d** PDOS of Na_2_S_4_ adsorbed on Mo_2_C, Fe–Mo_2_C and vFe–Mo_2_C. **e** TDOS for Mo_2_C, Fe–Mo_2_C and vFe–Mo_2_C hosts. **f** Schematic illustrations: the electrocatalytic mechanism and advantages of the vFe–Mo_2_C host in energy storage processes based on Na–S batteries

First-principles calculations based on the density functional theory (DFT) method were applied to give deep insight into the mechanism of how the homojunction induced by co-implantation of Fe and Mo-vacancy regulates the electronic structure and then noticeably ameliorates the kinetics behavior of the overall energy storage process to achieve the concurrent optimization of storage capacity, rate performance and fast-charging ability. For the purpose of highlighting the advantages of this “induced-homojunction” strategy, theoretical models of pristine Mo_2_C, Fe-doped Mo_2_C and Mo_2_C jointly decorated by vacancies and Fe-atom were established, denoted as Mo_2_C, Fe–Mo_2_C and vFe–Mo_2_C, respectively. The synergistic improvement mechanism of Fe–Mo_2_C-v for boosting the electrochemical performance toward Na–S cells can be visualized by the interaction between various Mo_2_C-based hosts above and all involved intermediates of Na_2_S_n_ (n = 1, 2, 4, 6, 8). Different from the reflected poor affinity to NaPSs of pristine Mo_2_C, the adsorption energies (E_ads_) of S_8_, Na_2_S_8_, Na_2_S_6_, Na_2_S_4_, Na_2_S_2_, and Na_2_S on vFe–Mo_2_C are calculated to be − 1.28, − 2.41, − 4.53, − 3.29, − 3.94, and − 4.13 eV, respectively, where these obviously more negative E_ads_ throughout the whole reaction processes than that assigned to Fe–Mo_2_C signify better and moderate chemical immobilization ability for the sodium polysulfides in accompany with the favorable formation of Mo–S bonds, thereby substantially weakening S–S bonds and promoting their eventual reactions. Besides, the moderate catalyst-intermediate interaction does not impose a significant barrier for product desorption, thereby enabling highly efficient catalytic cycling while avoiding catalyst deactivation, as depicted in Figs. 6a and S30. As demonstrated in charge-density difference maps, the vFe–Mo_2_C system with Na_2_S_4_ adsorption exhibits an apparently elevated electron cloud density than that of other two systems of Fe–Mo_2_C–Na_2_S_4_ and Mo_2_C–Na_2_S_4_, giving direct evidence for the stronger interaction between Fe–Mo_2_C-v substrate and active species (Figs. 6b and S31). This finding is in line with the binding energy values, which collectively testifies the homojunction construction can effectively prompt the carrier transmission across the interface in view of the continuous band bending within the same constitution, and then empower the interface between n-type and p-type semiconductor with high activity to fast bind with NaPSs, ultimately obviously increasing the charge-storage capacity. The progressively decreased binding energies observed across Mo_2_C to vFe–Mo_2_C hosts correlating with increasingly favorable thermodynamics processes, perfectly align with the gradual clarification of supernatants in adsorption experiments and continuously raised specific capacity. Compared with the only reaction sites introduction with localized electron-state modulation to a certain extent by the single Fe-doping or single Mo-vacancy mean, the enhanced energy storage capability via homojunction effects is more highlighted.

A favorable adsorption process between hosts and various-order active species can be regarded as the critical step to trigger the redox reactions. To investigate the correlation among effectual adsorption, intrinsic electrocatalytic activity and the significantly enhanced reaction kinetics by inducing p-n Mo_2_C homojunction, the Gibbs free energies of stepwise sulfur evolution pathways on the individual catalytic substrates are systematically evaluated based on the optimal configurations (Fig. 6c). Specifically, a larger energy gain is expected in the gradual sodiation process for Na_2_S_n_ when a more negative ΔG is obtained. That is to say, there is a driving force during the exergonic process for facilitating Na^+^ to migrate into a Na-poor region and bind with a Na-deficient Na_2_S_n_ chain. Particularly, the ΔG value toward the Na_2_S_2_/Na_2_S precipitation is dramatically decreased on vFe–Mo_2_C surface, which is quantified as 0.21 eV than that of Fe–Mo_2_C (0.33 eV) and pristine Mo_2_C (0.46 eV), while the free energy levels of other reaction steps on vFe–Mo_2_C catalyst always remain minimized throughout the discharge process, collectively ensuring thermodynamically more favorable processes regardless of long-chain or short-chain NaPSs conversion. The geometric structures of relative configurations are provided in the inset of Figs. 6c and S30. Notably, the Na_2_S_2_ adsorption on vFe–Mo_2_C (101) imposes a conspicuously increased S–S bond length (2.71 Å) compared with that on Fe–Mo_2_C (101) (2.42 Å) and Mo_2_C (101) (2.24 Å), signifying a greater tendency for S–S bond cleavage and the easier sulfur-based redox reactions, which benefits from the enhanced interactions with NaPSs by means of the preferred n-p elecronic transmission across homojuntion interface [37].

Considering the conductivity of the host/NaPSs systems after adsorption is vital for subsequent sulfur-based species transformation, the partial density of states (PDOS) calculation was employed to probe the corresponding electronic structures of adsorption systems (Fig. 6d). Encouragingly, a monotonically decreased trend toward electron cloud density near the Fermi level from vFe–Mo_2_C/Na_2_S_4_ to Fe–Mo_2_C/Na_2_S_4_ to Mo_2_C/Na_2_S_4_ systems further affirms that the “induced-homojunction” by the coexistence of Mo-vacancy and Fe-dopant can significantly enhance charge transfer between the catalyst and NaPSs, thereby dramatically favoring the fast kinetics of catalyst-assisted sulfur-redox reactions [110, 111]. These observations agree well with the analytic results of ΔG. Moreover, the metallic characteristics have been corroborated by the continuous distribution of electronic states across the Fermi level in Fig. 6e. And the superior electronic conductivity in the form of the highest total density of states (TDOS) at the Fermi level of vFe–Mo_2_C than that of other hosts including Fe–Mo_2_C and pristine Mo_2_C has been verified, which affords more electrons to participate in reactions and compensates for the inherent insulation drawback of sulfur, thus surely playing a decisive role in accelerating the discharge/charge kinetics of Na–S batteries. The enhancement of conductivity by homojunction has been fully demonstrated here. The above analysis fully demonstrates that constructing the structure with p-type conductive characteristics near the n-type semiconductor within the individual Mo_2_C can successfully trigger the interfacial n-p homojunction effects, thereby achieving the goal of enhancing conductivity. To further substantiate the charge-accumulation situations, the calculation of charge-density difference was performed (Fig. S32). Compared with the Fe–Mo_2_C model only with Fe-doping, the coexistent vFe–Mo_2_C model shows a more pronounced distribution of oppositely charged species on the two sides of the potential interface between the Fe-doped local structure and the Mo-vacancy local structure, which fully matches the IEF analysis. These phenomena match well with the results of XPS and Raman characterizations. It can be believed that the introduction of “induced-homojunction” concept could exert enormous effects on accelerating the charge-storage dynamics based on the polysulfide-intermediated sulfur redox, overcoming the most fundamental obstacles in Na–S battery applications and providing valuable insights for designing fast-charging sulfur cathodes.

Incorporating the experimental results and DFT calculations together, the working mechanisms toward “induced-homojunction” via co-implantation design to ameliorate sulfur-based conversion kinetics and enhance the fast-charging performance are visually depicted in Figs. 3g and 6f. The Fe-dopants and Mo-vacancies via acid-etching, introduced as additional active sites, individually tune the surrounding electronic configurations, resulting in discernible energy level disparities and different p- or n-conductive type between adjacent crystalline domains of Mo_2_C, which in turn constructs the homojunction structure. More specifically, inducing p-type Mo_2_C semiconductor via Fe-doping around n-type Mo_2_C with Mo-vacancy to prepare a kind of sandwiched p-n homojunctions. The fast and directional carrier migration with continuous band bending occurs in the formation process of the sandwiched p-n homojunctions, enabling considerably enhanced charge density on each binding site. In particular, the prominent charge localization at sites not only appropriately amplifies the interactions with various-order NaPSs to ensure their rapid and efficient confinement at multi-active centers but also facilitates the conductive Mo-S bonds formation to provide eminent electron transfer pathways while accelerating the breakage of inherent S–S bonds, which in turn initiates the rapid redox reactions. Meanwhile, the enhanced chemical immobilization for NaPSs and their fast transformation to insoluble Na_2_S_2_/Na_2_S efficaciously avoid the shuttling effects of long-chain polysulfides and the damage of active species. Furtherly, the migration behavior of electrons and holes culminates in Fermi level alignment, leading to spatial-charge accumulation with opposing polarities at the interface sides, along with the establishment of so-called internal electric fields (IEF) on the homojunction interfaces. Under the driving functions of IEF, electrons and Na^+^ vectorially transfer to react with NaPSs as quickly as possible, which has been strongly corroborated by the substantially boosted electron conductivity, reduced Eads and ΔG in DFT calculations, the remarkable reduction of the energy barriers corresponding to Na_2_S_2_/Na_2_S formation step. Consequently, the ingenious construction of p-n homojunction enables the high discharge depths and significantly improves utilization efficiency for sulfur, ultimately achieving advanced RT Na-S batteries with unparalleled reaction dynamics advantage and fast-charging capability. This insight offers a new paradigm, implying the possibility that other strategies capable of modulating the electronic structure can be synergistically employed to construct highly active interfaces and extended to a broad range of materials.

## Conclusions

In short, a new “induced-homojunction” concept is introduced to Na-S batteries firstly, where the sandwiched p-n Mo_2_C homojunction is constructed as the sulfur host (v_6_Fe–Mo_2_C/C) for improving the conversion kinetics process. It is the respective modulations of Fe-doping and Mo-vacancy for the electronic structure around these sites that endow the Mo_2_C with n- and p-type conductivities, and make it possible to construct the p-n homojunction. The tendency toward energy band alignment apparently facilitates the directional spatial-charge transmission across the interface, resulting in the increased charge density and potential gradient at both sides of the interface, i.e., the built-in electric field (IEF). In virtue of the unique homojunction with continuous band bending, the sites are empowered with high activity to remarkably enhance their entrapment ability for polysulfides, ultimately enabling the efficient cleavage of S–S bonds and then initiating the conversion reactions. Furthermore, the drive by IEF achieves fast electrons and ions migration to timely supply for subsequent redox reactions, hence considerably improving the catalytic conversion kinetics with the rapid Na_2_S nucleation/decomposition and exhibiting the fast-charging advantage. As a result, the battery assembled by v_6_Fe–Mo_2_C/C@S cathode delivers a satisfactory capacity of 1508 mAh g^−1^ at 0.1 A g^−1^ even over 100 cycles and long-term storage life. Especially the almost top-tier rate capability with 88.8% capacity retention (1 A g^−1^) and the fast-charging ability delivering a stable discharge capacity (1357 mAh g^−1^) independently from the charging rate (even at 5 A g^−1^), endow its with great practical value. The proposition of “induced-homojunction” concept in this work is of great significance for the high-efficiency electrocatalyst design and exploitation of fast-charging Na–S batteries.

## Supplementary Information

Below is the link to the electronic supplementary material.

Supplementary file1 (DOCX 8694 kb)

## References

[1] M. Qin, M. Liu, Z. Zeng, Q. Wu, Y. Wu et al., Rejuvenating propylene carbonate-based electrolyte through nonsolvating interactions for wide-temperature Li-ions batteries. Adv. Energy Mater. **12**(48), 2201801 (2022). 10.1002/aenm.202201801

[2] M. Fichtner, K. Edström, E. Ayerbe, M. Berecibar, A. Bhowmik et al., Rechargeable batteries of the future: the state of the art from a BATTERY 2030+ perspective. Adv. Energy Mater. **12**(17), 2102904 (2022). 10.1002/aenm.202102904

[3] X. Zeng, M. Li, D. Abd El-Hady, W. Alshitari, A.S. Al-Bogami et al., Commercialization of lithium battery technologies for electric vehicles. Adv. Energy Mater. **9**(27), 1900161 (2019). 10.1002/aenm.201900161

[4] Y.-J. Lei, H.-W. Liu, Z. Yang, L.-F. Zhao, W.-H. Lai et al., A review on the status and challenges of cathodes in room-temperature Na-S batteries. Adv. Funct. Mater. **33**(11), 2212600 (2023). 10.1002/adfm.202212600

[5] J. Wu, Y. Tian, Y. Gao, Z. Gao, Y. Meng et al., Rational electrolyte design toward cyclability remedy for room-temperature sodium–sulfur batteries. Angew. Chem. Int. Ed. **61**(30), e202205416 (2022). 10.1002/anie.202205416 10.1002/anie.202205416 35538589

[6] Y.-X. Wang, B. Zhang, W. Lai, Y. Xu, S.-L. Chou et al., Room-temperature sodium-sulfur batteries: a comprehensive review on research progress and cell chemistry. Adv. Energy Mater. **7**(24), 1602829 (2017). 10.1002/aenm.201602829

[7] Z.W. Seh, J. Sun, Y. Sun, Y. Cui, A highly reversible room-temperature sodium metal anode. ACS Cent. Sci. **1**(8), 449–455 (2015). 10.1021/acscentsci.5b00328 27163006 10.1021/acscentsci.5b00328 PMC4827673

[8] Y. Wang, X.L. Huang, H. Liu, W. Qiu, C. Feng et al., Nanostructure engineering strategies of cathode materials for room-temperature Na–S batteries. ACS Nano **16**(4), 5103–5130 (2022). 10.1021/acsnano.2c00265 35377602 10.1021/acsnano.2c00265

[9] Z. Ahaliabadeh, V. Miikkulainen, M. Mäntymäki, M. Colalongo, S. Mousavihashemi et al., Stabilized nickel-rich-layered oxide electrodes for high-performance lithium-ion batteries. Energy Environ. Mater. **7**(6), e12741 (2024). 10.1002/eem2.12741

[10] Y. Fan, D. Su, Y. Zheng, Q. Le, D. Chen et al., Anionic MOF-derived Ni/Ni_1-__*x*_O heterojunctions with electrochemically induced vacancy reconstruction: enabling high-rate and stable room-temperature Na–S batteries. Adv. Energy Mater. **16**(2), e04080 (2026). 10.1002/aenm.202504080

[11] Z. Lin, Z. Xu, Y. Ying, G. Chen, X. Gong et al., Nano-particulate surface pinning of CeO_2_ enables durable high-voltage lithium-ion batteries. Adv. Mater. **38**(3), e17074 (2026). 10.1002/adma.202517074 41045120 10.1002/adma.202517074

[12] Z. Lin, Y. Ying, Z. Xu, G. Chen, X. Gong et al., A multifunctional zeolite film enables stable high-voltage operation of a LiCoO_2_ cathode. Energy Environ. Sci. **18**(1), 334–346 (2025). 10.1039/d4ee04370g

[13] G. Li, J. Sun, W. Hou, S. Jiang, Y. Huang et al., Three-dimensional porous carbon composites containing high sulfur nanoparticle content for high-performance lithium–sulfur batteries. Nat. Commun. **7**, 10601 (2016). 10.1038/ncomms10601 26830732 10.1038/ncomms10601 PMC4740444

[14] H. Liu, J. Long, H. Yu, S. Zhang, S. Shi et al., Synergistic engineering of Ni-decorated composite current collector and hollow CoS nanocages for ultrahigh areal capacity lithium-ion battery anodes. ACS Sustain. Chem. Eng. **14**(3), 1529–1537 (2026). 10.1021/acssuschemeng.5c10890

[15] B. Guo, W. Du, T. Yang, J. Deng, D. Liu et al., Nickel hollow spheres concatenated by nitrogen-doped carbon fibers for enhancing electrochemical kinetics of sodium–sulfur batteries. Adv. Sci. **7**(4), 1902617 (2020). 10.1002/advs.201902617 10.1002/advs.201902617 PMC7029643 32099760

[16] Q. He, W. Chen, B. Fan, Q. Wei, Y. Zou, Heteroatom-doped ZIF-67 for anchoring and catalyzing polysulfides in lithium–sulfur batteries. Chem. Eng. J. **496**, 153813 (2024). 10.1016/j.cej.2024.153813

[17] Y. Chen, Y. Liao, L. Li, Y. Ding, Y. Wu et al., Solvothermal-assisted defect engineering in hierarchically porous carbonized wood fibers for high-performance lithium–sulfur batteries. Green Chem. **27**(16), 4235–4243 (2025). 10.1039/d5gc00414d

[18] S. Zhang, Y. Yao, X. Jiao, M. Ma, H. Huang et al., Mo_2_N–W_2_N heterostructures embedded in spherical carbon superstructure as highly efficient polysulfide electrocatalysts for stable room-temperature Na–S batteries. Adv. Mater. **33**(43), 2103846 (2021). 10.1002/adma.202103846 10.1002/adma.202103846 34463381

[19] B. Yu, C. Gyan-Barimah, J. Wang, M.I. Maulana, J.H. Sung et al., Magnesiothermically synthesized TiO-decorated 3D N-doped graphitized porous carbon as a multifunctional sulfur host for Li–S batteries. ACS Nano **19**(43), 37879–37894 (2025). 10.1021/acsnano.5c11688 41120190 10.1021/acsnano.5c11688

[20] Y. Dong, T. Li, D. Cai, S. Yang, X. Zhou et al., Progress and prospect of organic electrocatalysts in lithium–sulfur batteries. Front. Chem. **9**, 703354 (2021). 10.3389/fchem.2021.703354 34336789 10.3389/fchem.2021.703354 PMC8322034

[21] H. Raza, J. Cheng, J. Xu, L. An, J. Wang et al., Harnessing high entropy sulfide (HES) as a robust electrocatalyst for long-term cycling of lithium-sulfur batteries. ENERGY & ENVIRONMENTAL MATERIALS **8**(4), e70007 (2025). 10.1002/eem2.70007

[22] Z. Yang, Y. Wang, J. Han, T. Wu, Y. Fu et al., Exclusive Se-O coordination and Fe-doping complementation: a catalytic strategy for enhanced sulfur redox in Li-S batteries. Adv. Sci. **13**(9), e13049 (2026). 10.1002/advs.202513049 10.1002/advs.202513049 PMC12904079 41316877

[23] T.L.L. Doan, D.C. Nguyen, P.M. Bacirhonde, A.S. Yasin, A.I. Rezk et al., Atomic dispersion of Rh on interconnected Mo_2_C nanosheet network intimately embedded in 3D Ni_*x*_MoO_*y*_ nanorod arrays for pH-universal hydrogen evolution. Energy Environ. Mater. **6**(5), e12407 (2023). 10.1002/eem2.12407

[24] Y. Xie, W. Zheng, J. Ao, Y. Shao, X. Huang et al., Multifunctional Ni-doped CoSe_2_ nanoparticles decorated bilayer carbon structures for polysulfide conversion and dendrite-free lithium toward high-performance Li-S full cell. Energy Storage Mater. **62**, 102925 (2023). 10.1016/j.ensm.2023.102925

[25] S. Tao, X. Zhang, Z. Gao, T.-Y. Chen, H. Min et al., Dynamic electronic and ionic transport actuated by cobalt-doped MoSe_2_/rGO for superior potassium-ion batteries. Small **19**(48), 2304200 (2023). 10.1002/smll.202304200 10.1002/smll.202304200 37525334

[26] X. Zhang, T. Yang, J. Liu, C. Hu, S. Gao et al., Atomic-level catalyst coupled with metal oxide heterostructure for promoting kinetics of lithium-sulfur batteries. Small **20**(31), 2311086 (2024). 10.1002/smll.202311086 10.1002/smll.202311086 38459647

[27] Z. Lin, K. Fan, T. Liu, Z. Xu, G. Chen et al., Mitigating lattice distortion of high-voltage LiCoO_2_*via* core-shell structure induced by cationic heterogeneous co-doping for lithium-ion batteries. Nano-Micro Lett. **16**(1), 48 (2023). 10.1007/s40820-023-01269-1 10.1007/s40820-023-01269-1 PMC10713914 38082174

[28] G. Zhang, C. Ye, T. Li, S. Liu, W.-H. Huang et al., Quenching-induced Fe doping on spent cathode materials enhances the oxygen evolution reaction performance. Energy Storage Mater. **80**, 104430 (2025). 10.1016/j.ensm.2025.104430

[29] R. Zhang, Y. Guo, S. Zhang, D. Chen, Y. Zhao et al., Efficient ammonia electrosynthesis and energy conversion through a Zn-nitrate battery by iron doping engineered nickel phosphide catalyst. Adv. Energy Mater. **12**(13), 2103872 (2022). 10.1002/aenm.202103872

[30] J. Li, J. Yu, Y. Zhang, C. Li, Y. Ma et al., Boosting polysulfide conversion on Fe-doped nickel diselenide toward robust lithium–sulfur batteries. Adv. Funct. Mater. **35**(33), 2501485 (2025). 10.1002/adfm.202501485

[31] J. Liu, G. Li, D. Luo, J. Li, X. Zhang et al., Incorporation of heteroatomic Fe activates rapid catalytic behaviors of Co_3_O_4_ hollow nanoplates toward advanced lithium–sulfur batteries. Adv. Funct. Mater. **34**(5), 2303357 (2024). 10.1002/adfm.202303357

[32] Y. Ma, C. Fang, B. Ding, G. Ji, J.Y. Lee, Fe-doped Mn_x_O_y_ with hierarchical porosity as a high-performance lithium-ion battery anode. Adv. Mater. **25**(33), 4646–4652 (2013). 10.1002/adma.201301906 23798505 10.1002/adma.201301906

[33] D. Li, W. Wan, Z. Wang, H. Wu, S. Wu et al., Self-derivation and surface reconstruction of Fe-doped Ni_3_S_2_ electrode realizing high-efficient and stable overall water and urea electrolysis. Adv. Energy Mater. **12**(39), 2201913 (2022). 10.1002/aenm.202201913

[34] Z. Wu, Y. Zhao, W. Jin, B. Jia, J. Wang et al., Recent progress of vacancy engineering for electrochemical energy conversion related applications. Adv. Funct. Mater. **31**(9), 2009070 (2021). 10.1002/adfm.202009070

[35] X. Fu, M. Yang, M. Zhai, C. Zhang, H. Niu et al., Precision anode vacancy engineering for long-lasting and fast-charging Na-Ion batteries. Energy Storage Mater. **70**, 103450 (2024). 10.1016/j.ensm.2024.103450

[36] Q. Xu, Y. Wang, X. Feng, T. Fang, X. Li et al., Monoclinic Li_2_ZrO_3_ with cationic vacancy–based ion transport channels enhanced composite polymer electrolytes for high-rate solid-state lithium metal batteries. Nano Energy **147**, 111571 (2026). 10.1016/j.nanoen.2025.111571

[37] Y. Zhang, G. Li, J. Wang, G. Cui, X. Wei et al., Hierarchical defective Fe_3-__*x*_C@C hollow microsphere enables fast and long-lasting lithium–sulfur batteries. Adv. Funct. Mater. **30**(22), 2001165 (2020). 10.1002/adfm.202001165

[38] S. Wang, Y. Wang, Y. Song, X. Jia, J. Yang et al., Immobilizing polysulfide *via* multiple active sites in W_18_O_49_for Li-S batteries by oxygen vacancy engineering. Energy Storage Mater. **43**, 422–429 (2021). 10.1016/j.ensm.2021.09.020

[39] G. Liu, G. Zhao, W. Zhou, Y. Liu, H. Pang et al., *In situ* bond modulation of graphitic carbon nitride to construct p–n homojunctions for enhanced photocatalytic hydrogen production. Adv. Funct. Mater. **26**(37), 6822–6829 (2016). 10.1002/adfm.201602779

[40] S.-M. Wu, X.-L. Liu, X.-L. Lian, G. Tian, C. Janiak et al., Homojunction of oxygen and titanium vacancies and its interfacial n–p effect. Adv. Mater. **30**(32), 1802173 (2018). 10.1002/adma.201802173 10.1002/adma.201802173 29947064

[41] H. Hao, Y. Wang, N. Katyal, G. Yang, H. Dong et al., Molybdenum carbide electrocatalyst *in situ* embedded in porous nitrogen-rich carbon nanotubes promotes rapid kinetics in sodium-metal–sulfur batteries. Adv. Mater. **34**(26), 2106572 (2022). 10.1002/adma.202106572 10.1002/adma.202106572 35451133

[42] J.P. Perdew, K. Burke, M. Ernzerhof, Generalized gradient approximation made simple. Phys. Rev. Lett. **77**(18), 3865–3868 (1996). 10.1103/physrevlett.77.3865 10062328 10.1103/PhysRevLett.77.3865

[43] H.J. Monkhorst, J.D. Pack, Special points for Brillouin-zone integrations. Phys. Rev. B **13**(12), 5188–5192 (1976). 10.1103/physrevb.13.5188

[44] F. Wang, J. An, H. Shen, Z. Wang, G. Li et al., Gradient graphdiyne induced copper and oxygen vacancies in Cu_0.95_V_2_O_5_anodes for fast-charging lithium-ion batteries. Angew. Chem. Int. Ed. **62**(7), e202216397 (2023). 10.1002/anie.202216397 10.1002/anie.202216397 36517418

[45] K. Huang, Y. Yan, J. Li, L. Qiao, J. Sui et al., Mo vacancies enhancing Pt, Ni co-incorporated Mo_2_C nanofibers for high-efficiency water decomposition. Vacuum **231**, 113792 (2025). 10.1016/j.vacuum.2024.113792

[46] S. Siddique, G. Abbas, M.M. Yaqoob, J. Zhao, R. Chen et al., Optimization of thermoelectric performance in *p*-type SnSe crystals through localized lattice distortions and band convergence. Adv. Sci. **12**(7), 2411594 (2025). 10.1002/advs.202411594 10.1002/advs.202411594 PMC11831502 39721020

[47] M. Yuan, H. Lv, H. Cheng, B. Zhao, G. Chen et al., Atomic and electronic reconstruction in defective 0D molybdenum carbide heterostructure for regulating lower-frequency microwaves. Adv. Funct. Mater. **33**(33), 2302003 (2023). 10.1002/adfm.202302003

[48] J. Zhao, Y. Bai, X. Liang, T. Wang, C. Wang, Photothermal catalytic CO_2_ hydrogenation over molybdenum carbides: crystal structure and photothermocatalytic synergistic effects. J. CO2 Util. **49**, 101562 (2021). 10.1016/j.jcou.2021.101562

[49] Q. Lu, B. Xiao, M. Zhang, H. Sun, Q. Lu et al., Etching dopant elements to construct active-site-rich Mo_2_C for the hydrogen evolution reaction. Chem. Commun. **59**(15), 2153–2156 (2023). 10.1039/d2cc06181c 10.1039/d2cc06181c 36727577

[50] Y. Ma, M. Chen, H. Geng, H. Dong, P. Wu et al., Synergistically tuning electronic structure of porous β-Mo_2_C spheres by co doping and Mo-vacancies defect engineering for optimizing hydrogen evolution reaction activity. Adv. Funct. Mater. **30**(19), 2000561 (2020). 10.1002/adfm.202000561

[51] S. Li, C. Cheng, A. Sagaltchik, P. Pachfule, C. Zhao et al., Metal-organic precursor–derived mesoporous carbon spheres with homogeneously distributed molybdenum carbide/nitride nanoparticles for efficient hydrogen evolution in alkaline media. Adv. Funct. Mater. **29**(3), 1807419 (2019). 10.1002/adfm.201807419

[52] H. Kang, A. Washington, M.D. Capobianco, X. Yan, V.V. Cruz et al., Concentration-dependent photocatalytic upcycling of poly(ethylene terephthalate) plastic waste. ACS Mater. Lett. **5**(11), 3032–3041 (2023). 10.1021/acsmaterialslett.3c01134 37969139 10.1021/acsmaterialslett.3c01134 PMC10630977

[53] X. Zhou, Z. Yu, Y. Yao, Y. Jiang, X. Rui et al., A high-efficiency Mo_2_C electrocatalyst promoting the polysulfide redox kinetics for Na–S batteries. Adv. Mater. **34**(14), 2200479 (2022). 10.1002/adma.202200479 10.1002/adma.202200479 35142394

[54] P. Wang, Y. Song, Z. Xu, N. Li, J. Sun et al., Hierarchical Mo_x_C@NC hollow microsphere with incorporated Mo vacancies as multifunctional confined reactors for high-loading Li–S batteries. Inorg. Chem. Front. **9**(10), 2194–2203 (2022). 10.1039/d1qi01649k

[55] Z. Cui, Y. Li, G. Fu, X. Li, J.B. Goodenough, Robust Fe_3_Mo_3_C supported IrMn clusters as highly efficient bifunctional air electrode for metal–air battery. Adv. Mater. **29**(40), 1702385 (2017). 10.1002/adma.201702385 10.1002/adma.201702385 28856742

[56] J. Yu, K. Wang, Vacancy formation and clustering behavior in δ-MoN: a systematic density functional theory study. Nanomaterials **15**(11), 810 (2025). 10.3390/nano15110810 40497858 10.3390/nano15110810 PMC12157999

[57] G. Chen, C. He, G. Yan, H. Gu, X. Wu et al., Mastering vacancy engineering for electrocatalysis: insights into classification, synthesis, and characterization. Nano Mater. Sci. (2025). 10.1016/j.nanoms.2025.05.005

[58] F. Li, X. Zhao, J. Mahmood, M.S. Okyay, S.-M. Jung et al., Macroporous inverse opal-like Mo_*x*_C with incorporated Mo vacancies for significantly enhanced hydrogen evolution. ACS Nano **11**(7), 7527–7533 (2017). 10.1021/acsnano.7b04205 28692795 10.1021/acsnano.7b04205

[59] Y. Ma, J. Liu, M. Chen, Q. Yang, H. Chen et al., Selective hydrogenation of naphthalene to decalin over surface-engineered α-MoC based on synergy between Pd doping and Mo vacancy generation. Adv. Funct. Mater. **32**(25), 2112435 (2022). 10.1002/adfm.202112435

[60] H. Cheng, P. Cui, F. Wang, L.-X. Ding, H. Wang, High efficiency electrochemical nitrogen fixation achieved with a lower pressure reaction system by changing the chemical equilibrium. Angew. Chem. Int. Ed. **58**(43), 15541–15547 (2019). 10.1002/anie.201910658 10.1002/anie.201910658 31502747

[61] R.Y. Hu, L.Y. Liu, J.H. He, Y. Zhou, S.B. Wu et al., Preparation and electrochemical properties of bimetallic carbide Fe_3_Mo_3_C/Mo_2_C@carbon nanotubes as negative electrode material for supercapacitor. J. Energy Storage **72**, 108656 (2023). 10.1016/j.est.2023.108656

[62] J.-B. Luo, X.-Z. Wang, J. Zhang, Y. Zhou, Fe-doped Co_3_O_4_ anchored on hollow carbon nanocages for efficient electrocatalytic oxygen evolution. J. Fuel Chem. Technol. **51**(5), 571–579 (2023). 10.1016/s1872-5813(22)60080-x

[63] W. Huang, H. Liang, Z. Su, L. Liu, Y. Song et al., Molten-salt-assisted industrial-scale Fe_3_C-based nano-reactors endow dynamic regulation from polysulfides guided transfer to enforced transformation mechanism. Adv. Funct. Mater. e22858 (2025). 10.1002/adfm.202522858

[64] S. Ning, X. Wu, H. Song, X. Ma, S. Yue et al., Light-field orchestrated tandem photothermal catalysis for highly selective CO_2_-to-C_2+_ olefin conversion. J. Am. Chem. Soc. **148**(1), 1728–1740 (2026). 10.1021/jacs.5c18890 41431122 10.1021/jacs.5c18890

[65] F. Qian, M. Wang, Z. Wei, Y. Cai, Z. Sun et al., Stabilized Fe_7_C_3_ catalyst with K–Mg dual promotion for robust CO_2_ hydrogenation to high-value olefins. Nat. Commun. **16**, 8044 (2025). 10.1038/s41467-025-63218-3 40877312 10.1038/s41467-025-63218-3 PMC12394697

[66] C. Liu, R. Yang, J. Wang, B. Liu, X. Chang et al., Synergistic catalysts with Fe single atoms and Fe_3_C clusters for accelerated oxygen adsorption kinetics in oxygen reduction reaction. Angew. Chem. Int. Ed. **64**(21), e202501266 (2025). 10.1002/anie.202501266 10.1002/anie.202501266 40065733

[67] S. Sun, Z. Liu, F. Yang, T. Qiu, M. Wang et al., Fe C enhancing the catalytic activity of FeN in oxidative dehydration of N-heterocycles. Green Chemical Engineering **3**(4), 349–358 (2022). 10.1016/j.gce.2021.12.007

[68] A. Mehmood, M. Gong, F. Jaouen, A. Roy, A. Zitolo et al., High loading of single atomic iron sites in Fe–NC oxygen reduction catalysts for proton exchange membrane fuel cells. Nat. Catal. **5**(4), 311–323 (2022). 10.1038/s41929-022-00772-9

[69] H. Wang, X. Qin, Z. Gao, Y. Diao, S. Liu et al., Ni redispersion from SiO_2_ to molybdenum carbide creates dual interfaces to boost tandem CO_2_ hydrogenation. Nat. Commun. **16**, 11383 (2025). 10.1038/s41467-025-64030-9 41429769 10.1038/s41467-025-64030-9 PMC12738546

[70] X. Liu, L. Zhao, H. Xu, Q. Huang, Y. Wang et al., Tunable cationic vacancies of cobalt oxides for efficient electrocatalysis in Li–O_2_ batteries. Adv. Energy Mater. **10**(40), 2001415 (2020). 10.1002/aenm.202001415

[71] Y. Sun, K. Lai, X. Shi, N. Li, Y. Gao et al., Regulating metal cation Cu vacancies on ZnIn_2_S_4_/Cu_1.81_S to achieve high selectivity for the photocatalytic reduction of CO_2_ to CH_4_. Applied Catalysis B: Environment and Energy **365**, 124907 (2025). 10.1016/j.apcatb.2024.124907

[72] J. Ge, Y. Chen, Y. Zhao, Y. Wang, F. Zhang et al., Activated MoS_2_ by constructing single atomic cation vacancies for accelerated hydrogen evolution reaction. ACS Appl. Mater. Interfaces **14**(23), 26846–26857 (2022). 10.1021/acsami.2c06708 35657022 10.1021/acsami.2c06708

[73] N. Österbacka, H. Ouhbi, F. Ambrosio, J. Wiktor, Spontaneous oxygen vacancy ionization enhances water oxidation on BiVO_4_. ACS Energy Lett. **9**(1), 153–158 (2024). 10.1021/acsenergylett.3c02348

[74] L. Meng, J. He, W. Tian, M. Wang, R. Long et al., Ni/Fe codoped In_2_S_3_ nanosheet arrays boost photo-electrochemical performance of planar Si photocathodes. Adv. Energy Mater. **9**(38), 1902135 (2019). 10.1002/aenm.201902135

[75] Y.-C. Wang, C.-Y. Chang, T.-F. Yeh, Y.-L. Lee, H. Teng, Formation of internal p–n junctions in Ta_3_N_5_ photoanodes for water splitting. J. Mater. Chem. A **2**(48), 20570–20577 (2014). 10.1039/c4ta04501g

[76] D. Vidyasagar, S.G. Ghugal, S.S. Umare, M. Banavoth, Extended π-conjugative n-p type homostructural graphitic carbon nitride for photodegradation and charge-storage applications. Sci. Rep. **9**, 7186 (2019). 10.1038/s41598-019-43312-5 31076639 10.1038/s41598-019-43312-5 PMC6510722

[77] Y. Xiao, C. Feng, J. Fu, F. Wang, C. Li et al., Band structure engineering and defect control of Ta_3_N_5_ for efficient photoelectrochemical water oxidation. Nat. Catal. **3**(11), 932–940 (2020). 10.1038/s41929-020-00522-9

[78] Y. Yang, S. Wang, Y. Jiao, Z. Wang, M. Xiao et al., An unusual red carbon nitride to boost the photoelectrochemical performance of wide bandgap photoanodes. Adv. Funct. Mater. **28**(47), 1805698 (2018). 10.1002/adfm.201805698

[79] X. Wang, X. Liu, Y. Wu, Y. Fu, H. Zhang et al., Thinnest npn homojunction for inspired photoelectrochemical water splitting. Appl. Catal. B Environ. **323**, 122182 (2023). 10.1016/j.apcatb.2022.122182

[80] Y. Wu, X. Liu, H. Zhang, J. Li, M. Zhou et al., Atomic sandwiched p-n homojunctions. Angew. Chem. Int. Ed. **60**(7), 3487–3492 (2021). 10.1002/anie.202012734 10.1002/anie.202012734 33128336

[81] Y. Ning, Z. Zhang, F. Teng, X. Fang, Novel transparent and self-powered UV photodetector based on crossed ZnO nanofiber array homojunction. Small **14**(13), 1703754 (2018). 10.1002/smll.201703754 10.1002/smll.201703754 29383872

[82] Q. Ma, Q. Liu, Z. Li, J. Pu, J. Mujtaba et al., Oxygen vacancy-mediated amorphous GeOx assisted polysulfide redox kinetics for room-temperature sodium-sulfur batteries. J. Colloid Interface Sci. **629**, 76–86 (2023). 10.1016/j.jcis.2022.09.071 36152582 10.1016/j.jcis.2022.09.071

[83] J. Zhou, Y. Yang, Y. Zhang, S. Duan, X. Zhou et al., Sulfur in amorphous silica for an advanced room-temperature sodium–sulfur battery. Angew. Chem. Int. Ed. **60**(18), 10129–10136 (2021). 10.1002/anie.202015932 10.1002/anie.202015932 33554433

[84] F. Xiao, H. Wang, T. Yao, X. Zhao, X. Yang et al., MOF-derived CoS_2_/N-doped carbon composite to induce short-chain sulfur molecule generation for enhanced sodium–sulfur battery performance. ACS Appl. Mater. Interfaces **13**(15), 18010–18020 (2021). 10.1021/acsami.1c02301 33822575 10.1021/acsami.1c02301

[85] J. Wu, Z. Yu, Y. Yao, L. Wang, Y. Wu et al., Bifunctional catalyst for liquid–solid redox conversion in room-temperature sodium–sulfur batteries. Small Struct. **3**(8), 2200020 (2022). 10.1002/sstr.202200020

[86] Y. Qi, Q.-J. Li, Y. Wu, S.-J. Bao, C. Li et al., A Fe_3_N/carbon composite electrocatalyst for effective polysulfides regulation in room-temperature Na-S batteries. Nat. Commun. **12**, 6347 (2021). 10.1038/s41467-021-26631-y 34732738 10.1038/s41467-021-26631-y PMC8566531

[87] D. Fang, S. Huang, T. Xu, P. Sun, X.L. Li et al., Low-coordinated Zn–N_2_ sites as bidirectional atomic catalysis for room-temperature Na–S batteries. ACS Appl. Mater. Interfaces **15**(22), 26650–26659 (2023). 10.1021/acsami.3c02599 37226049 10.1021/acsami.3c02599

[88] D. Yang, M. Li, X. Zheng, X. Han, C. Zhang et al., Phase engineering of defective copper selenide toward robust lithium–sulfur batteries. ACS Nano **16**(7), 11102–11114 (2022). 10.1021/acsnano.2c03788 35758405 10.1021/acsnano.2c03788

[89] Z. Zheng, L. Yu, M. Gao, X. Chen, W. Zhou et al., Boosting hydrogen evolution on MoS_2_*via* co-confining selenium in surface and cobalt in inner layer. Nat. Commun. **11**, 3315 (2020). 10.1038/s41467-020-17199-0 32620781 10.1038/s41467-020-17199-0 PMC7334232

[90] Y. Liu, A. Chatterjee, P. Rusch, C. Wu, P. Nan et al., Monodisperse molybdenum nanoparticles as highly efficient electrocatalysts for Li-S batteries. ACS Nano **15**(9), 15047–15056 (2021). 10.1021/acsnano.1c05344 34529415 10.1021/acsnano.1c05344

[91] M. Ali Raza Anjum, H.Y. Jeong, M.H. Lee, H.S. Shin, J.S. Lee, Efficient hydrogen evolution reaction catalysis in alkaline media by all-in-one MoS_2_ with multifunctional active sites. Adv. Mater. **30**(20), 1707105 (2018). 10.1002/adma.201707105 10.1002/adma.201707105 29603427

[92] P. Guo, K. Sun, X. Shang, D. Liu, Y. Wang et al., Nb_2_O_5_/RGO nanocomposite modified separators with robust polysulfide traps and catalytic centers for boosting performance of lithium–sulfur batteries. Small **15**(40), 1902363 (2019). 10.1002/smll.201902363 10.1002/smll.201902363 31419025

[93] H. Pan, C. Wang, M. Qiu, Y. Wang, C. Han et al., Mo doping and electrochemical activation co-induced vanadium composite as high-rate and long-life anode for Ca-ion batteries. Energy Environ. Mater. **7**(5), e12690 (2024). 10.1002/eem2.12690

[94] P. Cheng, L. Shi, W. Li, X. Fang, D. Cao et al., Efficient regulation of polysulfides by MoS_2_/MoO_3_ heterostructures for high-performance Li-S batteries. Small **19**(16), 2206083 (2023). 10.1002/smll.202206083 10.1002/smll.202206083 36683234

[95] N. Wang, Y. Wang, Z. Bai, Z. Fang, X. Zhang et al., High-performance room-temperature sodium–sulfur battery enabled by electrocatalytic sodium polysulfides full conversion. Energy Environ. Sci. **13**(2), 562–570 (2020). 10.1039/c9ee03251g

[96] G. Yao, Z. Li, Y. Zhang, Y. Xiao, L. Wei et al., Highly flexible carbon film implanted with single-atomic Zn–N_2_ moiety for long-life sodium-sulfur batteries. Adv. Funct. Mater. **34**(5), 2214353 (2024). 10.1002/adfm.202214353

[97] D. Fang, T. Ghosh, S. Huang, Y. Wang, J. Qiu et al., Core–shell tandem catalysis coupled with interface engineering for high-performance room-temperature Na–S batteries. Small **19**(41), 2302461 (2023). 10.1002/smll.202302461 10.1002/smll.202302461 37292002

[98] T. Sun, S. Wang, M. Xu, N. Qiao, Q. Zhu et al., High-performance sulfurized polyacrylonitrile cathode by using MXene as a conductive and catalytic binder for room-temperature Na/S batteries. ACS Appl. Mater. Interfaces **16**(8), 10093–10103 (2024). 10.1021/acsami.3c17874 38359415 10.1021/acsami.3c17874

[99] S. Zhang, M. Huang, Y. Wang, Z. Wang, H. Wang et al., Achieving a quasi-solid-state conversion of polysulfides *via* building high efficiency heterostructure for room temperature Na–S batteries. Adv. Energy Mater. **14**(14), 2303925 (2024). 10.1002/aenm.202303925

[100] C. Wu, Y. Lei, L. Simonelli, D. Tonti, A. Black et al., Continuous carbon channels enable full Na-ion accessibility for superior room-temperature Na–S batteries. Adv. Mater. **34**(8), 2108363 (2022). 10.1002/adma.202108363 10.1002/adma.202108363 34881463

[101] D. Ma, Y. Li, J. Yang, H. Mi, S. Luo et al., New strategy for polysulfide protection based on atomic layer deposition of TiO_2_ onto ferroelectric-encapsulated cathode: toward ultrastable free-standing room temperature sodium–sulfur batteries. Adv. Funct. Mater. **28**(11), 1705537 (2018). 10.1002/adfm.201705537

[102] C. Li, J. Yu, H. Li, R. Zhao, Y. Zhou et al., Balancing electronic spin state *via* atomically-dispersed heteronuclear Fe–Co pairs for high-performance sodium–sulfur batteries. J. Am. Chem. Soc. **147**(10), 8250–8259 (2025). 10.1021/jacs.4c15408 40017101 10.1021/jacs.4c15408

[103] W. Song, Z. Wen, X. Wang, K. Qian, T. Zhang et al., Unsaturation degree of Fe single atom site manipulates polysulfide behavior in sodium-sulfur batteries. Nat. Commun. **16**, 2795 (2025). 10.1038/s41467-025-58114-9 40118884 10.1038/s41467-025-58114-9 PMC11928504

[104] D. Zhao, S. Jiang, S. Yu, J. Ren, Z. Zhang et al., Lychee seed-derived microporous carbon for high-performance sodium-sulfur batteries. Carbon **201**, 864–870 (2023). 10.1016/j.carbon.2022.09.075

[105] J. Offermann, S.N. Ul Haq, K.X. Wang, R. Adelung, S.H. Chang et al., Fast-charging lithium–sulfur batteries. Adv. Energy Mater. **15**(26), 2404383 (2025). 10.1002/aenm.202404383

[106] B. Wang, L. Wang, B. Guo, Y. Kong, F. Wang et al., *In situ* electrochemical evolution of amorphous metallic borides enabling long cycling room-/ subzero-temperature sodium-sulfur batteries. Adv. Mater. **36**(48), 2411725 (2024). 10.1002/adma.202411725 10.1002/adma.202411725 39410861

[107] Y. Wang, Y. Wang, C. Xu, Y. Meng, P. Liu et al., Phosphor-doped carbon network electrocatalyst enables accelerated redox kinetics of polysulfides for sodium–sulfur batteries. ACS Nano **18**(4), 3839–3849 (2024). 10.1021/acsnano.3c12754 38227979 10.1021/acsnano.3c12754

[108] X. Ye, J. Ruan, Y. Pang, J. Yang, Y. Liu et al., Enabling a stable room-temperature sodium–sulfur battery cathode by building heterostructures in multichannel carbon fibers. ACS Nano **15**(3), 5639–5648 (2021). 10.1021/acsnano.1c00804 33666431 10.1021/acsnano.1c00804

[109] Y. Zhang, C. Ma, C. Zhang, L. Ma, S. Zhang et al., Selective catalysis of single V atoms and VN_1-__*x*_ nanodots enables fast polysulfides conversion in lithium–sulfur batteries. Chem. Eng. J. **452**, 139410 (2023). 10.1016/j.cej.2022.139410

[110] Z. Shen, M. Cao, Y. Wen, J. Li, X. Zhang et al., Tuning the local coordination of CoP_1–__*x*_S_*x*_ between NiAs- and MnP-type structures to catalyze lithium–sulfur batteries. ACS Nano **17**(3), 3143–3152 (2023). 10.1021/acsnano.2c12436 36715422 10.1021/acsnano.2c12436

[111] B. Qin, Y. Li, Q. Wang, S. Zhang, J. Zhang et al., Zinc-doping-induced electronic states modulation of molybdenum carbide: expediting rate-determining steps of sulfur conversion in lithium-sulfur batteries. Adv. Sci. **12**(22), 2417126 (2025). 10.1002/advs.202417126 10.1002/advs.202417126 PMC12165060 40162579

