# Supplementary material for: Pisiform Homojunction with Energy Band Bending Induced via Co-Implantation Design Enabling Fast-Charging Sodium-Sulfur Battery
Source: Nanomicro Lett. 2026 Mar 27;18:306. doi: 10.1007/s40820-026-02163-2 (PMC13031616; doi:10.1007/s40820-026-02163-2)
Supplement: Supplementary file 1 — Supplementary file1 (DOCX 8694 kb) [file 40820_2026_2163_MOESM1_ESM.docx]

Supporting Information for

**Pisiform Homojunction with Energy Band Bending Induced via Co-Implantation Design Enabling Fast-Charging Sodium-Sulfur Battery**

Yanjun Gao^1^, Zujia Lu^1^, Qiyao Yu^1^*, Jianguo Zhang^1^*

^1^State Key Laboratory of Explosion Science and Safety Protection, Beijing Institute of Technology, Beijing 100081, People’s Republic of China

*Corresponding authors. E-mail: [qiyaoyu@bit.edu.cn](mailto:qiyaoyu@bit.edu.cn) (Qiyao Yu); [zjgbit@bit.edu.cn](mailto:zjgbit@bit.edu.cn) (Jianguo Zhang)

**Supplementary Figures and Tables**


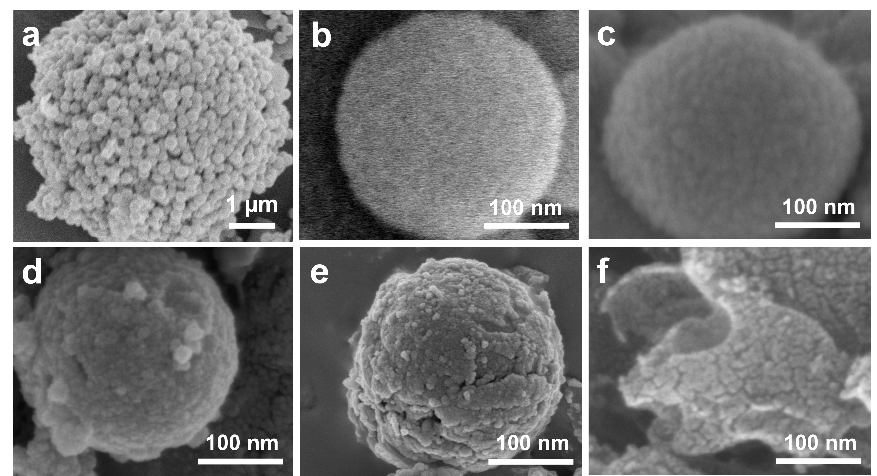


**Fig. S1 a** SEM images of v_6_Fe-Mo_2_C/C. **b** Mo_2_C/C. **c** v_0_Fe-Mo_2_C/C. **d** v_2_Fe-Mo_2_C/C. **e** v_4_Fe-Mo_2_C/C. **f** v_12_Fe-Mo_2_C/C


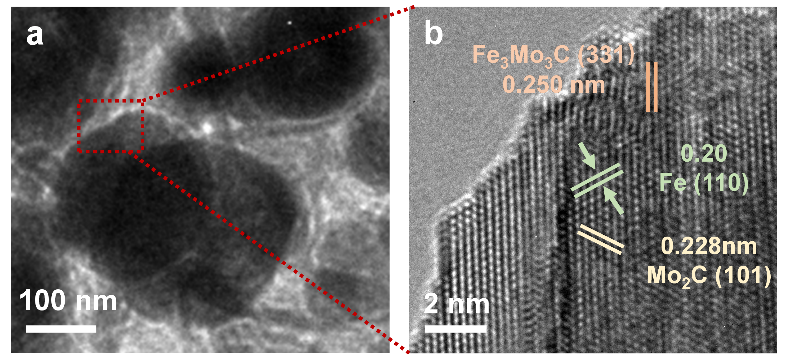


**Fig. S2** **a** TEM image of the v_2_Fe-Mo_2_C/C sample. **b** HR-TEM image of the area corresponding to (**a**)

The lattice spacing of 0.253 nm corresponding to Fe_3_Mo_3_C species ((331) facet), 0.20 nm and 0.228 nm indexed to Fe phase ((110) facet) and Mo_2_C phase ((101) facet), respectively, appear on the v_2_Fe-Mo_2_C/C surface, thereby confirming the coexistence of the three components.


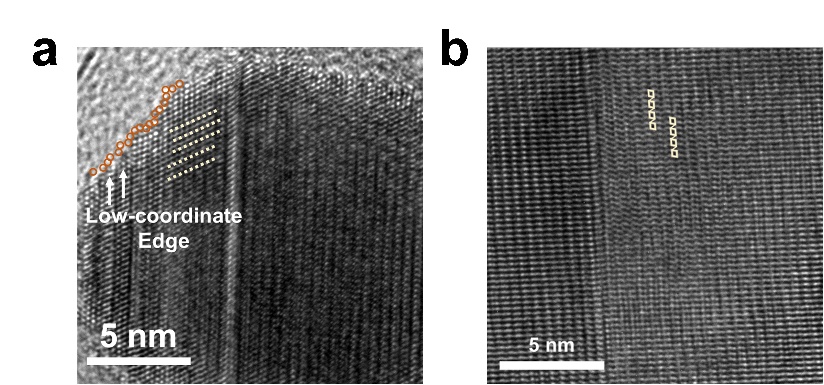


**Fig. S3** HR-TEM images of v_6_Fe-Mo_2_C/C: low-coordinate edge and surface crystal lattice distortion


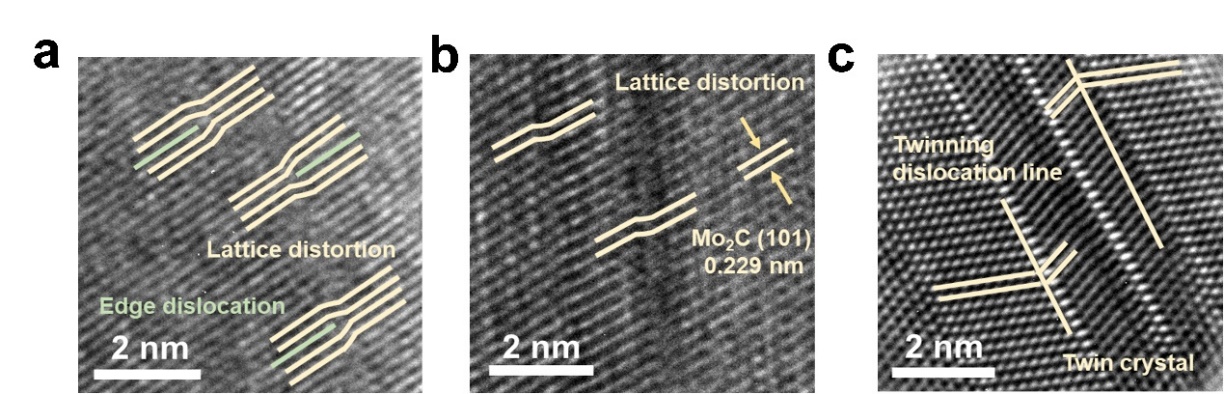


**Fig. S4** HR-TEM images towards surface defects in the v_6_Fe-Mo_2_C/C sample


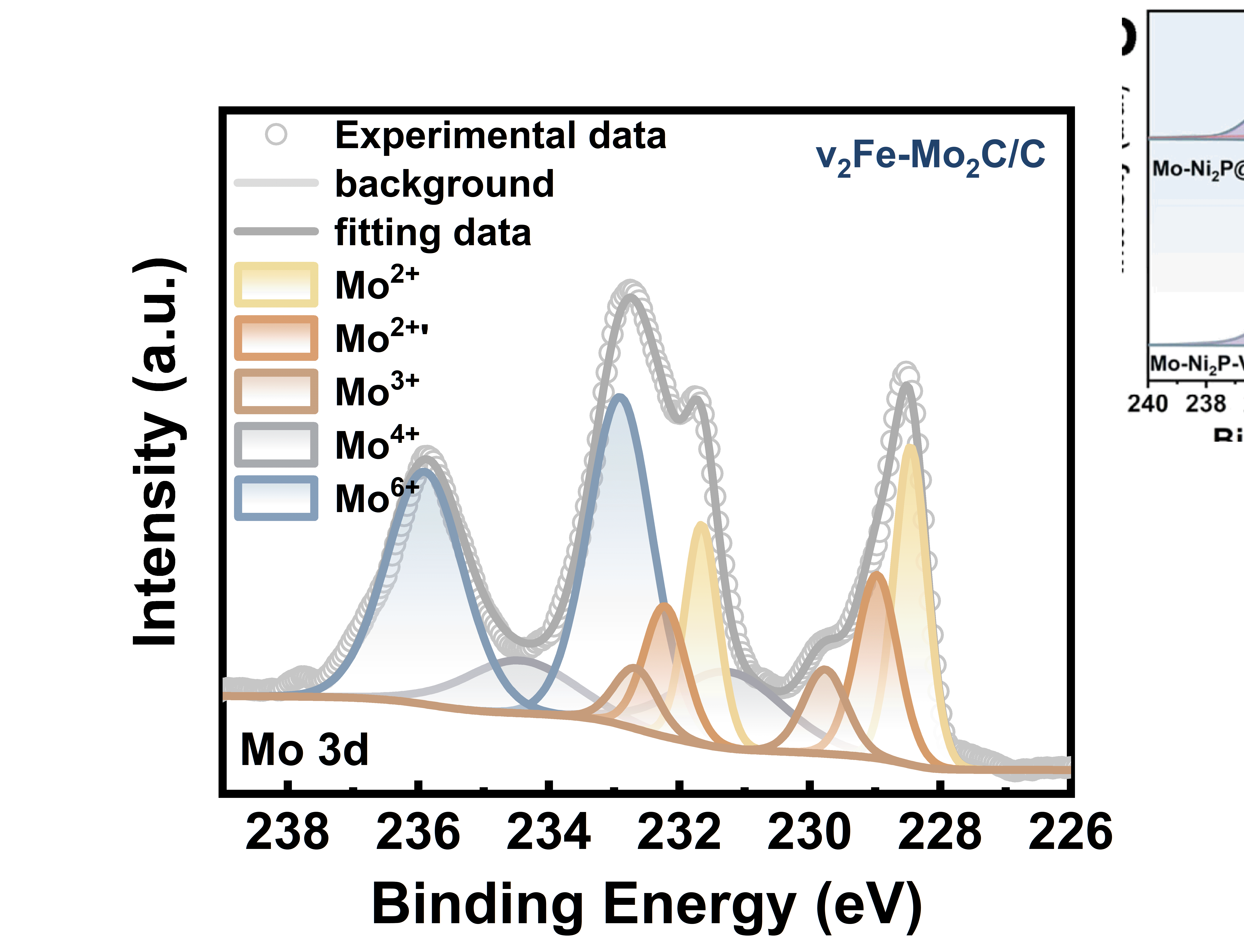


**Fig. S5** High resolution XPS spectra of Mo 3*d* of the v_2_Fe-Mo_2_C/C sample


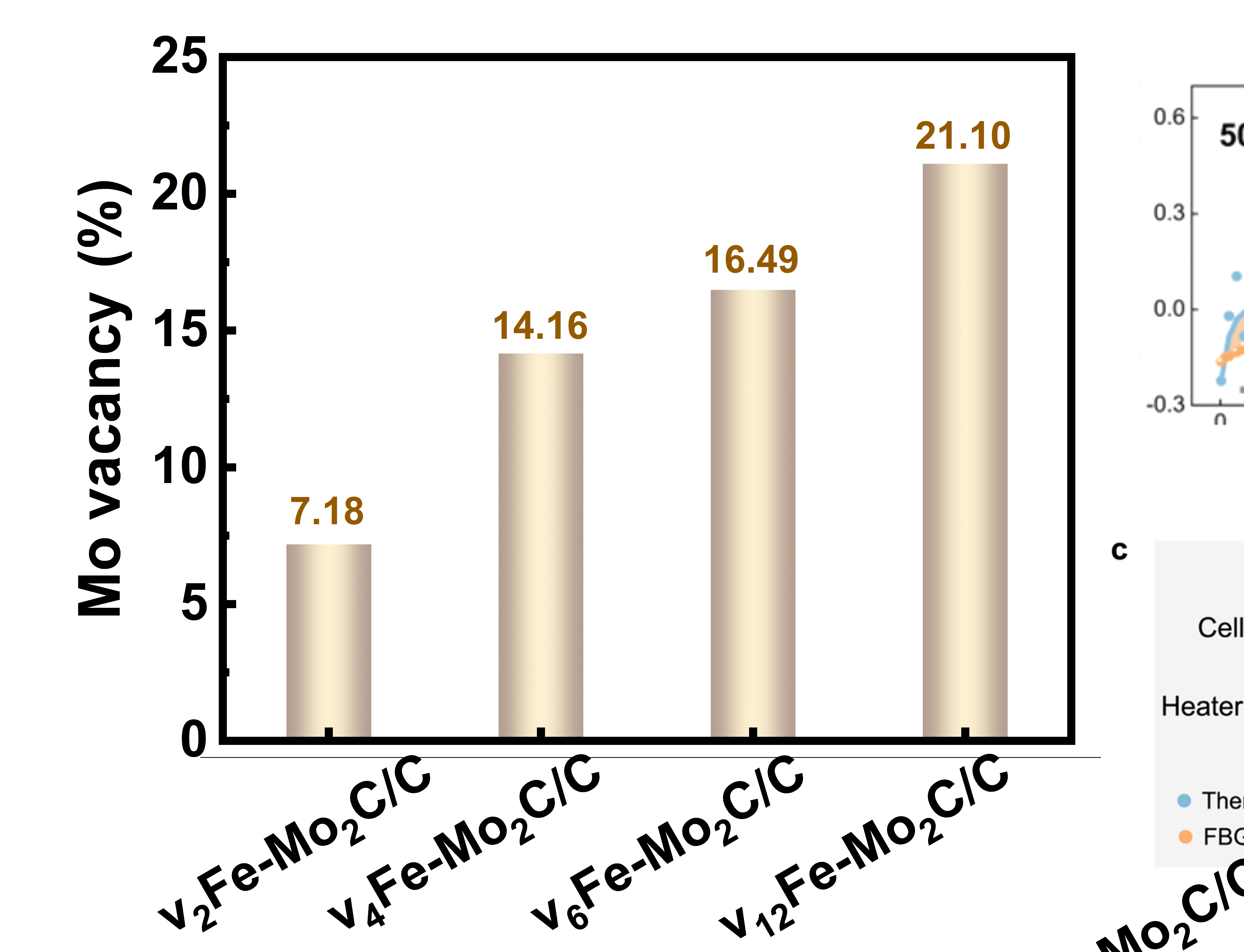


**Fig. S6** Contents of Mo vacancies based on XPS analysis of Mo 3*d*


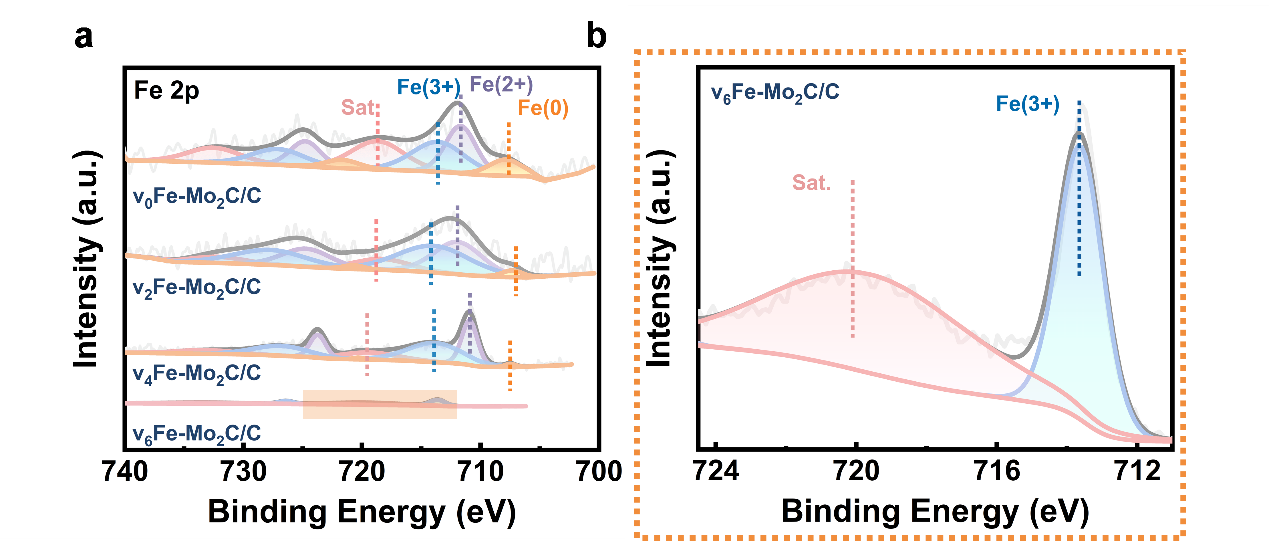


**Fig. S7** **a** High resolution XPS spectra of Fe 2*p* of v_t_Fe-Mo_2_C/C with different HCl etching time. **b** Corresponding zoom-in regions of (**a**) in a binding energy range from 711 to 724 eV


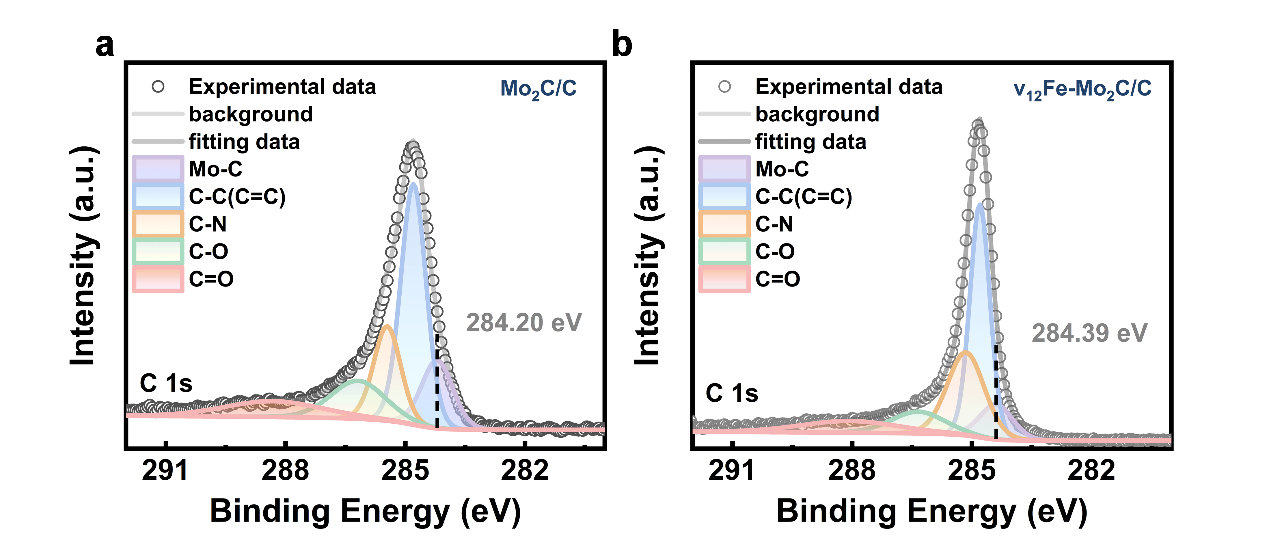


**Fig. S8** **a** High-resolution XPS spectra of C 1*s* in Mo_2_C/C. **b** C 1*s* spectrum of v_12_Fe-Mo_2_C/C


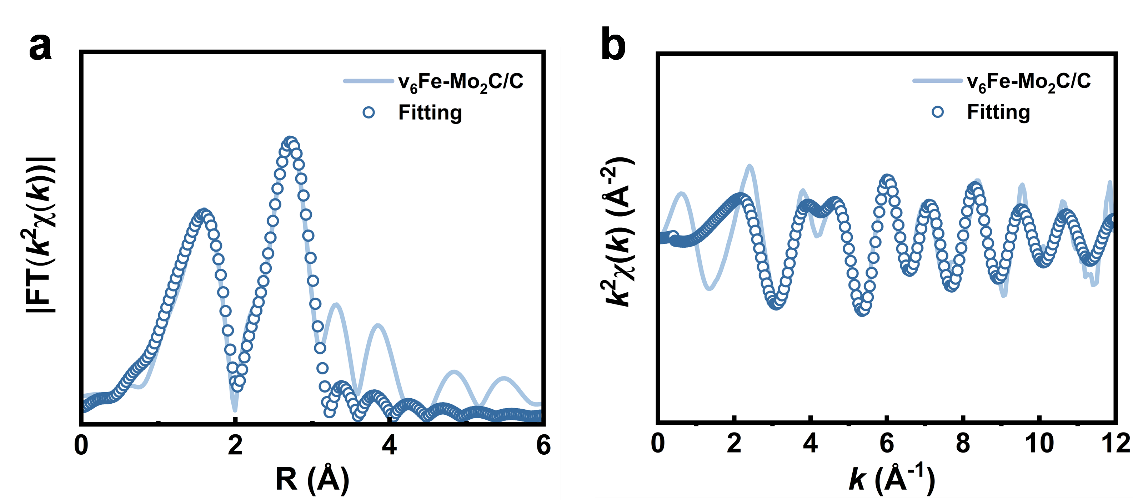


**Fig. S9** EXAFS spectra of v_6_Fe-Mo_2_C/C in (**a**) R-space and (**b**) k-space


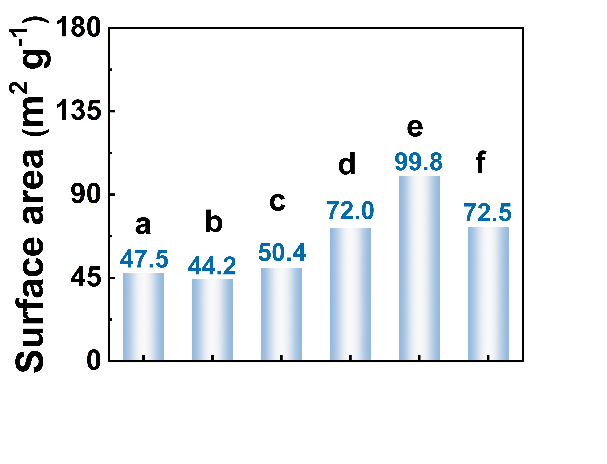


**Fig. S10** Values of BET specific surface area. a-f represent Mo₂C/C, v_0_Fe-Mo₂C/C, v_2_Fe-Mo₂C/C, v_4_Fe-Mo₂C/C, v_6_Fe-Mo₂C/C and v_12_Fe-Mo₂C/C, respectively


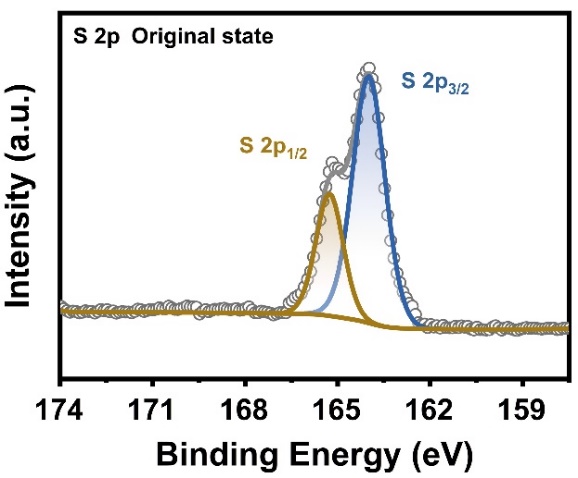


**Fig. S11** High-resolution XPS spectra of S 2*p* for the v_6_Fe-Mo_2_C/C@S


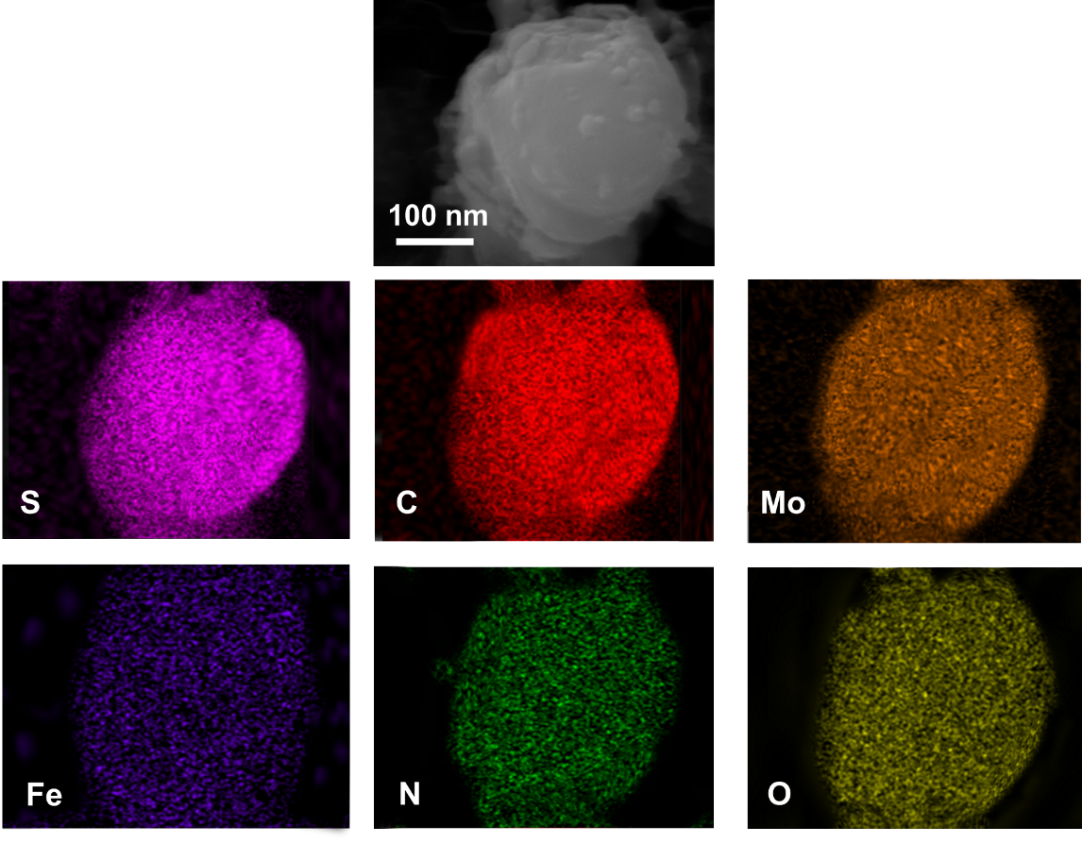


**Fig. S12** EDS elemental mapping of v_6_Fe-Mo_2_C/C@S

Together with the XPS and TGA results, EDS mapping verifies the successful loading of active sulfur, which can be regarded as an essential prerequisite for achieving high reversible capacity and energy density. Meanwhile, EDS also demonstrates the homogeneous distribution of the elements involved in the host.


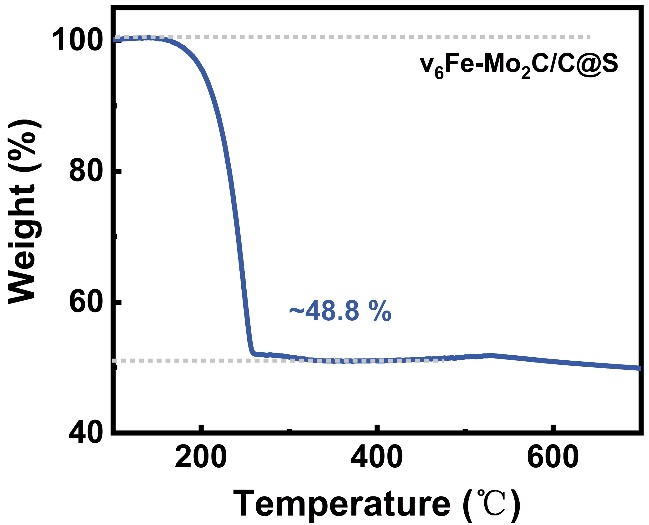


**Fig. S13** TGA curve of v_6_Fe-Mo_2_C/C@S


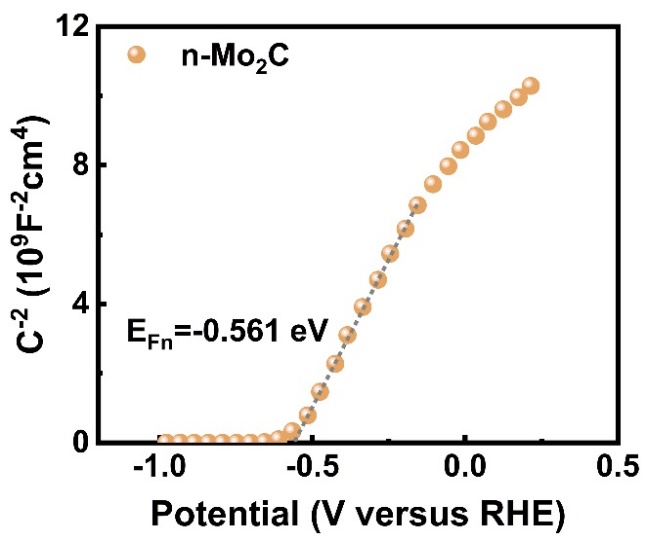


**Fig. S14** Mott-Schottky plot of n-Mo_2_C


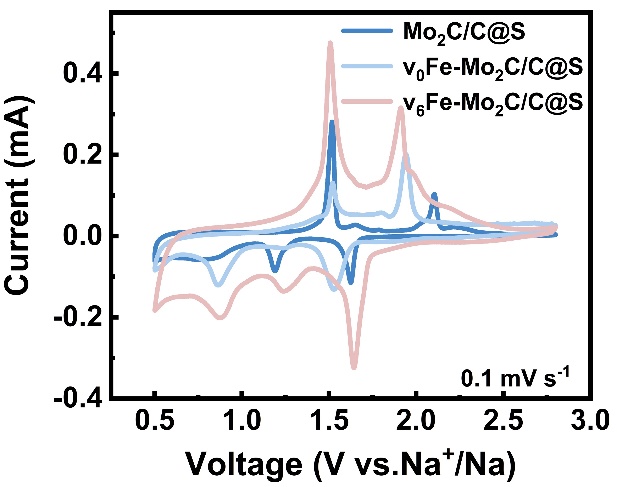


**Fig. S15** CV curves of Mo_2_C/C@S, v_0_Fe-Mo_2_C/C@S and v_6_Fe-Mo_2_C/C@S cathodes at 0.1 mV s^-1^


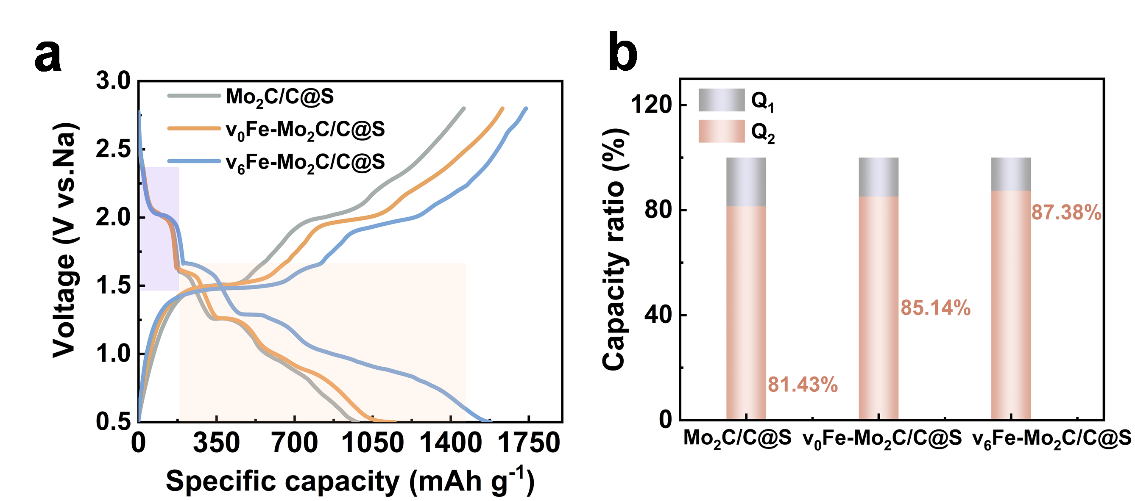


**Fig. S16** **a** Galvanostatic charge/discharge profiles of Mo_2_C/C@S, v_0_Fe-Mo_2_C-0/C@S and v_6_Fe-Mo_2_C/C@S cathodes at 0.1 A g^-1^. **b** Values of Q_1_ and Q_2_ obtained from charge/discharge profiles


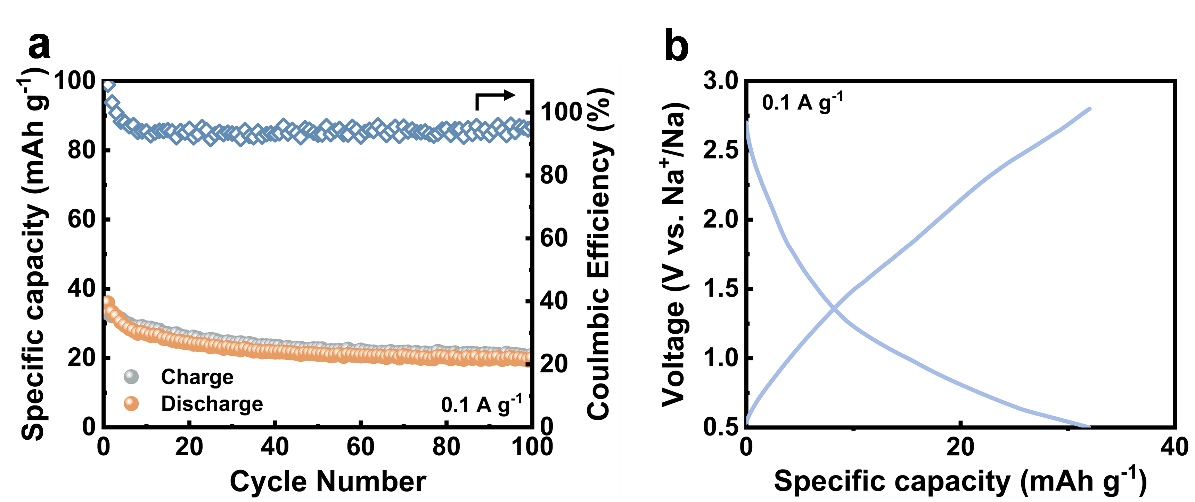


**Fig. S17** **a** Cycling performance of v_6_Fe-Mo_2_C/C host material. **b** GCD curves at 0.1 A g^-1^

Notably, the negligible capacity of pure v_6_Fe-Mo_2_C/C electrode without sulfur clearly confirms that sulfur is the sole active material, and the excellent performance obtained in this work originates from the improvement of sulfur-based energy-storage process by host rather than from the introduction of additional capacity-contributing materials.


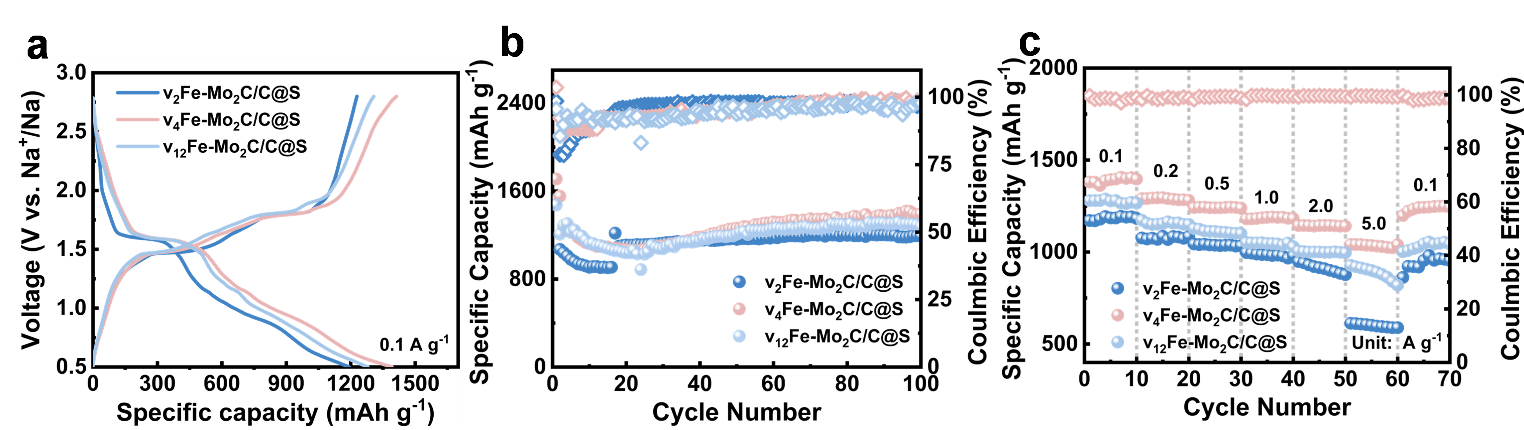


**Fig. S18** Electrochemical performance analysis for v_2_Fe-Mo_2_C/C@S, v_4_Fe-Mo_2_C/C@S and v_12_Fe-Mo_2_C/C@S cathodes. **a** GCD curves at 100^th^ cycle. **b** Cycling properties at 0.1 A g^-1^. **c** Rate properties from 0.1 to 5 A g^-1^


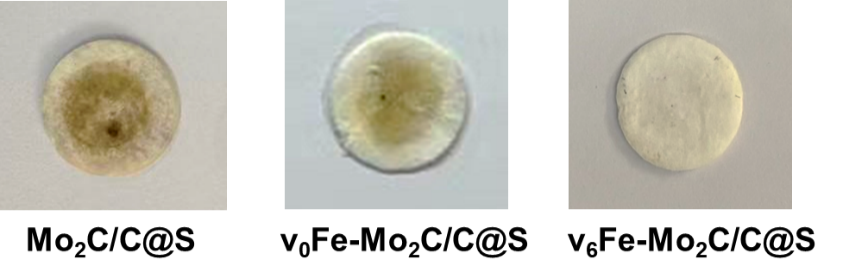


**Fig. S19** Optical images of the separators after disassembling batteries with Mo_2_C/C@S, v_0_Fe-Mo_2_C/C@S and v_6_Fe-Mo_2_C/C@S, respectively


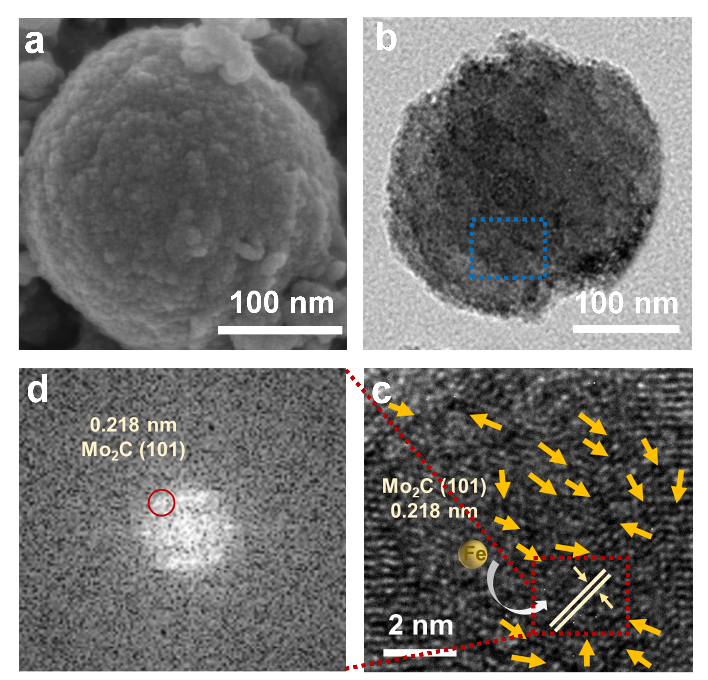


**Fig. S20** Structural characterization of v_6_Fe-Mo_2_C/C@S cathode after 100 cycles: **a** SEM image. **b** TEM image. **c** HR-TEM image corresponding to the circled region in (**b**) highlighting the Mo vacancies and Fe-doping. d FFT pattern in the marked area of (**c**)


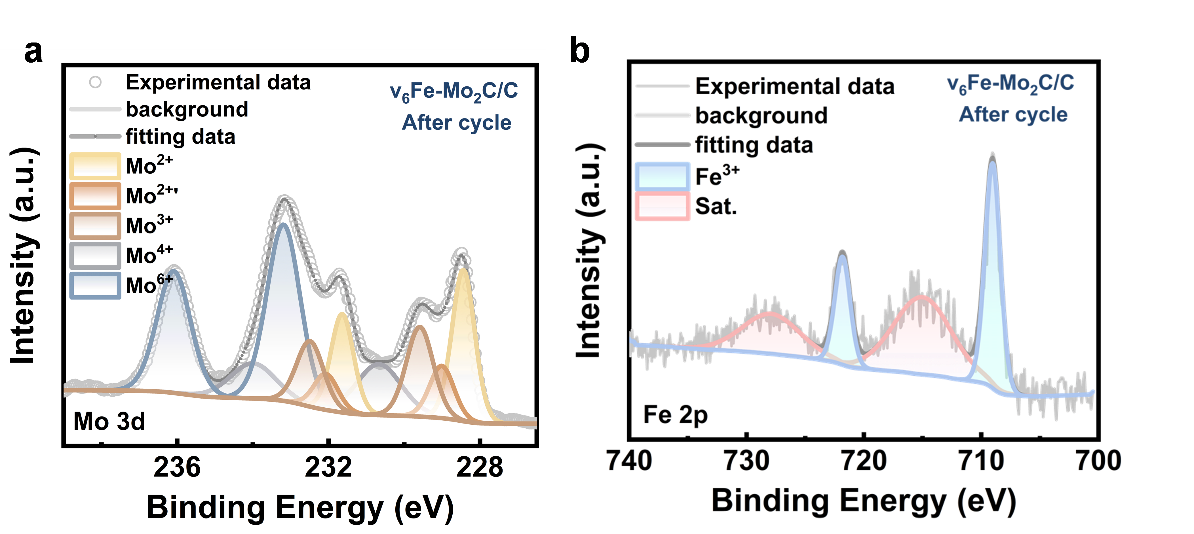


**Fig. S21** Ex situ XPS study toward v_6_Fe-Mo_2_C/C@S cathode after 100 cycles: **a** Mo 3*d* spectrum. **b** Fe 2*p* spectrum at the fully charge states


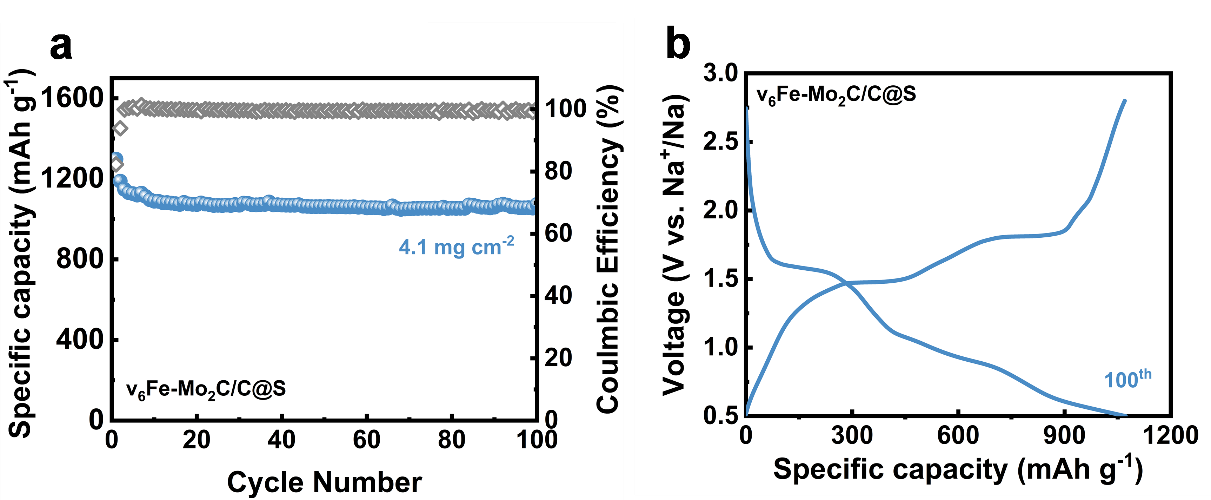


**Fig. S22** **a** Cycling performance of v_6_Fe-Mo_2_C/C@S with a high mass loading of 4.1 mg cm^-2^. **b** The corresponding GCD profiles at 0.2 A g^-1^


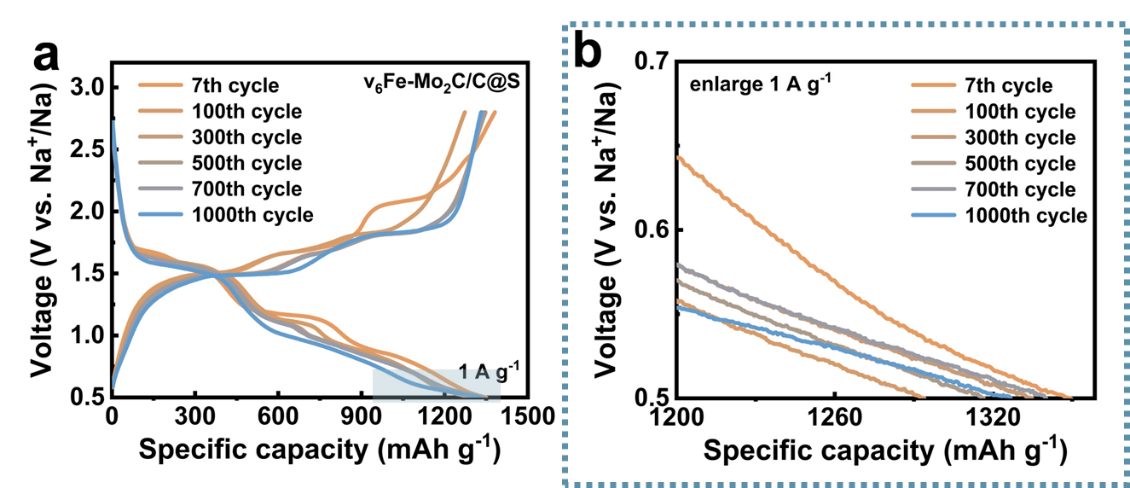


**Fig. S23** GCD profiles at different stages (The blue region in left indicates the area shown in the zoomed-in image in the right)


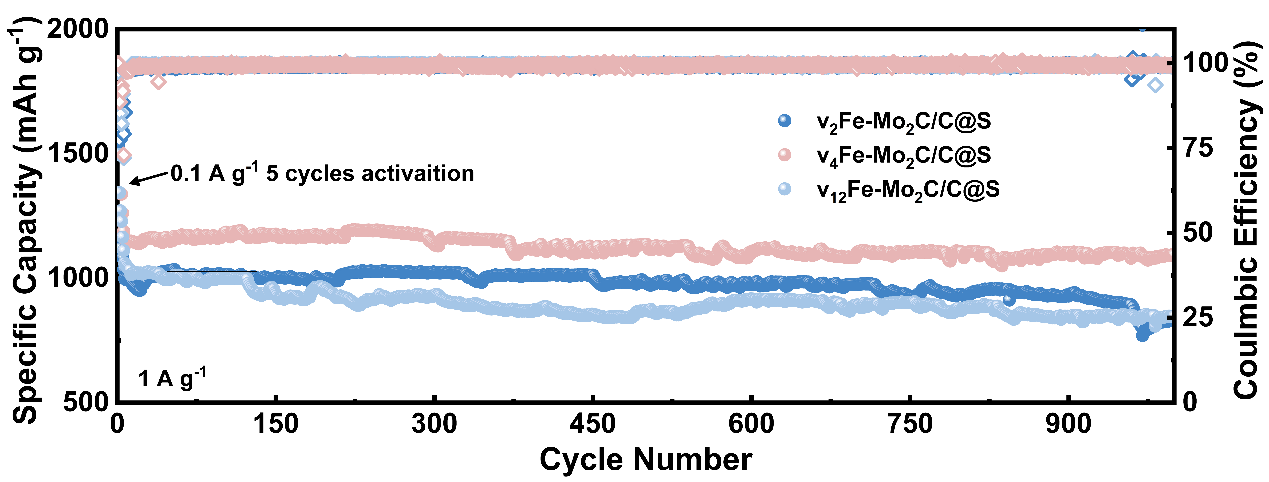


**Fig. S24** Long-term cycling performance at 1 A g^-1^ for Na-S batteries with v_2_Fe-Mo_2_C/C@S, v_4_Fe-Mo_2_C/C@S and v_12_Fe-Mo_2_C/C@S cathodes

Specifically, the v_0_Fe-Mo₂C/C sample was obtained by a Fe-doping strategy, which leads to the performance improvement compared with pristine Mo₂C. The acid treatment for the v_0_Fe-Mo₂C/C sample was carried out and then the etching-time gradually varied to regulate the contents of Fe dopants and Mo vacancies. Based on the above analysis, the samples can be divided into several types: (i) v_0_Fe-Mo_2_C/C represents the sample only with Fe-doping; (ii) v_2_Fe-Mo_2_C/C, v_4_Fe-Mo_2_C/C and v_6_Fe-Mo_2_C/C represent the sample with Fe-doping and Mo vacancies simultaneously, and the differences among the three samples lie in the different ratio of Fe dopant to Mo vacancies; (iii) v_12_Fe-Mo_2_C/C represents the sample only with Mo vacancies. Through rigorous comparison in Figs. 4b, c and S18, at a low current density of 0.1 A g^-1^, the v_6_Fe-Mo₂C/C@S cathode maintains an exceptional capacity of 1508.0 mAh g⁻¹ after 100 cycles, whereas v_4_Fe-Mo₂C/C@S delivers 1392.3 mAh g⁻¹ under the same conditions. In contrast, v_12_Fe-Mo₂C/C@S exhibits a substantially lower capacity of only 1279.9 mAh g⁻¹. A similar trend is observed during a long-term cycling process at 1 A g^-1^ (Figs. 4g and S24). After 1000 cycles, v_6_Fe-Mo₂C/C@S retains 94.2% of its initial capacity, while the capacity retention of v_12_Fe-Mo₂C/C@S decreases markedly with only 76.4% capacity retention, highlighting a pronounced performance disparity. More importantly, rate performance analysis (Figs. 4e and S18c) shows that v_6_Fe-Mo₂C/C@S delivers the specific capacity up to 1337.0 mAh g⁻¹ at 1 A g^-1^, corresponding to approximately 88.8% of its initial capacity at 0.1 A g^-1^. Under the same background, v_4_Fe-Mo₂C/C@S retains 1185.6 mAh g⁻¹ (85.0%), whereas v_12_Fe-Mo₂C/C@S reaches only 1047.1 mAh g⁻¹, with a capacity retention of 79.8%. Upon reverting from high current density back to 0.1 A g^-1^, v_6_Fe-Mo₂C/C@S recovers to 94.9% (1429.3 mAh g⁻¹) of its initial capacity, in sharp contrast to v_12_Fe-Mo₂C/C@S recovering only 81.8% (1044.6 mAh g⁻¹). Across all evaluated metrics, including the specific capacity, rate and cycling capability, the v_6_Fe-Mo₂C/C@S cathode exhibits the best electrochemical performance, followed by v_4_Fe-Mo₂C/C@S. In contrast, v_12_Fe-Mo₂C/C@S delivers the markedly inferior performance. Therefore, an appropriate ratio of Fe dopant to Mo vacancies in Mo₂C maximizes the effect of the induced homojunction, thereby endowing it with the optimal electrochemical capability.

Moreover, based on a thorough review of the relevant background and prior literature, the targeted evaluations and analyses of “fast-charging performance” were carried out, specifically employing an asymmetric testing protocol, as detailed below:

For commercially deployed lithium-ion batteries (LIBs) and their applications in the electric vehicles (EVs) sector, cost and driving range have become broadly comparable to those of internal combustion engine vehicles (ICEVs) [S1]. However, a critical challenge still constrains their widespread adoption: whether charging can be achieved as rapidly and conveniently as refueling, known as extreme fast charging (XFC). Zhang et al.[S2] compared the charging time required for the fastest-charging mass-produced EVs on the market to charge from 10% to 80% state, revealing that charging durations have steadily decreased over development and are now approaching the ultimate goal of XFC. With the rapid expansion of portable electronic devices and EVs applications, reducing charging time and improving user experience have made fast-charging capability become an essential requirement for the current development of LIBs.

However, lithium-sulfur (Li-S) batteries, whose commercialization remains far behind that of LIBs, suffer from severe capacity degradation under fast-charging conditions, which has emerged as a critical bottleneck, letting alone sodium-sulfur (Na-S) batteries that are still confined at the laboratory stage. To date, some researches have been devoted to improving the so-called “fast-charging” performance of Li-S batteries. Pang’s group [S3] proposed an all-solid-state Li-S battery (ASSLSB) achieved with lithium thioborophosphate iodide (LBPSI) solid electrolytes and evaluated the fast-charging performance by charging the cell at various C rates and with a constant discharging rate of 1 C. Finally, the presented high specific capacity of 1,497 mAh g^-1^ on charging at 2 C and the still maintained 784 mAh g^-1^ at 20 C strongly demonstrate the ultrafast charging capability. Likewise, wang et al. [S4] utilize a protocol with a constant discharge rate (0.5 C) while increased charge rate from 0.5 C to 4 C to exhibit the attractive fast-charging ability of sulfurized pyrolyzed poly(acrylonitrile) cathode in Li-S batteries. The distinct discharge plateaus at high-rate extremes (18.0 C) and 90.8% capacity recovery upon returning to 2.0 C were achieved based on the S/Cu−PHEO electrode in the rate-capacity test under progressively increasing charge rates (2.0-18.0 C) while maintaining a constant discharge rate of 2.0 C (Fig. 3a) [S5]. So far, the primary obstacles for Na-S batteries still lie in their poor electronic conductivity and sluggish reaction kinetics, which results in unsatisfactory rate performance, not to mention their inability to replenish depleted energy within a short time, as required for the so-called “fast charging” capability. Accordingly, optimizing the redox kinetics and rate properties, particularly the ability to enable fast charging in Na-S batteries, are highly desirable [S6]. As demonstrated by the above literature, the asymmetric testing protocol under the gradually increased charge rates while maintaining a constant discharge rate is necessary, where the rate capability, expressed as the specific capacity retained even at high charge current, can be regarded as the key indicator for assessing the fast-charging performance [S7]. Therefore, the corresponding tests are also carried out in this work (Figs. 4i-k).


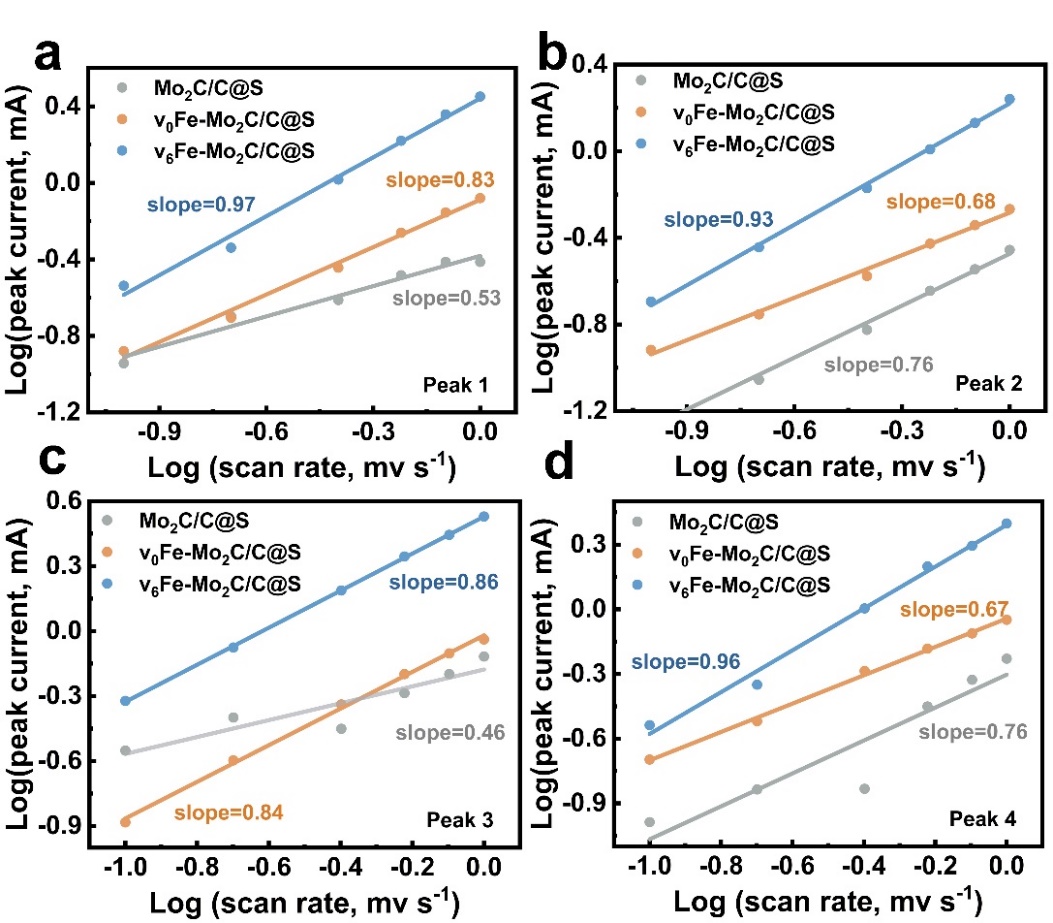


**Fig. S25** Linear fitting between log i and log ν for Mo_2_C/C@S, v_0_Fe-Mo_2_C/C@S, v_6_Fe-Mo_2_C/C@S cathodes of: **a** Peak 1. **b** Peak 2. **c** Peak 3. **d** Peak 4


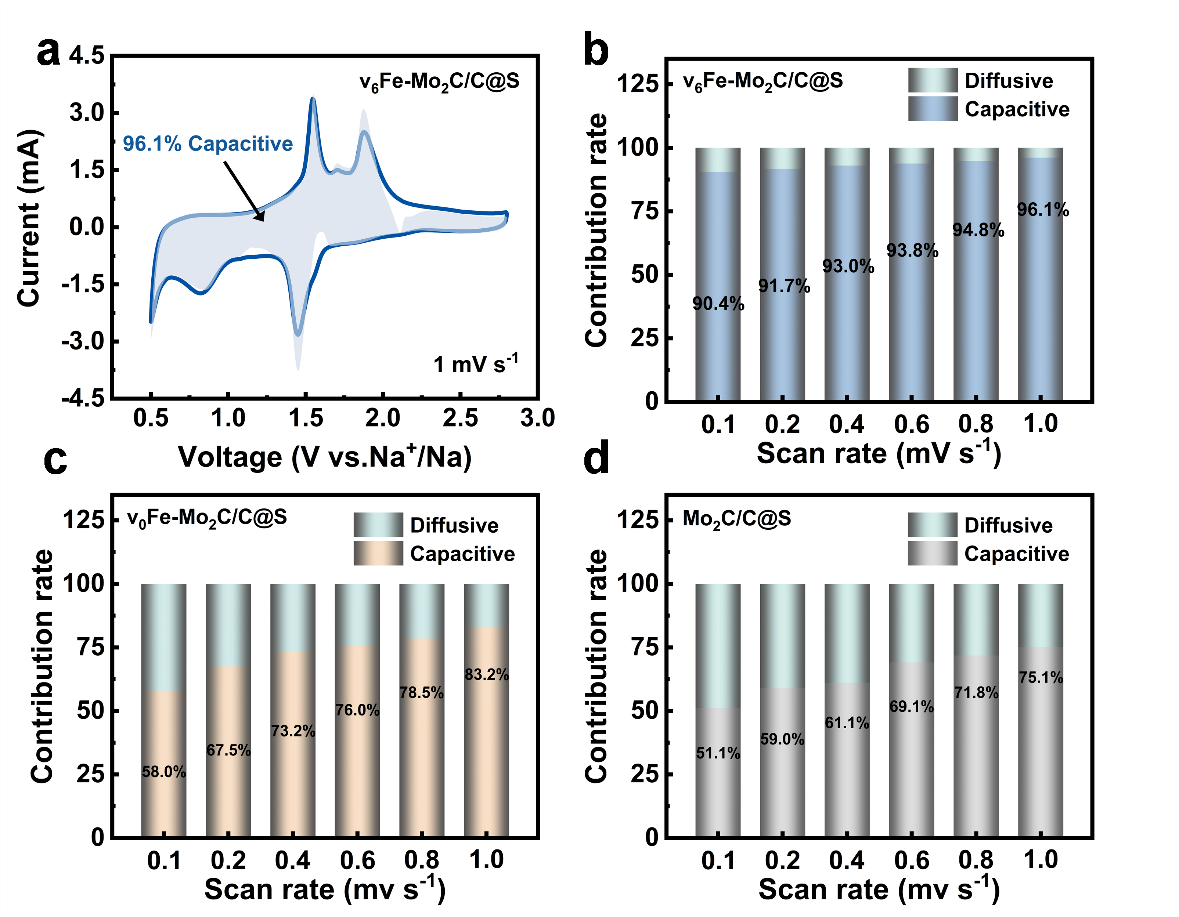


**Fig. S26** **a** Capacitive and diffusion-controlled contribution of v_6_Fe-Mo_2_C/C@S cathode. The capacitive contribution proportion at various scan rates of: **b** v_6_Fe-Mo_2_C/C@S cathode. **c** v_0_Fe-Mo_2_C/C@S cathode. **d** Mo_2_C/C@S cathode


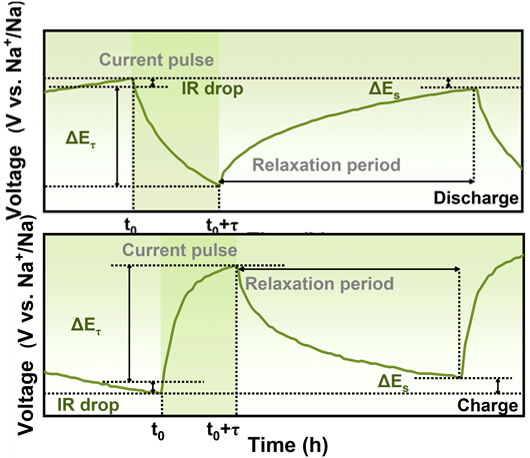


**Fig. S27** Schematic diagrams of the single-step GITT curves


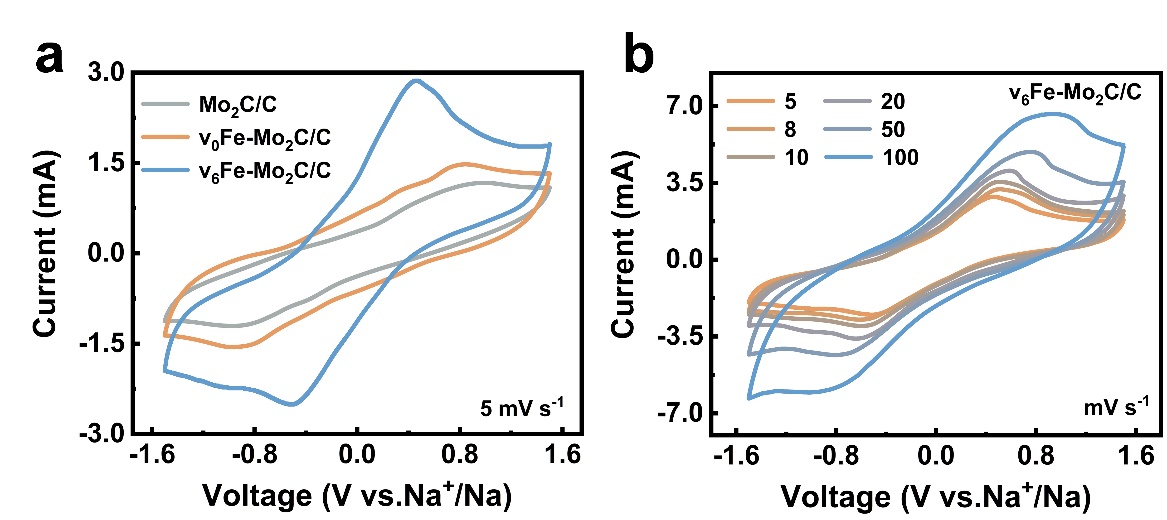


**Fig. S28** **a** CV curves towards the Na_2_S_6_ symmetrical batteries of Mo_2_C/C, v_0_Fe-Mo_2_C/C and v_6_Fe-Mo_2_C/C electrodes. **b** The v_6_Fe-Mo_2_C/C cathode at different scan rate from 5 to 100 mV s^-1^


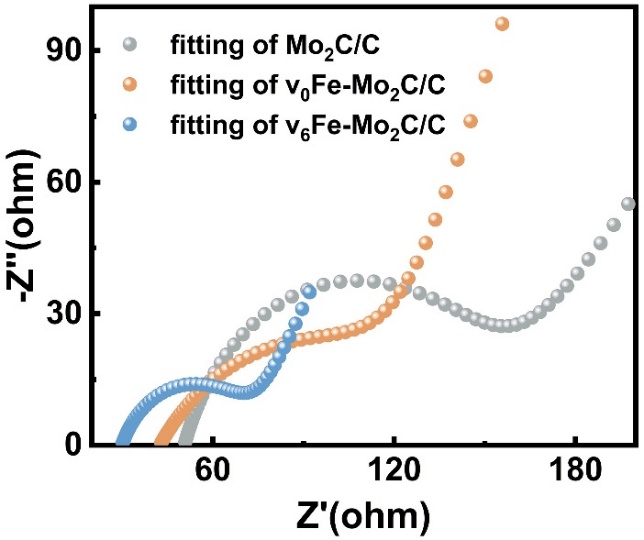


**Fig. S29** The fitting Nyquist plots of the Na_2_S_6_ symmetrical batteries with Mo_2_C/C, v_0_Fe-Mo_2_C/C and v_6_Fe-Mo_2_C/C electrodes


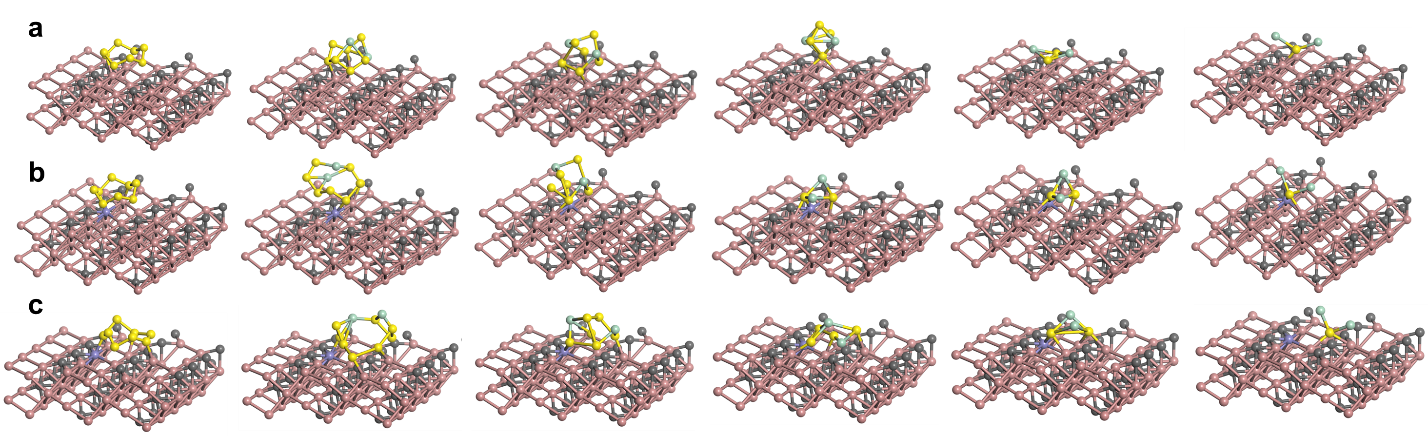


**Fig. S30** The optimized adsorption conformations of sulfur species on: **a** Pristine Mo_2_C. **b** Fe-Mo_2_C. **c** vFe-Mo_2_C-1 with a higher relative ratio of Mo-vacancy to Fe dopant than vFe-Mo_2_C host. The corresponding E_ads_ toward S_8_, Na_2_S_8_, Na_2_S_6_, Na_2_S_4_, Na_2_S_2_ and Na_2_S on vFe-Mo_2_C-1 are calculated to be -1.69, -2.91, -4.75, -3.58, -4.35 and -4.65 eV, respectively

The interaction strength between an electrocatalyst and reactants is a key factor governing catalytic efficiency. According to the Sabatier principle, the interaction between a catalyst and reactive species must be moderate, where this concept is intuitively illustrated by the volcano-curve dependence. If the adsorption capability is too weak, reactant molecules cannot be effectively anchored at the active sites of the catalyst surface, rendering the reaction difficult to initiate. Conversely, excessively strong adsorption hampers subsequent bond cleavage and product desorption, thereby impeding the catalytic cycling. Consequently, optimal catalytic activity is achieved when the adsorption and desorption of reaction intermediates reach a dynamic balance [S8-S10].

In this work, theoretical models of pristine Mo_2_C, Fe-doped Mo_2_C and Mo_2_C jointly decorated by vacancies and Fe-atom were established, denoted as Mo_2_C, Fe-Mo_2_C and vFe-Mo_2_C, respectively. In comparison, vFe-Mo_2_C presents the most favorable binding process for S_8_, Na_2_S_8_, Na_2_S_6_, Na_2_S_4_, Na_2_S_2_ and Na_2_S based on these obviously more negative E_ads_. Considering the fundamentally competing relationship between adsorption and desorption processes, and to verify the true advantage of Mo₂C materials jointly modified by an appropriate proportion of Fe doping to Mo vacancies, we constructed a model with a higher relative ratio of Mo vacancies to Fe dopant, denoted as vFe-Mo_2_C-1 (Fig. S30). And the corresponding E_ads_ toward S_8_, Na_2_S_8_, Na_2_S_6_, Na_2_S_4_, Na_2_S_2_ and Na_2_S on vFe-Mo_2_C-1 are calculated to be -1.69, -2.91, -4.75, -3.58, -4.35 and -4.65 eV, respectively, which are always lower than those of the vFe-Mo₂C catalyst, indicating a stronger interaction between vFe-Mo₂C-1 and the various sulfur intermediates. However, experimental results show that excessively high relative content of vacancies leads to the electrochemical performance deterioration instead. These suggest that, within a suitable range, more negative binding energies are beneficial for catalytic reactions. On the one hand, sulfur species can be effectively captured by the catalyst to form Mo-S bonds, accompanied by the original S-S bond cleavage that rapidly triggers the conversion reactions. On the other hand, the catalyst-intermediate interaction does not impose a significant barrier for product desorption, thereby enabling a highly efficient catalytic process while avoiding catalyst deactivation. In this work, the moderate interaction between the v_6_Fe-Mo_2_C/C catalyst and sulfur species enables effective adsorption of long-chain NaPSs while allowing the facile desorption of Na_2_S, demonstrating the advantage of Mo_2_C materials jointly modified by an appropriate proportion of Fe doping to Mo vacancies in catalytic activity.


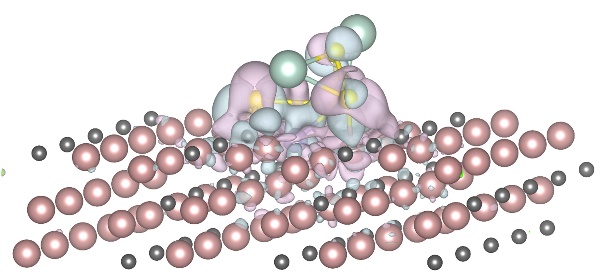


**Fig. S31** Charge density difference of Na_2_S_4_ on Fe-Mo_2_C, blue and purple areas represent increased and decreased electron density, respectively


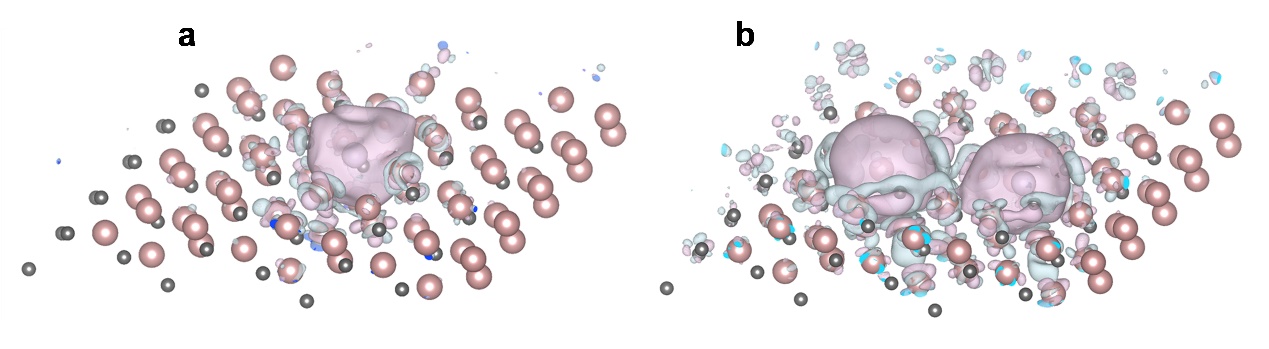


**Fig. S32** Charge density distribution: **a** Fe-Mo_2_C. **b** vFe-Mo_2_C, where blue and purple represent charge accumulation and loss, respectively

**Table S1** Values of Fe doping amount

| Sample | Fe doping amount [wt%] |
| --- | --- |
| **Mo_2_C** | **-** |
| **v_0_Fe-Mo_2_C** | **11.3** |
| **v_2_Fe-Mo_2_C** | **6.42** |
| **v_4_Fe-Mo_2_C** | **3.11** |
| **v_6_Fe-Mo_2_C** | **1.37** |
| **v_12_Fe-Mo_2_C** | **-** |

Data calculated from inductively coupled plasma atomic emission spectroscopy results.

**Table S2** Mo 3d_5/2_ binding energies of Mo_2_C/C and v_t_Fe-Mo_2_C/C (t=0, 2, 4, 6, 12) host materials and the corresponding percentages towards surface compositions of Mo_2_C, Fe_3_Mo_3_C, and Mo-vacancy species

| Host  materials | Mo 3d_5/2_ [eV] | | | Surface Mo species percentage [%] | | |
| --- | --- | --- | --- | --- | --- | --- |
|  | **Mo (2+)**  **(Mo_2_C)** | **Mo (2+**'**) Fe_3_Mo_3_C** | **Mo (3+)**  **Mo vacancies** | **Mo (2+)**  **(Mo_2_C)** | **Mo (2+**'**) Fe_3_Mo_3_C** | **Mo (3+)**  **Mo vacancies** |
| **Mo_2_C** | **229.05** | **-** | **-** | **20.41** | **0** | **0** |
| **v_0_Fe-Mo_2_C** | **228.56** | **229.02** | **-** | **22.80** | **22.77** | **0** |
| **v_2_Fe-Mo_2_C** | **228.46** | **228.97** | **229.77** | **20.54** | **15.01** | **7.18** |
| **v_4_Fe-Mo_2_C** | **228.47** | **228.89** | **229.49** | **20.00** | **8.15** | **14.16** |
| **v_6_Fe-Mo_2_C** | **228.45** | **228.99** | **229.57** | **21.40** | **7.46** | **16.49** |
| **v_12_Fe-Mo_2_C** | **228.52** | **-** | **229.31** | **21.21** | **0** | **21.10** |

**Table S3** Fe 2p_3/2_ binding energies of Mo_2_C/C and v_t_Fe-Mo_2_C/C (t=0, 2, 4, 6, 12) host materials and the corresponding percentages of Fe metal, Fe_3_Mo_3_C, and Fe oxides

| Host  materials | Fe 2p_3/2_ [eV] | | | Surface Fe species percentage [%] | | |
| --- | --- | --- | --- | --- | --- | --- |
|  | **Fe(0)**  **（Fe metal）** | **Fe(+2)**  **（Fe oxide)** | **Fe(+3)**  **（Fe_3_Mo_3_C）** | **Fe(0)**  **（Fe metal）** | **Fe(+2)**  **（Fe oxide)** | **Fe(+3)**  **（Fe_3_Mo_3_C）** |
| **v_0_Fe-Mo_2_C** | **707.87** | **711.67** | **713.58** | **15.26** | **40.38** | **44.37** |
| **v_2_Fe-Mo_2_C** | **707.3** | **711.92** | **713.94** | **3.88** | **43.04** | **53.08** |
| **v_4_Fe-Mo_2_C** | **707.48** | **710.92** | **713.84** | **1.98** | **38.89** | **59.13** |
| **v_6_Fe-Mo_2_C** | **-** | **-** | **713.61** | **0** | **0** | **100** |
| **v_12_Fe-Mo_2_C** | **-** | **-** | **-** | **0** | **0** | **0** |

**Table S4** Fe_surface_, Mo_surface_, Mo_vacancies_ and Mo(v)_surface_ percentages and R values

| Host  materials | Surface species percentage [%] | | | | R^e)^ |
| --- | --- | --- | --- | --- | --- |
|  | **Fe_surface_^a)^** | **Mo_surface_^b)^** | **Mo_vacancies_^c)^** | **Mo(v)_surface_^d)^** |  |
| **v_0_Fe-Mo_2_C** | **3.73** | **6.33** | **-** | **-** | **-** |
| **v_2_Fe-Mo_2_C** | **2.02** | **8.55** | **7.18** | **0.61** | **3.31** |
| **v_4_Fe-Mo_2_C** | **0.68** | **9.77** | **14.16** | **1.38** | **0.49** |
| **v_6_Fe-Mo_2_C** | **0.39** | **11.77** | **16.49** | **1.94** | **0.20** |
| **v_12_Fe-Mo_2_C** | **-** | **12.81** | **21.10** | **2.70** | **-** |

a) Fe_surface_: catalyst surface Fe species percentage, based on XPS full spectrum data.

b) Mo_surface_: catalyst surface Mo species percentage, based on XPS full spectrum data. c) Mo_vacancies_: Mo vacancies percentage in all surface Mo species, based on XPS of Mo 3d.

d) Mo(v)_surface_: catalyst surface Mo vacancies percentage, Mo(v)_surface_ = Mo_surface_ × Mo_vacancies_

e) R = (Fe_surface_/Mo(v)_surface_)

**Table S5** EXAFS data fitting results for various samples at the Fe K-edge

| Sample | Shell | CN^a^ | R(Å)^b^ | σ^2^ (Å^2^)^c^ | ΔE_0_ (eV)^d^ | R factor^e^ |
| --- | --- | --- | --- | --- | --- | --- |
| Fe Foil | **Fe-Fe_1_** | **8*** | **2.47±0.01** | **0.0026** | **4.67** | **0.0068** |
|  | **Fe-Fe_2_** | **6*** | **2.84±0.01** | **0.0023** |  |  |
| Fe_2_O_3_ | **Fe-O** | **6*** | **1.97±0.01** | **0.0104** | **-0.75** | **0.0126** |
|  | **Fe-O-Fe** | **6*** | **2.97±0.01** | **0.0054** |  |  |
| v_6_Fe-Mo_2_C/C | **Fe-C** | **2.8±0.2** | **1.98±0.01** | **0.0078** | **-2.25** | **0.0188** |
|  | **Fe-C-Metal** | **3.1±0.3** | **3.14±0.01** | **0.0032** |  |  |

^a^ CN, coordination number.

^b^ R, bonding distance.

^c^ σ^2^, Debye-Waller factor.

^d^ Δ*E*_0_, inner potential correction.

^e^ R factor indicates the goodness of the fit.

**Table S6** Bandgap and band positions of n-Mo_2_C and pnp-Mo_2_C materials investigated by UV-vis absorption spectra and UPS spectra

| Sample | E_g_ (eV) | E_F_ (eV) | E_VB_ (eV) | E_CB_ (eV) |
| --- | --- | --- | --- | --- |
| **n-Mo_2_C** | **1.183** | **-0.521** | **0.499** | **-0.684** |
| **pnp-Mo_2_C** | **1.118** | **-0.230** | **0.409** | **-0.709** |

**Table S7** A comprehensive comparison of the electrochemical performance among the v_6_Fe-Mo_2_C/C@S cathode and the state-of-the-art cathodes of Na-S battery systems

| Cathode materials | Ratio of sulfur (wt%) | Sulfur loading  (mg cm^-2^) | | Rate capability | | Cycle life under high sulfur loading  (mAh g^-1^@A g^-1^  /cycle NO.) | Energy density  (Wh kg^-1^@A g^-1^) | Refs. |
| --- | --- | --- | --- | --- | --- | --- | --- | --- |
|  |  | **ordinary** | **high** | **Specific capacity**  **(mAh g^-1^@A g^-1^)** | **Capacity retention** |  |  |  |
| v_6_Fe-Mo_2_C/C@S | **48.8%** | **1.0** | **4.1** | **1334.2@1.0**  **1130.5@5.0** | **88.8@1.0**  **75.1@5.0** | **1072.8@0.2/100^th^** | **878.9@0.1/100th**  **701.7@1/1000th** | **This work** |
| CN/Au/S | **56.5%** | **/** | **/** | **599.0@1.0**  **414.0@5.0** | **72.2@1.0**  **49.9@5.0** | **/** | **752.7@0.1** | **[S11]** |
| Zn-N_2_/CF/S film | **/** | **1.0** | **/** | **692.4/@1.0**  **595.3@5.0** | **80.8@1.0**  **69.5@5.0** | **/** | **992.3@0.1 (based on the m_sulfur_)** | **[S12]** |
| ZnS-NC@Ni-N_4_/S | **53.7%** | **1.0** | **2.6** | **924.0@1.0**  **650.0@5.0** | **78.3@1.0**  **55.1@5.0** | **640.0@0.2/200^th^** | **595.2@0.2** | **[S13]** |
| S/MoC-W_2_C-MCNFs | **50.3%** | **0.8** | **1.0** | **790.9@1.0**  **147.0@5.0** | **67.0@1.0**  **12.5@5.0** | **337.6@0.2/75^th^** | **754.22@0.2** | **[S14]** |
|  |  |  | **2.0** |  |  | **456.0@0.5/150^th^** |  |  |
| MMPCS-800@S | **43.8%** | **/** | **/** | **779.0@1.0**  **445.0@5.0** | **70.0@1.0**  **40.0@5.0** | **/** | **673.0@0.1** | **[S15]** |
| CSB@TiO_2_ | **60.0%** | **1.2-1.4** | **/** | **455.0@1.0**  **350.0@2.0** | **67.8@1.0**  **52.2@2.0** | **/** | **405.2@0.5/100^th^** | **[S16]** |
| S@HPC/Mo_2_C | **42.0%** | **/** | **/** | **866.0@1.0**  **679.0@5.0** | **69.6@1.0**  **54.5@5.0** | **/** | **656.0@0.2/120^th^** | **[S17]** |
| Fe-Co/NC/S | **50.0%** | **1.0** | **3.2** | **683.0@1.0**  **337.0@5.0** | **50.5@1.0**  **24.9@5.0** | **~710.0@0.1/70^th^** | **410.4@1.0** | **[S18]** |
|  |  |  | **5.6** |  |  | **280.4@0.1/100^th^** |  |  |
| Fe-N_1_/S | **57.1%** | **1.0** | **/** | **937.4@1.68**  **615.7@5.03** | **72.2@1.68**  **47.4@5.03** | **/** | **676.7@1.675** | **[S19]** |
| S/ELSC-40 | **40.0%** | **1.0** | **/** | **977.7@1.68**  **568.0@5.03** | **72.5@1.68**  **42.1@5.03** | **/** | **524.6@0.335/100^th^** | **[S20]** |
| S@Fe/NC/700 | **46.0%** | **1.5-2.0** | **4-4.5** | **735.0@1.0**  **545.0@5.0** | **71.4%@1.0**  **52.9%@5.0** | **550.0@1.0/150^th^** | **622.9@0.2/100^th^** | **[S21]** |
| S/Mo_2_N-W_2_N@PC | **48.8%** | **0.9** | **2.7** | **619.0@1.0**  **190.0@5.0** | **67.7@1.0**  **20.8@5.0** | **617.0@0.2/80^th^**  **535.0@0.5/100^th^** | **663.22@0.1** | **[S22]** |
| MoC/Mo_2_C  @PCNT-S | **/** | **0.7** | **2.0** | **987.0@1.0**  **621.0@5.0** | **/** | **660.0@0.3/250^th^** | **1240@1.0 (based on the m_sulfur_)** | **[S23]** |

The gravimetric energy density was calculated according to the equation [S24, S25]:

W = (∫ E*Q*m_s_)/M

Where W (Wh kg^-1^), E (V), Q (mAh g^-1^) and M (mg) are the energy density, reversible potential, the specific capacity and the total mass, respectively.

**Supplementary References**

1. A. Meintz, J. Zhang, R. Vijayagopal, C. Kreutzer, S. Ahmed, I. Bloom, A. Burnham, R. B. Carlson, F. Dias, E. J. Dufek, J. Francfort, K. Hardy, A. N. Jansen, M. Keyser, A. Markel, C. Michelbacher, M. Mohanpurkar, A. Pesaran, D. Scoffield, M. Shirk, T. Stephens, T. Tanim. Enabling fast charging-vehicle considerations. J. Power Sources **367**, 216-227 (2017). <https://doi.org/10.1016/j.jpowsour.2017.07.093>
2. Y.-X. Yao, L. Xu, C. Yan, Q. Zhang. Principles and trends in extreme fast charging lithium-ion batteries. EES Batteries. **1**(1), 9-22 (2025). <https://doi.org/10.1039/d4eb00011k>
3. H. Song, K. Munch, X. Liu, K. Shen, R. Zhang, T. Weintraut, Y. Yusim, D. Jiang, X. Hong, J. Meng, Y. Liu, M. He, Y. Li, P. Henkel, T. Brezesinski, J. Janek, Q. Pang. All-solid-state Li-S batteries with fast solid-solid sulfur reaction. Nature **637**(8047), 846-853 (2025). <https://doi.org/10.1038/s41586-024-08298-9>
4. J. Chen, H. Lu, X. Zhang, Y. Zhang, J. Yang, Y. Nuli, Y. Huang, J. Wang. Electrochemical polymerization of nonflammable electrolyte enabling fast-charging lithium-sulfur battery. Energy Storage Mater. **50**, 387-394 (2022). <https://doi.org/10.1016/j.ensm.2022.05.044>
5. C. Zhang, D. Wang, Q. Jin, Y. Tao, T. Qi, Z. Zhang, X. Zhang, L. Wu. Enabling extreme fast‐charging lithium‐sulfur batteries via interfacial electric field engineering on single‐atom‐anchored high‐entropy oxides. Adv. Funct. Mater. e25702 (2025). <https://doi.org/10.1002/adfm.202525702>
6. J. R. González-Jiménez, F. J. Jiménez-Romero, Á. Bonilla, M. C. López-Luna, F. R. Lara-Raya, Á. Caballero. Improved electrochemical performance of fast-charging Li-S batteries with constant power transfer protocol. J. Energy Storage. **86**, 111317 (2024). <https://doi.org/10.1016/j.est.2024.111317>
7. J. Offermann, S. N. Ul Haq, K. X. Wang, R. Adelung, S. H. Chang, B. Babu, M. Abdollahifar. Fast‐charging lithium-sulfur batteries. Adv. Energy Mater. **15**(26), 2404383 (2025). <https://doi.org/10.1002/aenm.202404383>
8. N. Turaeva, G. Yablonsky, R. Fushimi. Fermi-based kinetic model for the sabatier reaction: Sabatier principle and beyond it. J. PHYS. CHEM. C **129**(16), 7730-7739 (2025). <https://doi.org/10.1021/acs.jpcc.5c00099>
9. R. A. Miranda-Quintana, N. Adebar, M. Schulze, J. Smiatek. Sabatier principle revisited: The role of electronic properties in simple catalytic reactions. J. PHYS. CHEM. C **129**(21), 9926-9934 (2025). <https://doi.org/10.1021/acs.jpcc.5c01822>
10. G. Xu, C. Cai, T. Wang. Toward sabatier optimal for ammonia synthesis with paramagnetic phase of ferromagnetic transition metal catalysts. J. Am. Chem. Soc. **144**(50), 23089-23095 (2022). <https://doi.org/10.1021/jacs.2c10603>
11. N. Wang, Y. Wang, Z. Bai, Z. Fang, X. Zhang, Z. Xu, Y. Ding, X. Xu, Y. Du, S. Dou, G. Yu. High-performance room-temperature sodium–sulfur battery enabled by electrocatalytic sodium polysulfides full conversion. Energy Environ. Sci. **13**(2), 562-570 (2020). <https://doi.org/10.1039/c9ee03251g>
12. G. Yao, Z. Li, Y. Zhang, Y. Xiao, L. Wei, H. Niu, Q. Chen, Y. Yang, F. Zheng. Highly flexible carbon film implanted with single‐atomic Zn-N_2_ moiety for long‐life sodium‐sulfur batteries. Adv. Funct. Mater. **34**(5), 2214353 (2023). <https://doi.org/10.1002/adfm.202214353>
13. D. Fang, T. Ghosh, S. Huang, Y. Wang, J. Qiu, X. Xu, H. Y. Yang. Core-shell tandem catalysis coupled with interface engineering for high-performance room-temperature Na-S batteries. Small. **19**(41), e2302461 (2023). <https://doi.org/10.1002/smll.202302461>
14. S. Zhang, M. Huang, Y. Wang, Z. Wang, H. Wang, X. Liu. Achieving a quasi‐solid‐state conversion of polysulfides via building high efficiency heterostructure for room temperature Na-S batteries. Adv. Energy Mater. **14**(14), 2303925 (2024). <https://doi.org/10.1002/aenm.202303925>
15. C. Wu, Y. Lei, L. Simonelli, D. Tonti, A. Black, X. Lu, W. H. Lai, X. Cai, Y. X. Wang, Q. Gu, S. L. Chou, H. K. Liu, G. Wang, S. X. Dou. Continuous carbon channels enable full na-ion accessibility for superior room-temperature Na-S batteries. Adv. Mater. **34**(8), e2108363 (2022). <https://doi.org/10.1002/adma.202108363>
16. D. Ma, Y. Li, J. Yang, H. Mi, S. Luo, L. Deng, C. Yan, M. Rauf, P. Zhang, X. Sun, X. Ren, J. Li, H. Zhang. New strategy for polysulfide protection based on atomic layer deposition of TiO_2_ onto ferroelectric‐encapsulated cathode: Toward ultrastable free‐standing room temperature sodium-sulfur batteries. Adv. Funct. Mater. **28**(11), 1705537 (2018). <https://doi.org/10.1002/adfm.201705537>
17. X. Zhou, Z. Yu, Y. Yao, Y. Jiang, X. Rui, J. Liu, Y. Yu. A high-efficiency Mo_2_C electrocatalyst promoting the polysulfide redox kinetics for na-s batteries. Adv. Mater. **34**(14), e2200479 (2022). <https://doi.org/10.1002/adma.202200479>
18. C. Li, J. Yu, D. Yang, H. Li, Y. Cheng, Y. Ren, X. Bi, J. Ma, R. Zhao, Y. Zhou, J. Wang, C. Huang, J. Li, I. Pinto-Huguet, J. Arbiol, H. Zhang, S. Xin, A. Cabot. Balancing electronic spin state via atomically-dispersed heteronuclear Fe-Co pairs for high-performance sodium-sulfur batteries. J. Am. Chem. Soc. **147**(10), 8250-8259 (2025). <https://doi.org/10.1021/jacs.4c15408>
19. W. Song, Z. Wen, X. Wang, K. Qian, T. Zhang, H. Wang, J. Ding, W. Hu. Unsaturation degree of fe single atom site manipulates polysulfide behavior in sodium-sulfur batteries. Nat. Commun. **16**(1), 2795 (2025). <https://doi.org/10.1038/s41467-025-58114-9>
20. D. Zhao, S. Jiang, S. Yu, J. Ren, Z. Zhang, S. Liu, X. Liu, Z. Wang, Y. Wu, Y. Zhang. Lychee seed-derived microporous carbon for high-performance sodium-sulfur batteries. Carbon **201**, 864-870 (2023). <https://doi.org/10.1016/j.carbon.2022.09.075>
21. J. Ruan, Y. J. Lei, Y. Fan, M. C. Borras, Z. Luo, Z. Yan, B. Johannessen, Q. Gu, K. Konstantinov, W. K. Pang, W. Sun, J. Z. Wang, H. K. Liu, W. H. Lai, Y. X. Wang, S. X. Dou. Linearly interlinked Fe-N_x_-Fe single atoms catalyze high-rate sodium-sulfur batteries. Adv. Mater. **36**(21), e2312207 (2024). <https://doi.org/10.1002/adma.202312207>
22. S. Zhang, Y. Yao, X. Jiao, M. Ma, H. Huang, X. Zhou, L. Wang, J. Bai, Y. Yu. Mo_2_N-W_2_N heterostructures embedded in spherical carbon superstructure as highly efficient polysulfide electrocatalysts for stable room‐temperature na–s batteries. Adv. Mater. **33**(43), 2103846 (2021). <https://doi.org/10.1002/adma.202103846>
23. H. Hao, Y. Wang, N. Katyal, G. Yang, H. Dong, P. Liu, S. Hwang, J. Mantha, G. Henkelman, Y. Xu, J. A. Boscoboinik, J. Nanda, D. Mitlin. Molybdenum carbide electrocatalyst in situ embedded in porous nitrogen-rich carbon nanotubes promotes rapid kinetics in sodium-metal-sulfur batteries. Adv. Mater. **34**(26), e2106572 (2022). <https://doi.org/10.1002/adma.202106572>
24. C. Ma, X. Wang, J. Lan, J. Zhang, K. Song, J. Chen, J. Ge, W. Chen. Dynamic multistage coupling of FeS_2_/S enables ultrahigh reversible Na-S batteries. Adv. Funct. Mater. **33**(5), 2211821 (2022). <https://doi.org/10.1002/adfm.202211821>
25. J. Huang, X. Guo, X. Du, X. Lin, J.-Q. Huang, H. Tan, Y. Zhu, B. Zhang. Nanostructures of solid electrolyte interphases and their consequences for microsized sn anodes in sodium ion batteries. Energy Environ. Sci. **12**(5), 1550-1557 (2019). <https://doi.org/10.1039/c8ee03632b>
